# Lipidome of the Brown Macroalga *Undaria pinnatifida*: Influence of Season and Endophytic Infection

**DOI:** 10.3390/md21090466

**Published:** 2023-08-25

**Authors:** Ksenia Chadova, Peter Velansky

**Affiliations:** A.V. Zhirmunsky National Scientific Center of Marine Biology, Far Eastern Branch of Russian Academy of Sciences, Vladivostok 690041, Russia; velansky.pv@gmail.com

**Keywords:** brown algae, lipidome, seasonal adaptation, endophytic infection

## Abstract

An analysis of the lipidome of the brown alga *Undaria pinnatifida* (Laminariales) was performed’ more than 900 molecular species were identified in 12 polar lipids and 1 neutral lipid using HPLC/MS-MS. The seasonal changes of *U. pinnatifida* lipidome were determined. It was shown that acclimatization to winter and spring was accompanied by an increase in the unsaturation of both polar and neutral lipids. In autumn and summer, on the contrary, the contents of more saturated molecular species of all lipid classes increased. Based on the data obtained, a scheme for the polar and neutral lipid synthesis in brown algae was proposed. In addition, the influence of infection with the brown filamentous endophyte *Laminariocolax aecidioides* (Ectocarpales) on *U. pinnatifida* lipidome was studied. It was found that infection has the most noticeable effect on the molecular species composition of triacylglycerides, phosphatidylglycerol, phosphatidylcholine, phosphatidylethanolamine, and phosphatidylhydroxyethylglycine of the host macrophyte. In infected samples of algae, changes in the composition of triacylglycerides were revealed both in areas with the presence of an endophyte and in adjacent intact tissues, which may indicate the occurrence of a secondary infection.

## 1. Introduction

*Undaria pinnatifida* (Harvey) Suringar, 1873 (Phaeophyceae: Laminariales) is an annual brown macrophyte growing in littoral and sublittoral zones at a depth of 1–15 m. In many Asian countries, including Japan, China and Korea, these algae are cultivated and used as a food additive [1]. In Russia, a population of *U. pinnatifida* was found in Peter the Great Bay (Sea of Japan) [2]. It is known that environmental factors affect the growth, development, and photosynthetic activity of algae [3]. In the course of evolution, algae have developed numerous compensatory mechanisms to smooth out their negative effects. Lipid metabolism is one of the key mechanisms in the system of algae’s adaptation to changing environmental conditions. Modifications of the lipid composition of algae, mediated by changes in environmental conditions, such as temperature, light level and availability of nutrients, are aimed at maintaining physiological processes at an optimal level [4]. Previously, a comparative analysis of the total fatty acid (FA) composition and the content of lipid classes in *U. pinnatifida* was carried out in different seasons [5,6], but since the FA composition and the change in their degree of unsaturation during adaptation to external environmental conditions are specific for individual lipid classes, a comprehensive analysis of changes within each class is needed. The determination of the seasonal dynamics of the *U. pinnatifida* lipid molecular species can provide a complete picture of the cell membrane’s adaptive reorganization. In this regard, the purpose of this study was to analyze changes in the composition of lipid classes, as well as the composition of lipid molecular species in *U. pinnatifida* samples collected in different months.

When studying samples of *U. pinnatifida*, anomalies in the form of dark spots and perforations were found on the algal blade. An earlier microscopic examination showed that *U. pinnatifida* samples were infected with the brown endophytic algae *Laminariocolax aecidioides* (Rosenvinge) A.F. Peters, 1998 (Phaeophyceae: Ectocarpales), while the proportion of infected algae was 66–100% [7]. The authors found that infection occurs at the early stages of macrophyte growth in late October–early November, while the maximum development of the endophyte is observed in June–July during the period of sporulation and the natural destruction of thalli. Gauna et al. [8] found that the penetration of *L. aecidioides* filaments into the *U. pinnatifida* tissue causes lateral cell compression, and the development of their thalli leads to the formation of perforations in the macrophyte tissue. Currently, studies devoted to the analysis of the algae’s protective reactions to endophytic infection are quite common. It has recently been shown that the co-cultivation of the brown endophytic algae *Laminarionema elsbetiae* (Ectocarpales) and brown macrophytic algae is accompanied by the activation of several protective genes, including those encoding the oxidase homologue protein, which mediates the oxidative burst, and proteins with lipase and lipoxygenase activities, as well as the suppression of the expression of genes associated with photosynthesis and FA synthesis [9]. Studies on other algae produced similar results [10,11], but only one of them used a lipidomic approach [12]. In our previous study on juvenile *U. pinnatifida*, we showed that infection with *L. aecidioides* has the most significant effect on the FA composition of the neutral lipid fraction [13]. In this work, for a more detailed assessment of the influence of *L. aecidioides* on the *U. pinnatifida*, we carried out a comparative analysis of the lipidomes of infected and intact algae samples collected at an early stage of development and during sporulation.

## 2. Results and Discussion

### 2.1. Lipid Composition of Undaria pinnatifida

#### 2.1.1. Lipid Classes’ Composition of *Undaria pinnatifida*

A total of 12 polar (structural) lipids were identified, including glycoglycerolipids (GL)-monogalactosyldiacylglycerol (MGDG), digalactosyldiacylglycerol (DGDG), sulfoquinovosyldiacylglycerol (SQDG), and glucuronosyldiacylglycerol (GlcADG), phosphoglycerolipids (PL)-phosphatidylglycerol (PG), phosphatidylethanolamine (PE), phosphatidylcholine (PC), phosphatidylhydroxyethylglycine (PHEG), phosphatidylinositol (PI), phosphosphingolipid-ceramide phosphoinositol (CPI), and betaine lipids (BL)-diacylglyceryltrimethylhomoserine (DGTS) and diacylglycerylhydroxymethyltrimethylalanine (DGTA). Neutral (storage) lipids were represented by triacylglycerides (TAGs) (Table S1). GL and PG are components of plastidic (chloroplast) membranes, while PC, PE, PI, PHEG, and BL are components of extraplastidic membranes, such as plasma, mitochondrial, etc. PG can also be contained in extraplastidic membranes in small amounts.

**Figure 1:**
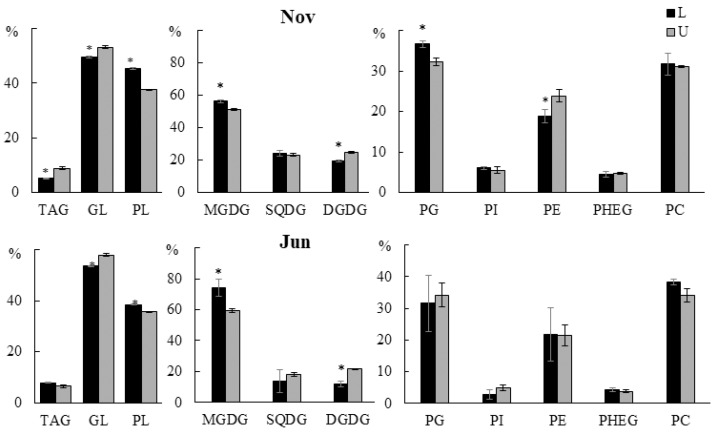
The contents of triacylglycerides (TAG), glycolipids (GL), and phospholipids (PL) (% of the total lipids) and the main classes of GL and PL (% of total GL and PL classes, respectively) of the lower (L) and upper (U) parts of the blade of *U. pinnatifida* samples collected in November and June. An asterisk indicates significant differences from the subsequent data point (*t*-test, *p* < 0.05).

GLs prevailed in all parts of the algal blade, while their total content in the lower parts was less than in the upper ones (52.2% vs. 58.2% in November, 58.3% vs. 61.9% in June (% of the total PL)) (Figure 1). In the lower parts, a higher content of MGDG was detected (56.0% vs. 51.4% in November and 74.2% vs. 59.3% in June (% of the total GL)). The content of DGDG, on the contrary, was lower in the lower parts (19.2% vs. 24.3% in November, 11.8% vs. 22.0% in June (% of the total GL)). Similar results were obtained in a study conducted on another brown alga *Laminaria japonica* (Laminariales) [14], which, like *U. pinnatifida*, is characterized by intercalary growth (blade growth occurs due to the intercalary meristem located at its base). The authors suggested that such differences may be related both to the age of the algae tissues (the lower part of the thalli is younger, the upper part is older) and to the functional features of these lipids. Old tissues are characterized by a higher intensity of photosynthesis, and, accordingly, an increased content of GLs in general, and DGDG in particular. The contents of PG (31.5–36.7% of the total PL), PC (31.1–38.3% of the total PL), PE (18.8–21.4% of the total PL), and PHEG (3.8–4.7% of the total PL) did not differ in different parts of the algal blade. CPI and BL were detected in trace amounts (less than 0.1%). The level of TAG ranged from 5.3% to 9.7% of the total lipids. It should be noted that we did not detect significant amounts of polar lipid lysoforms.

CPI was previously detected in brown algae in only one study [15]; this lipid is typical for red algae, as well as fungi and protozoa [16,17,18,19]. The question of its origin remains open; CPI can be either an endogenous lipid of brown algae or a sign of the presence of epi- or endophytic fungi or red algae. Betaine lipids DGTA and DGTS in algae of the order Laminariales were previously identified only in a few studies in trace amounts [20,21,22]. GlcADG is common in higher plants [23], unicellular algae [24], sea grasses [25], as well as in some bacteria and fungi [26]. Only in recent studies was this lipid identified in the brown algae *U. pinnatifida* and *S. natans* [21]. It is known that, in higher plants, GlcADG is synthesized in plastids from UDP-glucuronic acid and diacylglycerol (DAG) using SQDG synthase, and has an FA composition similar to that of SQDG [23]. In *U. pinnatifida*, the molecular composition of GlcADG was similar to that of SQDG. The function of GlcADG is not clear; it is only known that it can play a role in phosphorus starvation [27].

#### 2.1.2. Fatty Acids Composition of *Undaria pinnatifida*

As a result of the analysis, 47 FAs were identified in the polar (PLF) and neutral (NLF) lipid fractions (Table S2). The main FAs of *U. pinnatifida* were 14:0, 16:0, 18:0, 18:1ω9, 18:2ω6, 18:3ω3, 18:4ω3, 20:4ω6 and 20:5ω3, which is consistent with previous studies [28]. Odd-carbon saturated FAs (SFAs) such as 13:0, 15:0 and 17:0 were found in minor amounts predominantly in the neutral lipid fraction (Table S2).

**Figure 2:**
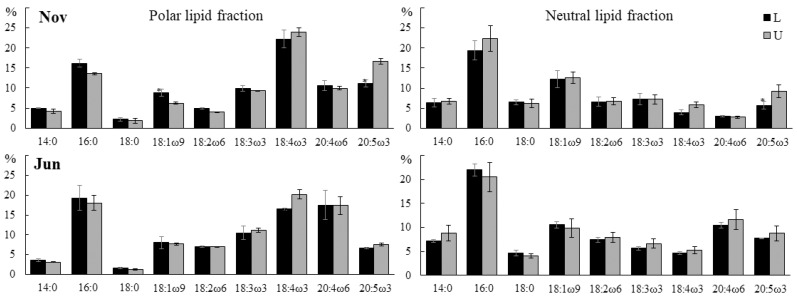
The fatty acid (FA) content of polar and neutral lipid fractions (% of the total FA) in the lower (L) and upper (U) parts of the blade of *U. pinnatifida* samples collected in November and June. An asterisk indicates significant differences from the subsequent data point (*t*-test, *p* < 0.05).

In juvenile algae in the upper parts of the blade, we found a higher content of 20:5ω3 (16.7% vs. 11.1% in PLF, 9.2 vs. 5.7% in NLF) and lower proportions of 16:0 and 18:1ω9 in the PLF (13.6% vs. 16.2% and 6.3% vs. 8.8%, respectively) (Figure 2). In adult algae, the content of 18:4ω3 in the PLF in the upper part of the blade was higher than in the lower part (20.2% vs. 16.5%). An increased amount of C18- and C20-polyunsaturated FA (PUFAs) in the upper parts of the blade may be associated with more active photosynthesis [14].

#### 2.1.3. Lipidome of *Undaria pinnatifida*

More than 900 lipid molecular species were identified in 12 polar lipids and 1 neutral lipid (Table S3). A quantitative calculation of the minor lipid molecular species (GlcADG, DGTA, DGTS, and CPI) was not carried out due to their low content (less than 0.1% of the total polar lipids). The main lipid molecular species are shown in Table 1.

The MGDG and DGDG composition was dominated by the SFA/C18-PUFA, C18-PUFA/C18-PUFA, and C20-PUFA/C18-PUFA molecular species. The SQDG and GlcADG main molecular species contained 14:0, 16:0, 16:1, and C18 FAs with varying degrees of saturation. C18 FAs with varying degrees of saturation, as well as 16:0 and 16:1, dominated the PG molecular species composition. The PE and PC main molecular species were PUFA/C20-PUFA and C20-PUFA/C20-PUFA. PI contained predominantly 14:0 and 16:0 in the sn-1 position and C18 FAs with varying degrees of saturation in the sn-2 position. PHEG is characterized by the presence of two main molecular species—20:4/20:4 and 20:4/20:5, totaling more than 98%. DGTA contained mainly 14:0, 18:4, and 20:5, while DGTS contained 14:0, 16:0, and C18-PUFAs. The acyl part of CPI is presented mainly by 14:0 and 16:0, while the sphingosine bases are presented by 22:0d and 22:1d. TAGs contained SFAs, MUFAs, and PUFAs with different chain lengths and degrees of unsaturation.

**Figure 3:**
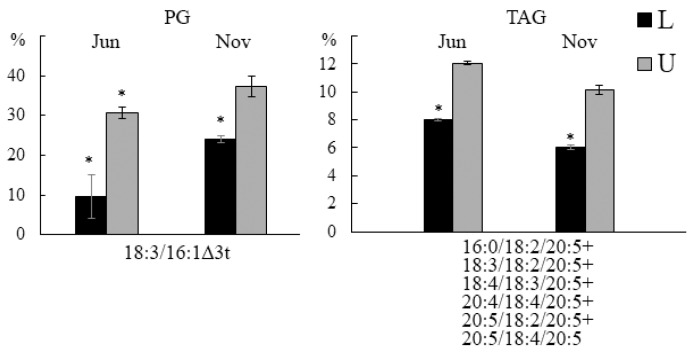
The contents of phosphatidylglycerol (PG) and TAG molecular species (% of the total lipid molecular species) of the lower (L) and upper (U) parts of the blade of *U. pinnatifida* samples collected in November and June. An asterisk indicates significant differences from the subsequent data point (*t*-test, *p* < 0.05).

A comparative quantitative analysis of molecular species revealed some differences between different parts of the *U. pinnatifida* blade (Figure 3). Thus, the 18:3/16:1∆3t PG molecular species’ proportion was higher in the upper part of the algal blade compared to the lower one, which may be due to the more developed structure of plastids in the old part of the thalli, and, accordingly, a large amount of light-harvesting complexes II (LHC II), which are surrounded by PG molecules containing the 16:1∆3t PG-specific FA in the sn-2 position [29]. The level of TAG containing C18- and C20-PUFAs in the upper part was higher than in the lower part, which, apparently, is due to more active photosynthesis.

### 2.2. Seasonal Dynamics of the Lipid Composition of Undaria pinnatifida

The lower (H) and upper tissue sections of U. pinnatifida samples collected in November, December, January, February, April, and June were used for analysis.

#### 2.2.1. Lipid Classes Composition

**Figure 4:**
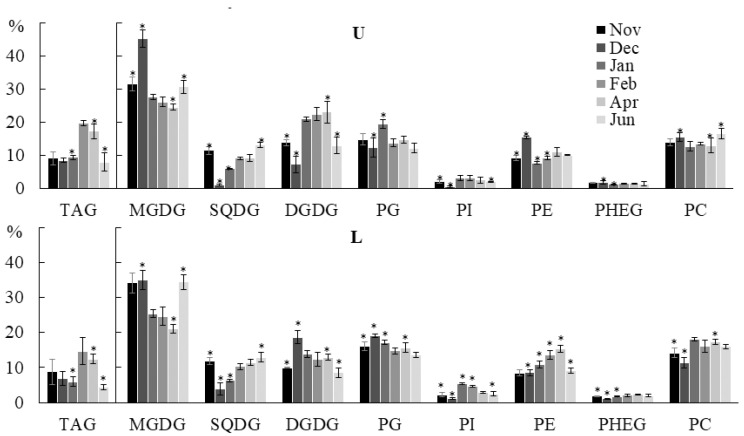
The contents of the main classes of PL (% of the total PL) and TAG (% of the total lipids) in the lower (L) and upper (U) parts of the blades of *U. pinnatifida* samples collected in different months. An asterisk indicates significant differences from the subsequent data point. An asterisk above the last data point indicates a significant difference between June and February (HSD-test, *p* < 0.05).

The *U. pinnatifida* population in the Peter the Great Bay of the Sea of Japan is represented by individuals at different stages of the life cycle: juvenile algae in autumn, winter, and early spring; juvenile and adult algae in late May–early June; and adult fertile algae in June. The compositions and contents of lipids in algae change significantly, which is due to both the ontogenesis stage and various environmental factors, including temperature and light conditions, the concentration of oxygen and nutrients, etc. Our study showed significant fluctuations in the contents of individual lipid classes. Thus, the TAG contents in the lower parts of the blade decreased from November to January (in the upper part they remained constant at that time), then sharply increased in both parts in February, and decreased to the minimum values by June (Figure 4). The decrease in the TAG content in the lower part by January can be explained by their use to maintain intercalary growth in low light conditions. In February, due to the lengthening of the photoperiod, photosynthesis intensifies, but the oxygen lack caused by the presence of ice cover does not allow the full use of photosynthesis products for growth, which leads to TAG accumulation. After the ice melts in March, the metabolic rate increases and the TAG level begins to decrease.

An increase in the MGDG content was noted in December and June in the upper parts of algae blade (Figure 4). The DGDG content in the lower part of the blade in December was the maximum (18.7%), and in November and June it was the minimum (9.9% and 8.5%, respectively). In the upper part of the blade, on the contrary, the DGDG proportion was minimal in December (7.2%), and maximal in April (23.0%). The SQDG level decreased from November to December and then gradually increased towards the summer. The maximum PG content in the lower parts of algal blade was observed in December (19.1%), and in the upper part in January (19.4%). The study conducted on filamentous brown algae of the genus *Streblonema* showed that with increasing temperature and light intensity, the relative content of SQDG increases, while those of MGDG and PG decrease. MGDG is a highly unsaturated non-bilayer lipid and is associated with photosystem proteins, while saturated SQDG is associated with the photosynthetic apparatus to a lesser extent [30,31]. Thus, in December, at the lowest illumination (due to a short day and the onset of water freezing), the SQDG content is the lowest, and MGDG is the highest, which is functionally important for maintaining the bilayer structure of membranes at a high content of photosynthetic proteins and low temperature.

In the lower part of the algal blade, the PE level increased from November to April (from 8.5% to 15.3%), then decreased in June to 9.1%. The PC and PHEG contents in the lower part of the blade were minimal in December. In the upper parts of the algal blade, PE, PC, and PHEG dynamics were hardly noticeable. The contents of GlcADG, CPI, DGTA, and DGTS minor lipids did not depend on the month of collection samples (Table S1).

#### 2.2.2. Molecular Species Composition

**Figure 5:**
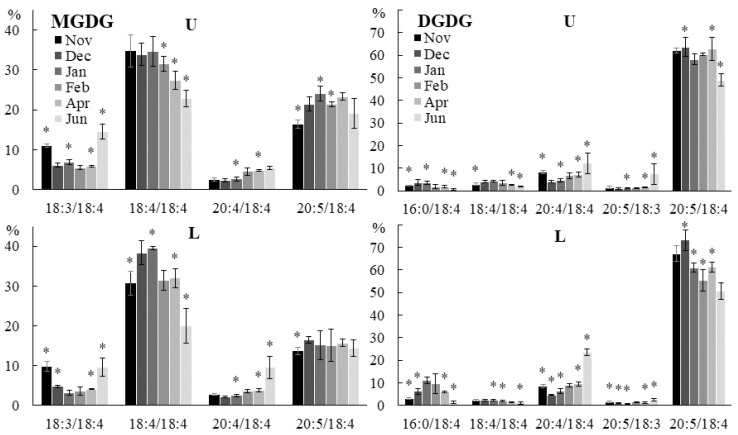
The contents of the monogalactosyldiacylglycerol (MGDG) and digalactosyldiacylglycerol (DGDG) molecular species (% of the total MGDG and DGDG molecular species, respectively) in the lower (L) and upper (U) parts of the blades of *U. pinnatifida* samples collected in different months. An asterisk indicates significant differences from the subsequent data point. An asterisk above the last data point indicates a significant difference between June and February (HSD-test, *p* < 0.05).

One of the most studied adaptation mechanisms of organisms to environmental factors is a change in the unsaturation level of membrane lipids, aimed at maintaining the optimal level of membrane fluidity, by regulating the desaturase activity [32]. However, the analysis of common FA compositions is not informative enough. For a better understanding of the adaptation process, it is necessary to study the change in the unsaturation degree and the acyl group positional distribution within each lipid class. In *U. pinnatifida* samples collected in different months, significant changes were observed in the molecular species compositions of both plastidic (MGDG, DGDG, SQDG, PG) and extraplastidic (PE, PC, PHEG) lipids (Table S3). In different parts of the algal blade, changes in the composition of the same class of lipids were not always identical. Thus, in the lower part of the algal blade in winter and spring, the content of the 18:4/18:4 MGDG molecular species increased, and in the upper part of the blade, the proportion of the 20:5/18:4 molecular species increased (Figure 5). The content of the 18:3/18:4 MGDG molecular species was higher in autumn and summer compared to winter and spring in all parts of the blade. The total content of PUFA/PUFA MGDG molecular species in all parts of algal blade was higher in summer than in autumn (Table S3). This may be due to a more developed plastid structure in adult algae, an increase in the photosynthetic activity of algae associated with a relatively high level of solar radiation in summer, and also to the MGDG function in the violaxanthin cycle, which protects the photosynthetic apparatus from excess energy during increased insolation [33]. The proportion of 20:4/18:4 DGDG increased in autumn and summer, and 18:4/18:4 and 16:0/18:4 increased in winter (Figure 5). An increase in the content of GLs containing highly unsaturated FAs (18:4 and 20:5) at low environmental temperatures in winter and less unsaturated forms of these lipids in autumn and summer may be necessary to maintain the liquid-crystalline phase of thylakoid membranes, and accordingly photosynthesis activity, at an optimal level [34]. At low environmental temperatures, a high degree of plastidic lipid unsaturation is also important for photosystem II (PS II) repair [35]. An increase in the content of highly unsaturated MGDG molecular species may be associated with a reduced level of illumination due to the short duration of daylight hours in winter. A similar adaptive response in brown algae of the genus *Streblonema* cultivated at low light intensity was described [36].

**Figure 6:**
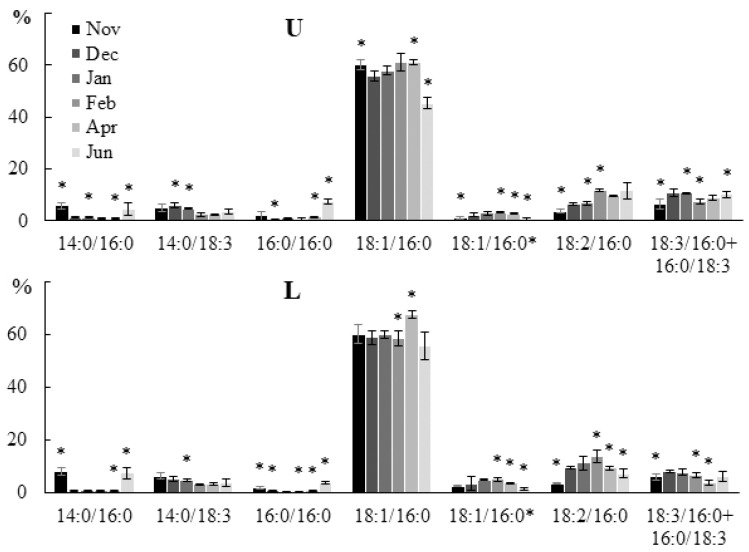
The content of the sulfoquinovosyldiacylglycerol (SQDG) molecular species (% of the total SQDG molecular species) in the lower (L) and upper (U) parts of the blade of *U. pinnatifida* samples collected in different months. 18:1/16:0* is an isomer of the main molecular species 18:1/16:0, apparently differing in the location of the double bond in the 18:1. An asterisk indicates significant differences from the subsequent data point. An asterisk above the last data point indicates a significant difference between June and February (HSD-test, *p* < 0.05).

Changes in the molecular species composition of SQDG were the least noticeable compared to other plastidic lipids. In brown algae, SQDG is usually the most saturated glycolipid. The ratio of saturated and unsaturated FAs in structural lipids is a key factor determining the viscosity of cell membranes. The observed increase in the content of SQDG molecular species with SFA (14:0/16:0, 16:0/16:0) in autumn and summer (Figure 6) can compensate for the high proportion of highly unsaturated MGDG and DGDG, and provide an optimal level of viscosity of thylakoid membranes in this season. A trend towards an increase in the level of SQDG with PUFAs in winter and spring was observed.

**Figure 7:**
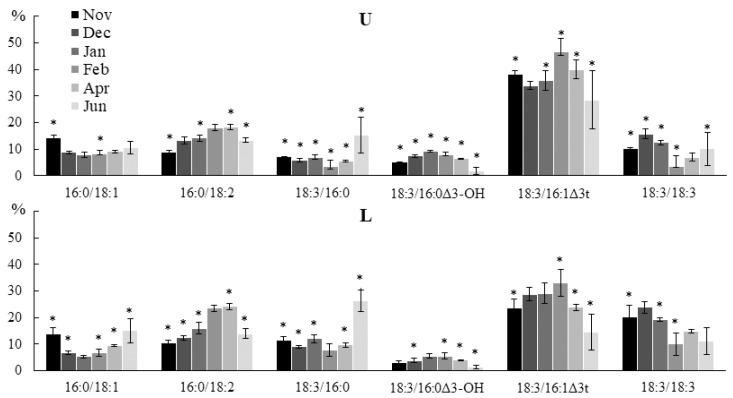
The content of the PG molecular species (% of the total PG molecular species) in the lower (L) and upper (U) parts of the blades of *U. pinnatifida* samples collected in different months. An asterisk indicates significant differences from the subsequent data point. An asterisk above the last data point indicates a significant difference between June and February (HSD-test, *p* < 0.05).

The 18:3/16:1Δ3t PG molecular species level was highest in February, while the 18:3/16:0 level was highest in June (Figure 7). The higher content of the 18:3/16:1Δ3t PG in winter compared to summer is probably due to the low dose of solar radiation during this period. It is known that PG containing 16:1Δ3t FA, which is specific for this phospholipid, in the sn-2 position enhances the trimerization of light-harvesting complex II (LHC II), and thus increases the efficiency of light absorption and energy transfer to photosystems [37]. The 18:3/16:0Δ3-OH molecular species was found in PG, the content of which also increased in the winter–spring period in all parts of the algal blade. It is possible that the 16:0Δ3-OH FA is formed during the oxidation of 16:1Δ3t and is of endogenous rather than bacterial origin. This is supported by the fact that other bacterial FAs (Δ2-OH, Δ3-OH, branched FAs) were not found in comparable amounts. In addition, the 18:3/16:1Δ3-OH PG molecular species, a presumed product of incomplete oxidation, was found in trace amounts (Table S3). It can be assumed that such an intense hydroxylation of 16:1Δ3t is affected either by the position of the double bond, as close as possible to the hydrophilic region of the membrane, by the proximity of PG with this acid to LHC II, or by both of these factors. The content of 16:0/18:1 PG increased in autumn and summer, while the level of 16:0/18:2 increased in winter and spring. In autumn and summer, the contents of SFA/SFA and SFA/MUFA PG molecular species increased, while the content of SFA/PUFA decreased (Table S3). An increase in the proportion of PG with PUFAs of 18:2 and 18:3 in the winter months is due to the fact that unsaturated PG is necessary to maintain the rate of PS II repair during low-temperature photoinhibition [38].

**Figure 8:**
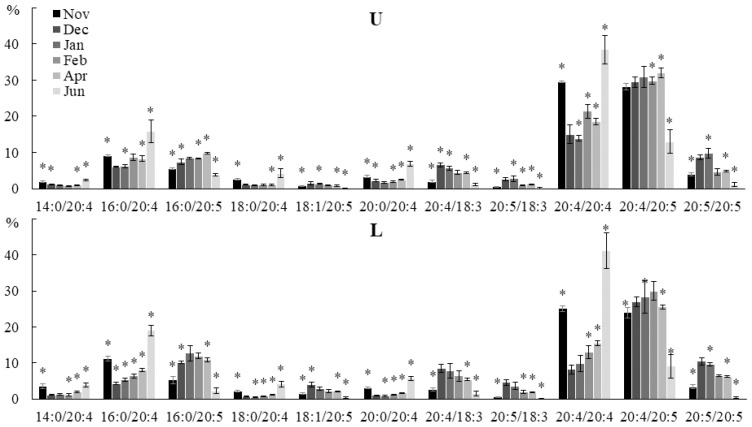
The content of the phosphatidylethanolamine (PE) molecular species (% of the total PE molecular species) in lower (L) and upper (U) parts of the blades of *U. pinnatifida* samples collected in different months. An asterisk indicates significant differences from the subsequent data point. An asterisk above the last data point indicates a significant difference between June and February (HSD-test, *p* < 0.05).

PC, PE, and PHEG are lipids of extraplastidic membranes of brown algae that perform a structural function. In general, the seasonal dynamics of the molecular species contents of these lipids were similar in all parts of the algal blade. The proportion of PE molecular species containing FA 20:4 (14:0/20:4, 16:0/20:4, 18:0/20:4, 20:0/20:4, 20:4/20:4) increased in autumn and summer in all parts of the blade, while the level of molecular species containing FAs with 20:5 FA (16:0/20:5, 18:1/20:5, 20:4/18:3, 20:5 /18:3, 20:5/20:5) was at the maximum in winter (Figure 8). The content of 20:4/20:5 PE decreased significantly only in summer.

**Figure 9:**
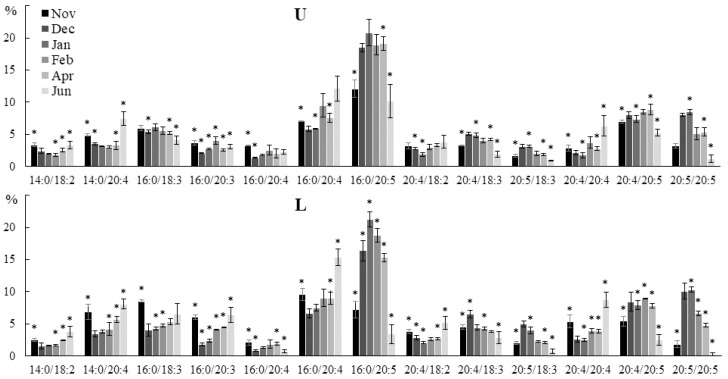
The content of the phosphatidylcholine (PC) molecular species (% of the total PC molecular species) in the lower (L) and upper (U) parts of the blades of *U. pinnatifida* samples collected in different months. An asterisk indicates significant differences from the subsequent data point. An asterisk above the last data point indicates a significant difference between June and February (HSD-test, *p* < 0.05).

A similar but more complex trend was observed in PC (Figure 9). Thus, the contents of 14:0/18:2, 14:0/20:4 16:0/20:3, 20:4/18:2, and 20:4/20:4 molecular species increased in autumn and summer, and the levels of 20:4/18:3, 16:0/20:5, and 20:5/20:5 increased in winter. The contents of 16:0/18:3 and 20:4/20:3 molecular species increased in autumn and summer only in the lower part of the blade. The contents of PUFA/PUFA PC and PE molecular species were lower in summer compared to winter, and that of PUFA/PUFA was higher (Table S3).

**Figure 10:**
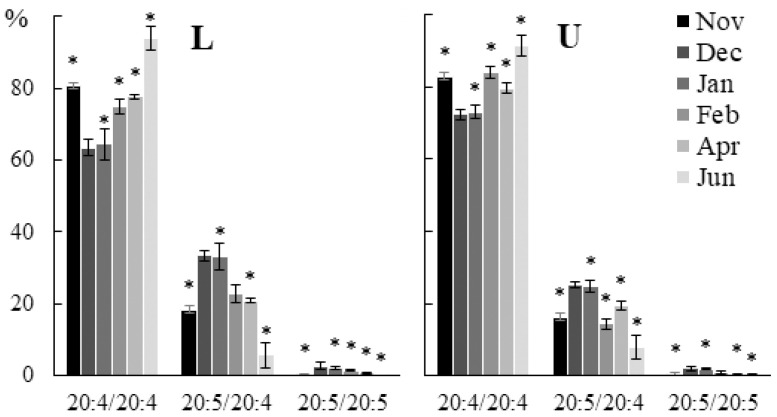
The content of phosphatidylhydroxyethylglycine (PHEG) molecular species (% of the total PHEG molecular species) in the lower (L) and upper (U) parts of the blades of *U. pinnatifida* samples collected in different months. An asterisk indicates significant differences from the subsequent data point. An asterisk above the last data point indicates a significant difference between June and February (HSD-test, *p* < 0.05).

The contents of 20:5/20:4 and 20:5/20:5 PHEG molecular species increased in winter, while the level of 20:4/20:4 was at its maximum in autumn and summer in all parts of the algal blade (Figure 10).

From the point of view of algal physiology, it is well known that the level of unsaturation of membrane lipids increases in response to lower temperatures in order to maintain the fluidity of cell membranes at an optimal level. Changes in the ratio of 20:4 and 20:5 in PC, PE and PHEG, as well as the ratio of 18:2 and 18:3 in the sn-2 position of PC and PE at different temperatures, can also be associated with the regulation of the ratio of ω3/ω6 FAs in structural lipids. It has been shown that brown algae produce the maximum amount of ω6 PUFA during warm months and ω3 PUFA during cold months [39].

The PI molecular composition remained practically unchanged in different months (Table S3). PI is a conservative lipid whose structural role (and, accordingly, the regulation of the physicochemical properties of membranes by changing the composition of FAs) is not the main one. PI is a precursor of a number of phosphoinositides involved in signaling processes, vesicular transport, and cytoskeletal reorganization [40].

**Figure 11:**
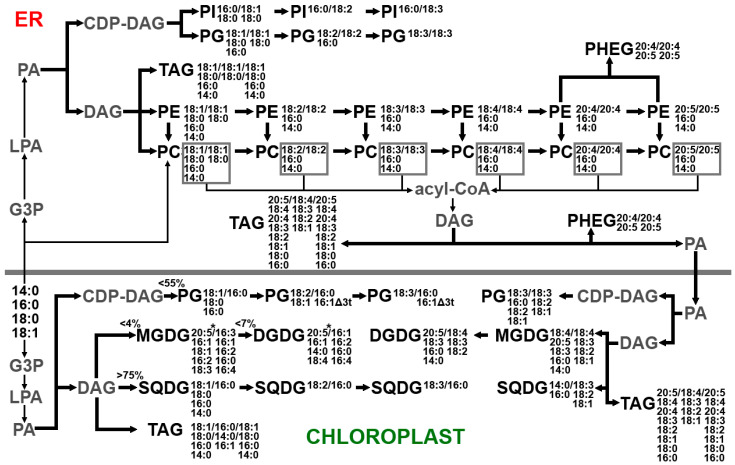
Biosynthesis of lipids in brown algae. CDP-DAG, cytidine diphosphate diacylglycerol; DAG, diacylglycerol; DGDG, digalactosyldiacylglycerol, ER, endoplasmic reticulum; G3P, glycerol-3-phosphate; LPA, lysophosphatidic acid; MGDG monogalactosyldiacylglycerol; PA, phosphatidic acid; PC, phosphatidylcholine; PE, phosphatidylethanolamine; PG, phosphatidylglycerol; PHEG, phosphatidylhydroxyethylglycine; PI, phosphatidylinositol; SQDG, sulfoquinovosyldiacylglycerol; TAG, triacylglyceride. Numbers C:db indicates the number of carbon atoms (C) and double bonds (db) in the fatty acid chains. Intermediates are indicated in gray font. Thick arrows indicate the synthesis of lipid molecules, thin arrows indicate the transfer of fatty acids. 20:5 *—ER-derived fatty acid included in the plastid-derived lipid.

It is known that, in plant and algae cells, DAG assembly occurs both in the endoplasmic reticulum (ER) (eukaryotic pathway) and in plastids (prokaryotic pathway), while *de novo* FA synthesis occurs only in plastids. In plants, for the synthesis of DAGs in the ER, free FAs are transported from plastids via the FAX-1 protein complex [41]. For algae, there is currently no direct evidence of how FAs synthesized in the stroma pass through two, three, and sometimes four plastidic membranes. Most of the FA transported to the ER is directly and reversibly incorporated into PC by acyl-CoA:lyso-PC acyltransferase, where it undergoes elongation and desaturation, and can be released and used for the synthesis of other lipids [42]. DAG molecules synthesized in the ER, presumably in the form of phosphatidic acid, can be transported into the plastidic membrane by the TGD1-5 protein complex and used for the synthesis of thylakoid lipids [43,44]. On the other hand, PC contained in the outer membrane of plastids can be a DAG donor for the synthesis of plastidic lipids. The synthesis of C20 FAs in algae occurs in the ER, after which they can also be transported to plastids [45]. It is known that, due to the substrate-specificity of lysophosphatidic acid acyltransferases, plastidic DAGs contain C16 FAs in the sn-2 position, while ER-derived DAGs contain C18 FAs. An analysis of the positional distribution of FAs made it possible to establish that *U. pinnatifida* galactolipids are synthesized mainly from ER-derived DAG (Table S4). Up to 86.0% SQDG and up to 54.4% PG were synthesized from plastidic DAG molecules. Plastid-derived forms of extraplastidic lipids have been identified, suggesting that DAG may be transported from plastids to synthesize these lipids. Based on the data from the literature [46,47,48], as well as data obtained as a result of the analysis of the FA positional distribution in the molecules of different lipid classes of four species of brown algae (Table S3, [36]) from two orders (Laminariales, Ectocarpales), a scheme of lipid molecular species biosynthesis pathways was constructed (Figure 11).

**Figure 12:**
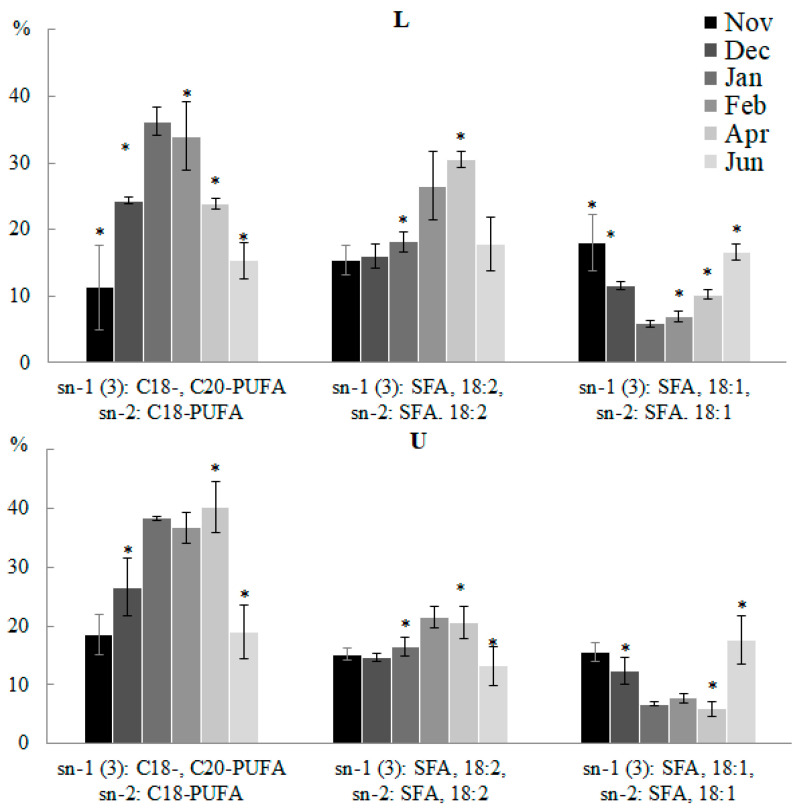
The contents of TAG molecular species (% of the total TAG molecular species) in the lower (L) and upper (U) parts of the blades of *U. pinnatifida* samples collected in different months. An asterisk indicates significant differences from the subsequent data point. An asterisk above the last data point indicates a significant difference between June and February (HSD-test, *p* < 0.05).

Depending on the month of collection and the part of the blade, 60.1% to 81.0% of TAGs were synthesized from ER-derived DAG molecules. ER-derived molecular species of TAGs can be divided into two groups: TAGs synthesized de novo in the Kennedy pathway containing predominantly 16:0, 18:0 and 18:1, and TAGs containing 18:2, 18:3, 18: 4, 20:4, and 20:5, which indicates the formation of this group from DAG fragments containing a pool of FAs that underwent elongation and desaturation in PC. In winter, in all parts of the algal blade, the accumulation of the most unsaturated ER-derived molecular species of TAG was observed, containing mainly C18-PUFA and 20:5 (18:3/18:2/20:5, 18:4/18:3/20: 5, 20:4/18:4/20:5, 20:5/18:2/20:5, 20:5/18:4/20:5, etc.) (Figure 12). In spring, the contents of plastid- and ER-derived molecular species with SFA and 18:2 (16:0/18:2/16:0, 18:1/16:0/18:2, 18:2/16:0/18:2, etc.) increased; their contents then decreased by the summer. In autumn and summer, the proportions of plastid- and ER-derived molecular species of TAG with SFA and MUFA (18:1/16:0/18:1, 14:0/18:1/16:0, 16:0/18:1 /16:0, 16:0/18:1/18:0, etc.) increased. The trend of changes in the contents of various molecular species of TAG correlates with changes in the contents of polar lipid molecular species containing similar FAs. An analysis of the positional distribution of FAs in *U. pinnatifida* lipids collected at different months confirmed an insignificant degree of inclusion of the TAG formation pathway directly from other polar lipids. Thus, seasonal changes in the molecular species composition of TAG reflect the general trends in FA synthesis.

### 2.3. Effect of Infection with the Endophyte Laminariocolax aecidioides on the Lipid Composition of Undaria pinnatifida

The lower (L) and upper (U) tissue sections of the blades of uninfected *U. pinnatifida* samples, and the lower (Li), upper intact (Ui) and upper tissue sections with a visual manifestation of the endophyte (Ue) of infected *U. pinnatifida* samples collected in November (juvenile algae) and June (adult, fertile algae) were used for analysis.

#### 2.3.1. Lipid Classes Composition

**Figure 13:**
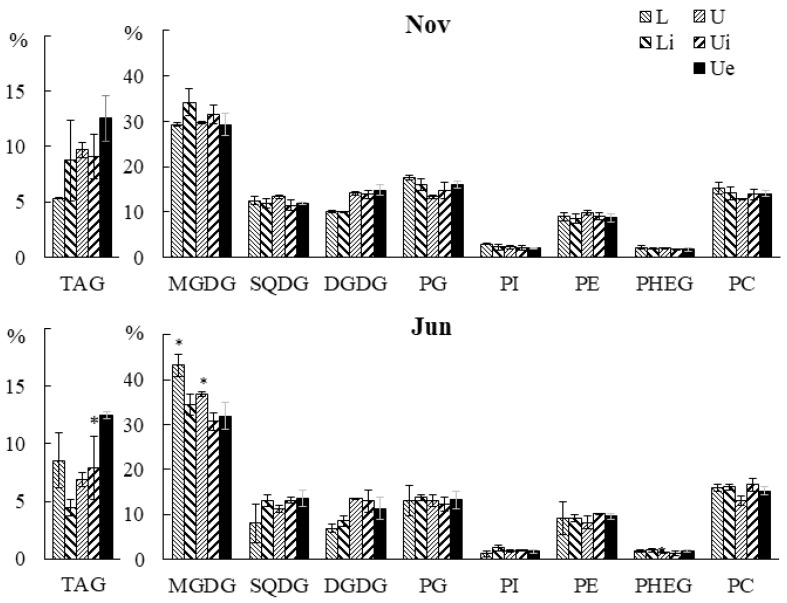
The content of TAG (% of total lipids) and polar lipid classes (% of total polar lipid classes) in the lower (Li), upper intact (Ui), and upper parts of the blades with endophyte (Ue) of infected *U. pinnatifida* samples, and in the lower (L) and upper (U) parts of the blades of uninfected *U. pinnatifida* samples collected in November and June. An asterisk indicates significant differences from the subsequent data point (*t*-test, *p* < 0.05).

Infection had no effect on the contents of major polar lipids in *U. pinnatifida*: MGDG, DGDG, SQDG, PG, PI, PC, PE, and PHEG (Figure 13). A reduced CPI content was observed in the upper part of the blades of juvenile infected algae (both in the tissue with the endophyte (0.4%) and in the intact adjacent tissue (0.4%)) compared with uninfected individuals (0.7%) (Table S1). The CPI content in the lower part of the blades of infected individuals was also lower (0.6% vs. 0.8% in uninfected individuals). It cannot be ruled out that this lipid is a sign of the presence of epi- or endophytic fungi or red algae. The reduced CPI level in infected samples can be explained by the displacement of CPI-containing organisms by proliferating filaments of *L. aecidioides*.

The TAG content varied from 4.5% to 12.5% in different parts of juvenile and adult algae. In adult algae, the TAG content in the infected section tissue was higher compared to the adjacent intact tissue, which may be due to a higher TAG proportion in the endophyte itself or the presence of other endophytes containing a high TAG proportion in the damaged tissue of the host macrophyte.

The DGTS, DGTA, and PG-OH levels were not affected by infection (Table S1).

#### 2.3.2. Fatty Acid Composition

**Figure 14:**
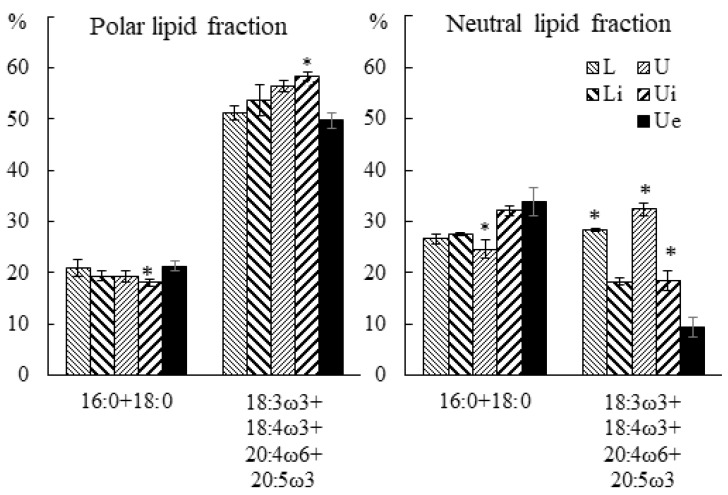
The FA contents of polar and neutral lipid fractions (% of the total FA fraction) in the lower (Li), upper intact (Ui), and upper parts of the blades with endophytes (Ue) of infected *U. pinnatifida* samples, and in the lower (L) and upper (U) parts of the blades of uninfected *U. pinnatifida* samples collected in June. An asterisk indicates significant differences from the subsequent data point (*t*-test, *p* < 0.05).

As a result of the analysis, the same effect of endophyte infection on the FA composition in both juvenile and adult host macrophytes was revealed (Table S2 [13]). In tissues with a pronounced presence of endophyte (Ue), significant changes were observed in the FA composition of both polar and neutral lipids, which were expressed in an increased level of SFAs 16:0 and 18:0, and a reduced level of PUFAs 18:3ω3, 18:4ω3, 20:4ω6 and 20:5ω3 (Figure 14). At the same time, FAs of polar lipids showed such changes only in that part of the blade of the host macrophytes, where the presence of the endophyte was determined visually (Ue), and in neutral lipids, this effect was also manifested in the remaining parts (Li and Ui) of infected samples.

#### 2.3.3. Molecular Species Composition

**Figure 15:**
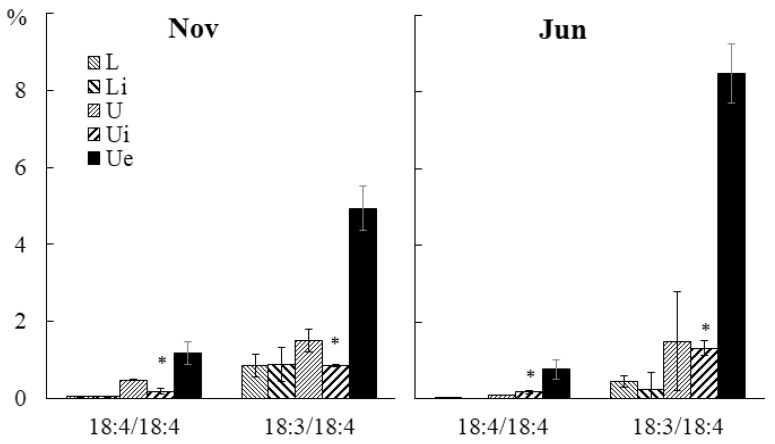
The contents of PG molecular species (% (% of the total PG molecular species) in the lower (Li), upper intact (Ui), and upper parts of the blade with endophyte (Ue) of infected *U. pinnatifida* samples, and in the lower (L) and upper (U) parts of the blades of uninfected *U. pinnatifida* samples collected in November and June. An asterisk indicates significant differences from the subsequent data point (*t*-test, *p* < 0.05).

GL molecular species composition was not significantly affected by infection (Table S3). The content of highly unsaturated PG molecular species such as 18:3/18:4 and 18:4/18:4 in both juvenile and adult algae in the infected section was higher, compared to the intact tissue section (Figure 15).

**Figure 16:**
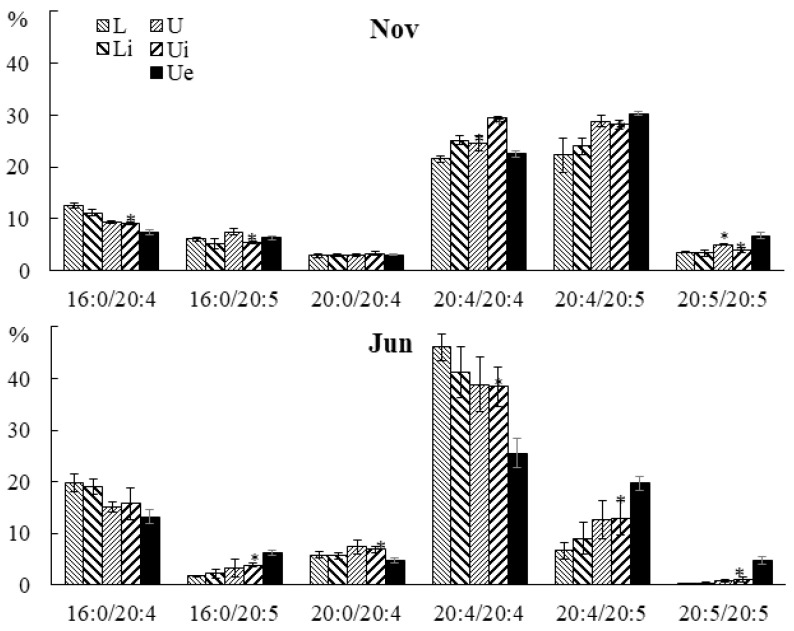
The contents of PE molecular species (% of the total PE molecular species) in the lower (Li), upper intact (Ui), and upper parts of the blades with endophyte (Ue) of infected *U. pinnatifida* samples, and in the lower (L) and upper (U) parts of the blades of uninfected *U. pinnatifida* samples collected in November and June. An asterisk indicates significant differences from the subsequent data point (*t*-test, *p* < 0.05).

The contents of 20:4/20:5 and 20:5/20:5 PE molecular species in juvenile and adult algae in the infected section were higher, and the proportion of 20:4/20:4 was lower, compared with intact tissue (Figure 16). In juvenile algae, the infected sections of blade also had a lower content of 16:0/20:4, and in adult algae, the content was 20:0/20:4.

**Figure 17:**
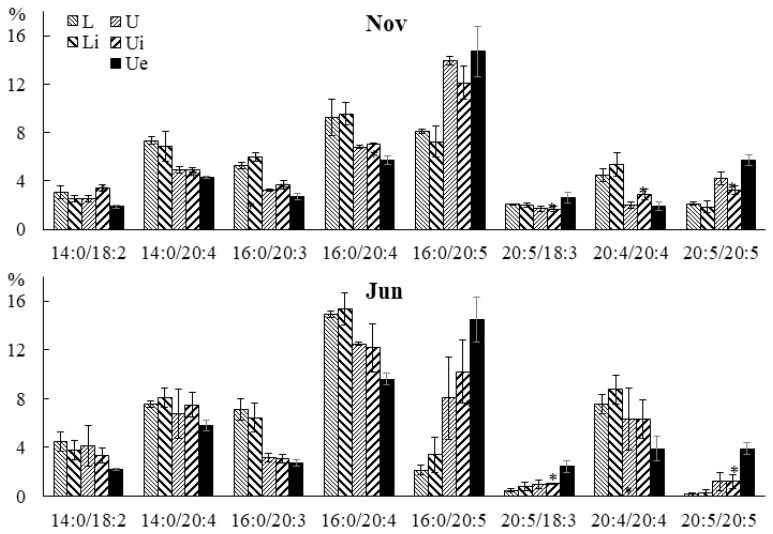
The contents of PC molecular species (% of the total PC molecular species) in the lower (Li), upper intact (Ui), and upper parts of the blades with endophytes (Ue) of infected *U. pinnatifida* samples, and in the lower (L) and upper (U) parts of the blades of uninfected *U. pinnatifida* samples collected in November and June. An asterisk indicates significant differences from the subsequent data point (*t*-test, *p* < 0.05).

A similar trend was observed in PC molecular species composition (Figure 17). Thus, in juvenile and adult algae in the infected sections of the blade, the contents of 20:5/18:3 and 20:5/20:5 PC molecular species were increased. The proportions of 14:0/18:2, 14:0/20:4, 16:0/20:3, and 16:0/20:4 PC molecular species decreased in the infected sections only in juvenile algae.

**Figure 18:**
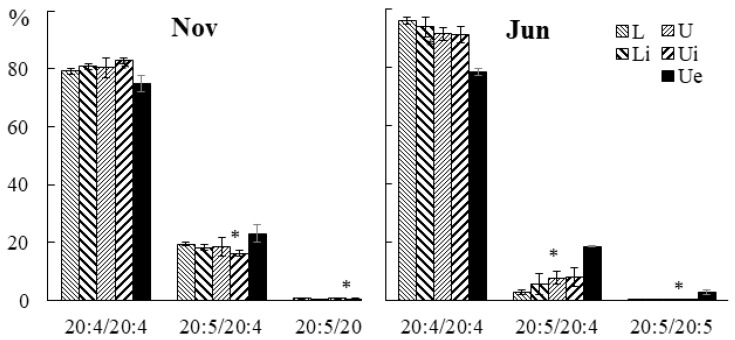
The content of PHEG molecular species (% of the total PHEG molecular species) in the lower (Li), upper intact (Ui), and upper parts of the blade with endophyte (Ue) of infected *U. pinnatifida* samples, and in the lower (L) and upper (U) parts of the blades of uninfected *U. pinnatifida* samples collected in November and June. An asterisk indicates significant differences from the subsequent data point (*t*-test, *p* < 0.05).

The contents of the 20:4/20:4 PHEG molecular species decreased in infected tissue sections in juvenile and adult algae, while the contents of 20:5/20:4 and 20:5/20:5 increased (Figure 18).

An increase in the proportion of PUFA/PUFA molecular species of extraplastidic lipids (PG, PE, PC, and PHEG) in infected sections of both juvenile and adult *U. pinnatifida* individuals may be associated with the mechanical effect of the endophyte on *U. pinnatifida* cells. It has been shown that proliferating filaments of *L. aecidioides* inside macrophyte tissue cause lateral cell contraction [7]. Similar to high hydrostatic pressure, this can cause the restriction of the mobility of lipid molecules [49]. It is possible that an increase in the content of PUFA/PUFA molecular species of structural lipids with extremely unsaturated FAs for them in infected sections compensates for this effect.

**Figure 19:**
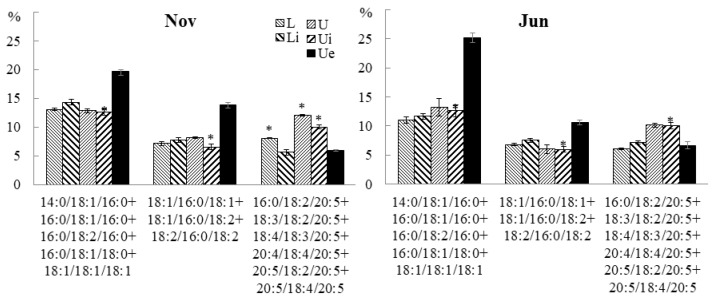
The contents of TAG molecular species (% of the total TAG molecular species) in the lower (Li), upper intact (Ui), and upper parts of the blades with endophytes (Ue) of infected *U. pinnatifida* samples, and in the lower (L) and upper (U) parts of the blades of uninfected *U. pinnatifida* samples collected in November and June. An asterisk indicates significant differences from the subsequent data point (*t*-test, *p* < 0.05).

The infection most noticeably affected the TAG molecular species composition of the *U. pinnatifida*. Thus, the contents of more saturated ER- and plastid-derived TAG species, such as 14:0/18:1/16:0, 16:0/18:1/16:0, 16:0/18:2/16:0, 16:0/18:1/18:0, 18:1/18:1/18:1, 18:1/16:0/18:1, 18:1/16:0/18:2, and 18:2/16:0/18:2, increased, while the proportion of ER-derived highly unsaturated TAG molecular species, such as 16:0/18:2/20:5, 18:3/18:2/20:5, 18:4/18:3/20:5, 20:4/18:4/20:5, 20:5/18:2/20:5, and 20:5/18:4/20:5, decreased (Figure 19). In juvenile individuals, a decrease in the content of TAG molecular species with PUFA was observed in all parts of the blade.

The presence of an endophyte violates the integrity of the outer integument, which opens access for secondary infections [50]. The accumulation of TAG molecular species containing SFAs and monounsaturated FAs (MUFAs) with 16 and 18 carbon atoms in the infected and adjacent parts of the blade may be associated with the penetration of a secondary infection; for example, viral or fungal. Previously, it was found that when unicellular algae are infected with a virus, an increase in the synthesis of TAGs with SFAs is observed, with their accumulation in the form of lipid droplets [51]. During the joint cultivation of microalgae with filamentous fungi, the accumulation of TAGs containing FAs such as 16:0, 18:0, 18:1, and 18:2 was also observed [52]. The decrease in the proportion of TAG molecular species with PUFAs in the infected sections of juvenile algae may be associated with a lower content of these TAG species in the endophyte itself.

## 3. Materials and Methods

### 3.1. Algae Material

**Figure 20:**
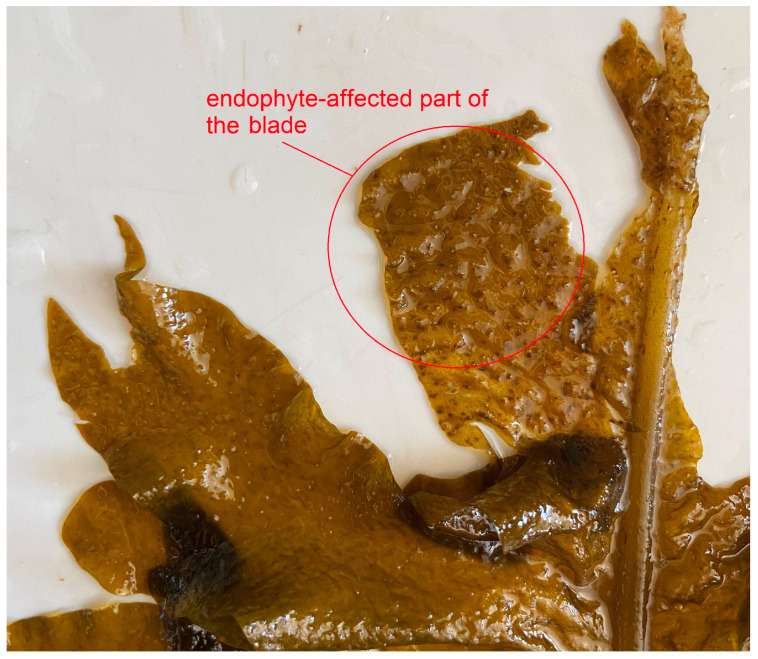
Upper part of the *Undaria pinnatifida* sample infected with endophyte *Laminariocolax aecidioides*.

*Undaria pinnatifida* samples were collected by diving from natural habitats at a depth of 1–2 m (Table 2). The algae were placed in a bucket of seawater, immediately transported to the laboratory, and thoroughly cleaned. The upper parts of the blades of the samples were examined for the presence of infection with the endophytic brown alga *Laminariocolax aecidioides* (dark brown spots of the *U. pinnatifida* blade surface) (Figure 20). From uninfected samples, tissue sections (0.2–1 g) of the upper (U) and lower (L) parts of the blade were taken; from infected samples, the lower parts of the blade (Li), intact tissue sections of the upper parts of the blade (Ui) and well-distinguished affected pigmented tissue sections of the upper parts of the blade (Ue) were taken. Pieces of tissue were quickly dried using filter paper, weighed, and placed in glass test tubes with 5 mL of a mixture of chloroform/methanol (1:1, *v*/*v*.) for the subsequent extraction of lipids.

### 3.2. Lipid Extraction

Algae samples were crushed using a homogenizer Ultra-Turrax T 25 (IKA, Germany) with an S25N-10G dispersing element. Tissue in a glass tube was homogenized for 2 min at 10,000 rpm with 5 mL of chloroform/methanol (1:1, *v*/*v*). The organic phase was collected in pear-shaped flasks using filter paper. The biomass was re-extracted six times. The final organic phase was dried using a rotary evaporator, transferred to glass vials, dried, weighed and stored in chloroform at −20 °C.

### 3.3. Fatty Acids Analysis by GC and GC-MS

The analysis of FA methyl esters was carried out using a Shimadzu GC-2010 (Kyoto, Japan) gas chromatograph with a flame ionization detector equipped with a Supelcowax-10 column (30 m × 0.25 mm × 0.25 µm), (Supelco, Bellefonte, PA, USA). The temperatures of the column, injector and detector were 200 °C, 250 °C, and 240 °C, respectively. FAMEs were identified based on the calculation of the equivalent chain length (ECL) [53] and comparison with known standards.

If structure confirmation was required, FAMEs were analyzed by GC-MS Shimadzu GCMS-2010 (Kyoto, Japan) with a SH-Rtx-5MS column (30 m × 0.25 mm × 0.25 μm), (Restek Corporation, Bellefonte, PA, USA). The temperature gradient of the column used was 160 °C (2 min)–2 °C/min–260 °C (20 min), while the injector and interface temperatures were 250 and 240 °C, respectively.

### 3.4. HPLC-MS/MS Analysis of Molecular Species

Each sample was analyzed twice by HPLC-MS, once in HILIC mode to determine the composition of lipid classes, and a second time on the reversed phase column to determine the composition of the molecular species of each class. The analysis of the composition of lipid classes using HILIC chromatography allows us to get more accurate and reproducible results, especially for minor compounds.

Quantitative analyses of molecular species and their identification using fragmentation patterns were performed on a Shimadzu HPLC system (Kyoto, Japan) (degassing units DGU-20A3r and DGU-20A5r, four pumps LC-30AD, autosampler SIL-30AC, column oven CTO-20AC and controller CBM-20A) connected to a Shimadzu LCMS-8060 triple-quadrupole mass spectrometer (Kyoto, Japan) with an electrospray ionization ion source. The column Ascentis Express 90 Å C18 (150 × 2.1 mm i.d., 2 µm) (Supelco, Bellefonte, PA, USA) was operated at 50 °C. The following mobile phases were used for the quaternary high-pressure gradient: A, methanol; B, 2-propanol; C, water containing 2 M formic acid and 1.8 M ammonia; D, water. A, B and D eluent channels were connected to a mixer (40 µL volume) through a cartridge (10 mm × 2 mm ID) with SCX-1001 cationite (Yanaco, Japan) and C channel was connected directly to a mixer. Eluent was pumped at a constant flow of 0.2 mL/min with the following gradient (A:B:C:D, % by vol.): 0 min (26.25:48.75:2.5:22.5), 5 min (18.75:56.25:2.5:22.5), 15 min (15:60:2.5:22.5:), 20 min (7.5:67.5:2.5:22.5), 22 min (0:75:2.5:22.5), 30–35 min (0:85:2.5:12.5), 45 min (0:87.5:2.5:10), 55 min (0:97.5:2.5:0), 57 min (0:100:0:0), 65 min (0:100:0:0), 65.01–74 min (26.25:48.75:2.5:22.5). The elution range of polar lipids was from 10 to 30 min, while that of triglycerides was from 35 to 60 min; 0.2–0.5 µL of total lipid extract in chloroform with 1 mg/mL lipid concentration was injected.

The MS parameters were as follows: the temperatures of the interface, heat block, and desolvatation line were 300, 400, and 250 °C, respectively; the flow rates of drying gas (N_2_), nebulizer gas (N_2_) and heating gas (zero air) were 10 L/min, 3 L/min and 10 L/min, respectively. Negative ion mode was applied for the detection of PI and CPI, and the positive mode was applied for other lipid classes. Previously described fragmentation reactions were used for the detection and determination sn-positions of acyl chains in all polar lipid classes [35] (except sn-positions of GlcADG and PHEG molecular species). For the determination of acyl sn-positions in MGDG, DGDG and PC molecular species, [M+Li]^+^ ions were used; in this case 5, mM LiOH in methanol with 0.02 mL/min flow was added postcolumn. [M + Li]^+^ ions have also been used to separate DGTS and DGTA molecular species according to the fragmentation reactions described by Li et al., 2017 [54]. TAGs were analyzed in the form of [M+NH_4_]^+^ ions; the sn-positions of acyl groups were determined by fragmentation at 28 eV collision energy [55,56,57,58].

### 3.5. HPLC-MS/MS Analysis of Lipid Classes

Quantitative analysis of lipid classes was performed using HPLC-MS. An Ascentis Si column (250 × 2.1 mm i.d., 5 µm) (Supelco, USA) in HILIC mode at 40 °C was used. A binary high-pressure gradient was used (B% by vol., total flow, mL/min): 0 min (2%, 0.2), 2.5 min (24%, 0.2), 2.99 min (0.2), 3 min (0.15), 5 min (26%, 0.15), 12 min (40%), 16–19.99 min (100%, 0.15), 20–21.49 min (100%, 0, 3), 21.5–24.99 min (2%, 0.3), 25–26 min (2%, 0.2), mixer volume—40 µL. Eluent A was acetonitrile containing 50 mM formic acid, eluent B was acetonitrile:water (1:1, *v*/*v*) containing 100 mM formic acid and 40 mM ammonium hydroxide; 0.1 μL of lipid extract with a concentration of 1 mg/mL was injected. For the detection of lipid classes, the same mass spectrometer parameters were used as for the molecular species analysis. Individual lipid classes isolated from *U. pinnatifida* extracts were used as standards for external calibration. For triglyceride detection, a total ion current in the range of 700–868 + 880–1100 m/s was used, since pigments interfered in the range of 868–880 *m*/*z*.

### 3.6. Statistical Analysis

All statistical analyses were performed using Microsoft Excel (Microsoft, Redmond, WA, USA). All values were presented as mean ± standard deviation for triplicate. The data were assessed statistically by one-way ANOVA and Tukey HSD test for a posteriori comparison, as well as by Student’s *t*-test. A probability level of *p* < 0.05 was considered significant.

## 4. Conclusions

In this work, the complete lipidome of the brown alga *U. pinnatifida* was characterized. Based on the data obtained, the biosynthesis pathways of various lipid molecular species in brown algae were determined.

The main molecular species of structural lipids involved in the seasonal adaptation of *U. pinnatifida* have been established. It was shown that acclimatization to winter and spring is accompanied by an increase in the most unsaturated molecular species of plastidic lipids—MGDG and DGDG containing 18:4 and 20:5, PG containing C18-PUFA, as well as extraplastidic lipids PE, PC, and PHEG containing 20:5. In autumn and summer, the contents of less unsaturated MGDG and DGDG molecular species with 18:3 and 20:4, as well as PG and SQDG with SFAs and PE, PC, and PHEG containing 20:4, were increased. During the year, changes were also observed in the compositions of storage lipids in alga. In winter, ER-derived molecular species of TAG with PUFAs accumulated, while in spring, plastid- and ER-derived molecular species accumulated, representing combinations of SFAs, MUFAs, and PUFAs, and in autumn and summer, plastid- and ER-derived molecular species with SFAs and MUFAs did.

Under natural conditions, *U. pinnatifida* is often infected with the brown filamentous endophyte *Laminariocolax aecidioides*. We studied the influence of endophytic infection on the *U. pinnatifida* lipidome. In infected sections of the algal blade, the proportion of structural lipids, such as PG, PE, PC, and PHEG, containing PUFA was increased, which may be a reaction in response to mechanical stress caused by the growth of endophytic filaments inside the host macrophyte tissue. In infected, as well as adjacent intact tissue sections of the *U. pinnatifida* blade in the contents of TAG molecular species containing SFAs and MUFAs were increased, which may indicate the occurrence of a secondary infection.

## Figures and Tables

**Table 1:** Lipid molecular species composition of *Undaria pinnatifida*.

Lipid Class	Total Number of Molecular Species	Main Molecular Species
Polar lipids		
Glycoglycerolipids		
MGDG	146	18:4/18:4, 20:5/18:4, 18:3/18:4, 20:4/18:4
DGDG	66	20:5/18:4, 20:4/18:4, 16:0/18:3, 14:0/18:3, 14:0/18:3, 18:3/18:4, 20:5/18:3
SQDG	47	18:1/16:0, 18:2/16:0, 18:3/16:0+16:0/18:3, 14:0/18:2, 14:0/16:0, 16:0/16:0
GlcADG	36	14:0/18:2, 14:0/18:3, 14:0/18:1, 16:1/18:1, 16:0/18:3, 16:0/18:1, 18:1/18:2, 18:1/18:1
Phosphoglycerolipids		
PG	105	18:3/16:0, 16:0/18:2, 16:0/18:1, 18:3/18:3
PE	96	20:4/20:4, 20:4/20:5, 16:0/20:4, 16:0/20:5, 20:5/20:5
PC	82	16:0/20:5, 16:0/20:4, 16:0/18:2, 16:0/18:3, 14:0/20:4, 14:0/20:5, 20:4/20:4, 20:4/20:5
PI	21	16:0/18:1, 16:0/18:2, 16:0/18:0, 16:0/18:3
PHEG	5	20:4/20:4, 20:5/20:4
Betaine lipids		
DGTA	38	18:4/14:0, 20:5/14:0, 18:4/20:5, 20:5/20:5, 18:2/14:0, 16:1/14:0
DGTS	47	18:4/16:0, 18:3/16:0, 16:1/14:0, 18:4/14:0, 18:2/14:0, 16:1/16:0, 18:3/18:1, 18:1/18:1
Phosphosphingolipids		
CPI	28	22:1d/14:0, 22:0d/14:0, 22:1d/13:0, 22:0d/16:0, 22:1d/16:0
Neutral lipids		
TAG	190	14:0/18:1/16:0, 16:0/18:1/16:0, 16:0/18:1/18:0, 18:1/16:0/18:1, 16:0/18:2/20:5, 18:4/18:3/20:5, 20:4/18:4/20:5, 20:5/18:4/20:5

MGDG, monogalactosyldiacylglycerol; DGDG, digalactosyldiacylglycerol; SQDG, sulfoquinovosyldiacylglycerol; GlcADG, glucuronosyldiacylglycerol; PG, phosphatidylglycerol; PE, phosphatidylethanolamine; PC, phosphatidylcholine; PI, phosphatidylinositol; PHEG, phosphatidylhydroxyethylglycine; DGTA, diacylglycerylhydroxymethyl-N,N,N-trimethyl-β-alanine; DGTS, diacylglyceryl-N,N,N-trimethyl-homoserine; CPI, ceramide phosphoinositol; TAG, triacylglyceride. Numbers C:db indicates the number of carbon atoms (C) and double bonds (db) in the fatty acid chains. Molecular species whose contents were higher than 5% of the total molecular species of a given class in at least one of the samples in descending order are shown (except TAG).

**Table 2:** Date and site of collection of *Undaria pinnatifida* samples.

Month	Site of Collection	Water Temperature
November	Sobol Bay (Ussuriisky Bay, Peter the Great Bay, Sea of Japan)	5 °C
December	Lazurnaya Bay (Ussuriisky Bay, Peter the Great Bay, Sea of Japan)	0.5 °C
January	Lazurnaya Bay (Ussuriisky Bay, Peter the Great Bay, Sea of Japan)	0–1 °C
February	Lazurnaya Bay (Ussuriisky Bay, Peter the Great Bay, Sea of Japan)	0–1 °C
April	Cape Zeleny (Amur Bay, Peter the Great Bay, Sea of Japan)	0 °C
June	Cape Zeleny (Amur Bay, Peter the Great Bay, Sea of Japan)	18 °C

## Data Availability

Not applicable.

## Supplementary Materials

The following supporting information can be downloaded at https://www.mdpi.com/article/10.3390/md21090466/s1, Table S1: The polar lipid classes contents (% of the total polar lipids) and TAG (% of the total lipids) of various parts of the blades of *Undaria pinnatifida* samples uninfected and infected with the endophyte *Laminariocolax aecidioides* collected in different months. Table S2: The fatty acids composition of various parts of the blades of *Undaria pinnatifida* samples uninfected (L and U) and infected with the endophyte *Laminariocolax aecidioides* (Li, Ui and Ue) collected in June. Table S3: Molecular species composition of MGDG, DGDG, SQDG, PG, PE, PC, PI, PHEG, and TAG of various parts of the blades of *Undaria pinnatifida* samples uninfected and infected with the endophyte *Laminariocolax aecidioides* collected in different months. Table S4: The plastid-derived lipid molecular species of MGDG, DGDG, SQDG, PG, PE, PC, PI, and TAG of various parts of the blades of *Undaria pinnatifida* samples uninfected and infected with the endophyte *Laminariocolax aecidioides* collected in different months.

## Abbreviations

BL	Betaine lipid
DAG	Diacylglyceride
DGDG	Digalactosyldiacylglycerol
DGTS	Diacylglyceryl-N,N,N-trimethylhomoserine
ER	Endoplasmic reticulum
FA	Fatty acid
GL	Glycoglycerolipid
GlcADG	Glucuronosyldiacylglycerol
LHC II	Light-harvesting complex II
MGDG	Monogalactosyldiacylglycerol
MUFA	Monounsaturated fatty acid
PC	Phosphatidylcholine
PE	Phosphatidylethanolamine
PG	Phosphatidylglycerol
PHEG	Phosphatidylhydroxyethylglycine
PI	Phosphatidylinositol
PL	Phosphoglycerolipid
PSII	Photosystem II
PUFA	Polyunsaturated fatty acid
SFA	Saturated fatty acid
SQDG	Sulfoquinovosyldiacylglycerol
TAG	Triacylglyceride
