# Supplementary material for: Lipidome of the Brown Macroalga Undaria pinnatifida: Influence of Season and Endophytic Infection
Source: Mar Drugs. 2023 Aug 25;21(9):466. doi: 10.3390/md21090466 (PMC10532667; doi:10.3390/md21090466)
Supplement: Supplementary file 1 [file marinedrugs-21-00466-s001.zip › marinedrugs-2558783-supplementary.pdf]

Supplementary data content page

Title: Lipidome of the Brown Macroalga *Undaria pinnatifida*: Influence of Season and Endophytic Infection

Authors: Ksenia Chadova and Peter Velansky

Address: A.V. Zhirmunsky National Scientific Center of Marine Biology, Far Eastern Branch of Russian Academy of Sciences, Vladivostok 690041. Russia

Correspondence: chadova_9595@mail.ru

Table S1. The polar lipid classes content (% of the total polar lipids) and TAG (% of the total lipids) of various parts of the blades of *Undaria pinnatifida* samples uninfected and infected with the endophyte *Laminariocolax aecidioides* collected in different months. Values given as mean ± s.d. for triplicate. "+" – amount less than 0.1%, "-" – not detected. Between columns result of Tukey test and Students t-test (p<0.05, n=3) are shown (">", "<" – statistically significant difference, "=" – no significant changes). "T" – difference between «June» and «February», S» – difference between uninfected and infected samples.

Lipid	The lower part of the blades of infected *U. pinnatifida* samples											
	Nov.		Dec.		Jan.		Feb.		Apr.		Jun.	T
TAG	8.7±3.7	=	6.9±2.0	=	5.9±1.4	<	14.7±3.9	=	12.5±1.5	>	4.5±0.7	<
MGDG	34.1±3.0	=	35.1±2.7	>	25.4±1.1	=	24.7±2.6	>	21.1±1.3	<	34.4±2.2	>
GlcADG	0.4±0.1	=	0.5±0.0	>	0.4±0.1	<	0.6±0.1	=	0.6±0.1	>	0.2±0.1	<
SQDG	11.9±1.0	>	3.9±1.7	<	6.2±0.3	<	10.3±0.9	=	11.4±0.8	=	12.9±1.3	>
DGDG	9.9±0.0	<	18.7±2.0	>	13.9±0.9	=	12.4±2.0	=	13.0±1.0	>	8.5±1.3	<
PG	16.0±1.2	<	19.1±0.5	>	17.2±0.6	>	14.8±0.9	=	15.6±1.3	>	13.7±0.7	=
PG-OH	0.3±0.0	<	0.5±0.0	=	0.5±0.1	=	0.5±0.1	>	0.3±0.0	>	0.1±0.0	<
PI	2.2±0.6	>	1.0±0.3	<	5.4±0.3	>	4.7±0.2	>	2.9±0.3	=	2.5±0.7	<
CPI	0.6±0.1	>	0.1±0.0	<	0.5±0.3	=	0.4±0.1	>	0.2±0.1	<	0.5±0.2	=
PE	8.5±1.0	=	8.5±0.9	<	10.9±1.0	<	13.7±1.3	<	15.3±1.0	>	9.1±0.8	<
DGTS	+	=	+	=	+	=	+	=	+	=	+	=
DGTA	+	=	+	=	-	=	0.1±0.0	=	-	=	+	=
PHEG	1.9±0.1	>	1.1±0.1	<	1.7±0.1	<	2.1±0.3	=	2.3±0.2	=	1.9±0.4	=
PC	14.2±1.4	>	11.3±1.4	<	18.0±0.6	=	16.0±1.8	=	17.2±0.8	>	16.0±0.6	=

Lipid	The upper intact part of the blades of infected *U. pinnatifida* samples											
	Nov.		Dec.		Jan.		Feb.		Apr.		Jun.	T
TAG	9.0±2.0	=	8.5±0.7	=	9.3±0.7	<	19.7±0.9	=	17.2±2.2	>	7.9±2.8	<
MGDG	31.6±2.1	<	45.4±2.6	>	27.7±0.8	=	26.1±1.5	=	24.5±0.9	<	30.7±2.0	>
GlcADG	0.8±0.1	=	0.6±0.0	=	0.5±0.1	=	0.5±0.0	=	0.5±0.1	=	0.6±0.3	=
SQDG	11.5±1.1	>	1.0±0.3	<	5.9±0.1	<	9.1±0.5	=	9.0±1.0	<	13.1±0.7	>
DGDG	13.9±0.9	>	7.2±2.5	<	20.9±0.6	=	22.5±2.0	=	23.0±3.2	>	12.9±2.5	<
PG	14.8±1.8	=	12.3±2.9	<	19.4±1.3	>	13.7±1.2	=	14.6±1.1	>	12.2±1.6	=
PG-OH	0.3±0.0	>	0.2±0.1	<	0.7±0.1	>	0.4±0.1	=	0.4±0.1	>	0.1±0.1	<
PI	2.0±0.5	>	0.5±0.3	<	3.1±0.8	=	3.2±0.8	=	2.4±0.9	>	2.0±0.1	<
CPI	0.4±0.1	>	0.1±0.0	<	0.3±0.1	=	0.3±0.0	>	0.2±0.1	<	0.5±0.1	>
PE	9.0±0.8	<	15.4±0.3	>	7.7±0.4	<	9.2±0.6	<	11.0±1.3	=	10.0±0.2	>
DGTS	+	=	+	>	+	=	+	=	+	=	+	>
DGTA	0.1±0.1	=	-	=	-	=	0.1±0.1	=	-	=	+	=
PHEG	1.7±0.1	=	1.7±0.4	>	1.2±0.1	<	1.5±0.1	=	1.4±0.2	=	1.3±0.6	=
PC	13.9±1.2	=	15.5±1.4	>	12.7±1.4	=	13.5±0.4	=	12.9±2.2	<	16.6±1.5	>

Lipid	The upper part of the blades of infected *U. pinnatifida* samples with endophyte		The lower part of the blades of uninfected *U. pinnatifida* samples				The upper part of the blades of uninfected *U. pinnatifida* samples			
	Nov.	Jun.	Nov.	S	Jun.	S	Nov.	S	Jun.	S
TAG	12.5±2.1	12.5±0.3	5.3±0.1	=	8.6±2.4	=	9.7±0.7	=	6.9±0.6	=
MGDG	29.2±2.5	31.9±3.0	29.2±0.4	=	43.2±2.4	>	29.7±0.3	=	36.7±0.6	>
GlcADG	1.0±0.1	0.9±0.2	0.5±0.0	=	0.2±0.0	=	1.0±0.2	=	0.3±0.0	=
SQDG	11.8±0.3	13.5±1.8	12.5±0.9	=	8.0±4.3	=	13.3±0.3	=	11.2±0.8	=
DGDG	14.8±1.2	11.3±2.5	10.0±0.2	=	6.8±0.9	=	14.1±0.4	=	13.6±0.1	=
PG	16.0±0.7	13.2±1.9	17.5±0.5	=	13.1±3.4	=	13.3±0.4	=	13.0±1.3	=
PG-OH	0.2±0.1	0.1±0.1	0.3±0.0	=	0.1±0.0	=	0.3±0.1	=	0.2±0.1	=
PI	2.0±0.2	1.7±0.4	2.9±0.2	=	1.2±0.6	=	2.3±0.4	=	1.8±0.3	=
CPI	0.4±0.1	0.5±0.2	0.8±0.1	>	0.3±0.1	=	0.7±0.0	>	0.5±0.2	=
PE	8.7±0.9	9.5±0.6	9.0±0.9	=	9.1±3.7	=	9.8±0.6	=	8.2±1.4	=
DGTS	+	0.1±0.0	+	=	+	=	+	>	+	=
DGTA	0.1±0.1	+	+	=	-	=	0.6±0.0	=	+	=
PHEG	1.7±0.3	1.8±0.2	2.1±0.3	=	1.8±0.3	=	1.9±0.1	=	1.4±0.2	=
PC	14.1±0.7	15.2±0.9	15.2±1.2	=	16.0±0.8	=	12.8±0.0	=	13.0±1.0	<

Table S2. The fatty acids composition of various parts of the blades of *Undaria pinnatifida* samples uninfected (L and U) and infected with the endophyte *Laminariocolax aecidioides* (Li, Ui and Ue) collected in June. Values given as % of total fatty acids of the fraction (mean ± s.d. for triplicate). "+" – amount less than 0.1%, "-" – not detected. Between columns result of Students t-test (p<0.05, n=3) are shown (">", "<" – statistically significant difference, "=" – no significant changes).

FA	Polar lipids fraction								Neutral lipids fraction							
	L		Li	U		Ui		Ue	L		Li	U		Ui		Ue
12:0	0.3±0.0	>	+	0.1±0.0	=	0.1±0.1	=	0.2±0.1	0.9±0.9	=	1.8±0.5	1.3±0.3	=	1.5±0.5	=	1.6±0.0
14:0	3.6±0.4	=	3.9±0.2	3.1±0.1	=	3.8±0.9	=	4.5±0.5	7.2±0.3	<	10.2±0.8	8.8±1.6	=	9.4±1.1	>	7.3±0.3
14:1ω-7	0.4±0.0	>	+	0.2±0.0	=	0.2±0.2	=	0.2±0.0	2.2±0.4	>	-	0.4±0.1	>	-	<	+

14:1ω-5	-	<	+	-	<	+	=	+	-	=	-	-	=	-	=	-
i-15:0	0.1±0.0	=	+	+	=	+	<	0.2±0.1	0.6±0.1	=	0.5±0.0	0.5±0.2	=	0.5±0.2	=	1.4±0.9
ai-15:0	0.2±0.0	<	+	0.1±0.0	=	0.1±0.0	=	0.1±0.0	0.9±0.1	=	1.0±0.1	0.8±0.5	=	1.1±0.9	=	1.5±0.0
15:0	0.6±0.3	=	0.2±0.0	0.4±0.1	=	0.5±0.3	=	0.6±0.1	+	<	2.3±0.9	+	<	1.7±0.5	>	+
15:1ω-7	+	=	+	+	=	+	=	0.1±0.0	0.6±0.0	>	0.3±0.0	-	<	0.7±0.2	=	0.5±0.0
i-16:0	0.3±0.0	>	+	0.2±0.0	=	0.7±0.6	=	0.2±0.0	-	=	-	-	=	-	<	0.6±0.0
16:0	19.3±3.1	=	17.8±1.9	18.1±2.0	=	16.3±1.0	<	19.3±1.4	22.0±1.2	=	23.1±0.5	20.5±3.1	<	26.3±2.0	<	30.2±4.3
16:1ω-9	1.4±0.0	>	0.1±0.0	0.8±0.0	=	0.6±0.5	=	0.8±0.1	0.2±0.3	=	1.2±0.1	1.0±0.2	=	0.8±0.0	=	1.4±0.7
16:1ω-7	0.5±0.1	=	0.3±0.0	0.4±0.1	=	0.5±0.0	=	0.6±0.2	1.2±0.3	=	1.0±0.1	1.0±0.1	=	1.3±0.6	=	2.7±0.8
16:1ω-5	+	<	0.1±0.0	-	<	0.1±0.1	=	0.1±0.0	-	<	+	-	=	-	<	0.2±0.2
trans-16:1Δ3	0.4±0.2	=	1.0±0.3	0.4±0.1	<	1.5±0.5	=	1.9±0.2	0.1±0.1	=	0.3±0.0	0.2±0.1	=	0.3±0.3	=	1.5±0.9
ai-17:0	0.1±0.0	=	+	0.1±0.0	=	+	=	0.1±0.0	0.3±0.2	=	1.9±0.8	0.2±0.2	<	1.4±0.0	=	1.7±1.5
16:2ω-4	-	=	-	+	=	+	=	+	0.7±0.5	=	+	0.6±0.5	=	0.1±0.0	<	0.3±0.0
17:0	0.2±0.0	=	0.1±0.0	0.1±0.0	=	0.2±0.1	=	0.2±0.0	0.8±0.1	=	0.9±0.1	0.7±0.1	=	1.1±0.4	=	0.6±0.1
17:1ω-9	0.2±0.0	>	+	0.1±0.0	=	0.1±0.0	=	0.2±0.0	1.5±0.1	=	1.2±0.2	1.2±0.3	=	1.6±1.2	=	0.6±0.0
16:3ω-3	0.1±0.0	>	-	+	=	0.1±0.0	=	0.1±0.0	0.3±0.4	=	0.9±0.0	0.3±0.3	=	0.1±0.0	>	-
17:1ω-5	0.1±0.0	=	+	0.1±0.0	=	0.1±0.0	=	0.1±0.1	-	>	1.3±0.0	-	<	0.6±0.7	>	-
i-18:0	0.2±0.0	>	+	0.1±0.0	=	0.1±0.0	=	0.1±0.0	1.1±0.2	=	1.3±0.1	0.6±0.0	=	1.9±1.1	=	2.8±0.0
17:2ω-6	+	>	-	+	=	+	=	+	0.4±0.2	=	0.3±0.1	0.4±0.0	=	0.3±0.1	=	1.4±1.3
16:4ω-1	-	<	+	-	<	+	=	+	-	<	0.1±0.0	-	<	0.1±0.0	>	-
18:0	1.6±0.2	=	1.5±0.1	1.2±0.1	<	1.8±0.2	=	2.0±0.4	4.6±0.6	=	4.4±0.1	4.1±0.4	<	5.8±0.2	>	3.7±1.4
18:1ω-9	8.0±1.5	=	9.2±1.2	7.7±0.3	>	5.3±0.6	=	6.7±0.8	10.6±0.7	=	14.6±0.2	9.8±1.9	<	14.0±0.3	<	15.4±1.0
18:1ω-7	0.1±0.0	>	+	0.1±0.1	=	0.2±0.1	=	0.3±0.1	0.3±0.2	=	0.6±0.3	0.2±0.0	=	0.5±0.2	=	2.6±2.3
18:1ω-6/ 18:1ω-5	-	<	+	-	=	-	=	-	-	=	-	-	=	-	=	-
18:2ω-6	7.0±0.3	=	7.2±0.6	6.9±0.0	=	6.5±0.8	=	6.4±0.6	7.4±0.5	=	7.9±0.6	7.9±1.1	=	6.5±0.8	=	5.6±0.4
19:0	+	>	-	-	<	+	=	+	0.1±0.0	=	0.1±0.0	0.1±0.0	=	0.1±0.1	<	0.5±0.0
18:3ω-6	1.3±0.4	=	2.0±0.4	1.2±0.1	<	0.6±0.3	=	0.6±0.1	1.8±0.1	=	1.3±0.3	1.8±0.1	>	0.5±0.0	=	0.5±0.0
Σ 19:1	-	=	-	-	=	-	=	-	-	=	-	-	=	-	=	-
18:3ω-3	10.4±1.7	=	8.7±1.8	11.2±0.5	=	10.9±0.4	=	9.6±1.4	5.6±0.4	>	2.7±0.2	6.7±0.9	>	4.1±0.9	>	1.6±1.4
i-20:0	-	<	+	-	=	0.1±0.0	=	0.1±0.0	-	=	-	-	=	-	<	0.1±0.0
18:4ω-3	16.5±0.2	=	18.0±5.1	20.2±1.2	<	14.2±1.8	=	10.6±1.2	4.7±0.3	=	3.5±1.2	5.3±0.7	=	4.4±2.8	=	3.2±3.5
18:4ω-1	-	<	+	-	<	+	=	+	-	=	-	-	=	-	=	-
18:5ω-3	-	<	+	-	=	+	=	+	-	=	-	+	>	-	=	-
20:0	0.4±0.1	=	0.5±0.1	0.4±0.1	=	0.9±0.4	=	0.9±0.1	0.7±0.0	=	0.8±0.1	0.7±0.1	=	0.9±0.3	=	1.5±1.4
20:1ω-9	-	<	+	-	<	+	<	0.1±0.0	-	<	0.3±0.0	-	<	0.5±0.0	>	0.2±0.0
20:2ω-6	0.1±0.1	=	0.1±0.0	0.1±0.0	=	0.1±0.1	=	0.1±0.0	1.4±1.2	=	0.6±0.4	1.5±1.2	=	0.4±0.3	=	-
20:3ω-9	-	=	-	-	<	0.1±0.0	=	0.2±0.1	-	<	0.4±0.0	-	<	0.4±0.1	=	0.5±0.0
20:3ω-6	0.8±0.5	=	1.1±0.3	0.6±0.0	=	0.8±0.4	=	0.9±0.1	0.9±0.1	=	1.0±0.0	0.9±0.1	=	0.4±0.1	>	-
20:4ω-6	17.5±3.7	=	17.6±3.5	17.4±2.2	=	19.6±0.1	=	17.5±1.8	10.4±0.5	>	7.8±0.9	11.6±2.1	>	5.3±1.7	>	2.2±0.7
21:1ω-7	-	<	+	-	<	+	=	+	-	=	-	-	=	-	<	0.9±0.0
20:3ω-3	0.1±0.0	=	0.1±0.0	0.1±0.0	=	0.1±0.1	=	0.1±0.0	-	=	-	-	=	-	=	-
i-22:0	-	=	-	-	<	+	=	0.1±0.0	-	=	-	-	<	+	=	+

20:4ω-3	1.5±0.4	>	0.7±0.1	0.9±0.4	=	1.7±0.8	=	2.6±1.0	0.9±0.1	=	1.1±0.8	1.1±0.3	=	0.9±0.4	=	2.0±2.4
20:5ω-3	6.6±0.1	<	9.3±1.6	7.6±0.4	<	13.5±1.1	=	12.0±1.2	7.7±0.1	>	4.3±0.7	8.8±1.6	>	4.7±2.3	=	2.3±1.9
SFA	26.0±2.0	=	24.1±2.2	23.4±1.7	=	23.3±1.8	=	27.7±2.0	37.8±3.6	=	43.5±2.7	36.9±6.1	=	46.6±2.3	=	46.9±7.3
MUFA	9.7±1.3	=	10.7±1.0	9.0±0.3	=	7.8±1.1	=	9.8±0.9	16.2±0.4	=	18.5±1.0	12.7±3.0	<	19.0±3.0	=	22.6±2.2
PUFA	61.9±3.2	=	64.8±1.8	66.2±2.2	=	67.4±2.9	>	60.8±2.9	42.4±4.0	>	31.4±2.7	46.9±7.5	>	28.1±8.8	=	19.2±11.5
ω-3	35.1±1.1	=	36.8±6.0	39.9±0.1	=	39.9±2.7	=	34.9±1.0	19.2±1.2	>	12.1±3.1	22.2±2.9	>	14.2±6.5	=	9.2±9.2
ω-6	19.7±4.6	=	20.7±3.7	19.3±2.4	=	20.8±0.7	=	19.2±1.9	14.6±1.7	>	10.7±0.2	15.9±3.3	>	6.6±1.6	=	2.7±0.6

Table S3. The molecular species composition of MGDG, DGDG, SQDG, PG, PE, PC, PI, PHEG and TAG of various parts of the blades of *Undaria pinnatifida* samples uninfected and infected with the endophyte *Laminariocolax aecidioides* collected in different months. Values given as % of total molecular species (mean ± s.d. for triplicate). "+" – amount less than 0.1%, "-" – not detected. Between columns result of Tukey test and Students t-test (p<0.05, n=3) are shown (">", "<" – statistically significant difference, "=" – no significant changes). "T" – difference between «June» and «February», «S» – difference between uninfected and infected samples.

MGDG	The lower part of the blades of infected *U. pinnatifida* samples												The upper intact part of the blades of infected *U. pinnatifida* samples												The upper part of the blades of infected *U. pinnatifida* samples with endophyte		The lower part of the blades of uninfected *U. pinnatifida* samples				The upper part of the blades of uninfected *U. pinnatifida* samples			
	Nov.		Dec.		Jan.		Feb.		Apr.		Jun.	T	Nov.		Dec.		Jan.		Feb.		Apr.		Jun.	T	Nov.	Jun.	Nov.	S	Jun.	S	Nov.	S	Jun.	S
14:0/16:4	+	=	+	=	+	=	+	=	+	=	+	=	+	=	0.1±0.0	>	+	=	0.1±0.1	>	+	=	+	=	0.1±0.1	0.1±0.0	+	=	+	=	0.2±0.0	>	+	=
16:3/14:0	+	=	+	=	+	=	+	<	+	=	+	=	+	=	+	>	+	=	+	=	+	=	+	=	+	+	+	=	+	=	0.1±0.0	=	+	=
14:0/16:2	+	=	+	=	+	=	+	=	+	=	+	=	+	=	+	=	+	=	+	=	+	=	+	=	+	+	+	=	-	=	+	=	-	=
14:0/16:2	+	=	+	=	+	=	+	=	+	=	+	=	+	=	+	>	-	<	+	>	+	>	-	<	+	+	+	=	-	=	+	=	+	=
14:0/16:1	0.1±0.0	=	0.1±0.0	=	0.1±0.0	<	0.1±0.0	=	0.1±0.0	=	0.1±0.0	<	0.1±0.1	=	0.1±0.0	>	0.1±0.0	=	0.1±0.0	=	0.1±0.0	>	+	<	0.2±0.0	0.1±0.0	0.1±0.0	>	+	=	0.4±0.0	>	+	=
14:0/16:0	+	=	+	=	+	=	+	=	+	<	0.1±0.0	>	+	=	+	>	+	=	+	=	+	<	+	>	+	0.1±0.1	0.1±0.0	>	0.1±0.0	=	+	=	+	=
16:4/16:4	+	=	+	=	+	=	+	=	+	<	+	=	0.1±0.1	=	0.4±0.3	>	+	=	+	=	+	=	0.1±0.1	=	0.1±0.2	0.1±0.0	+	=	-	=	0.6±0.0	>	+	=
16:3/16:4	+	=	0.1±0.1	=	+	=	0.1±0.1	=	+	>	+	=	0.1±0.1	<	0.6±0.2	>	0.1±0.0	=	0.1±0.1	>	+	=	+	=	0.2±0.1	0.1±0.1	0.1±0.1	=	+	=	0.5±0.1	>	+	=
16:3/16:3	+	=	0.1±0.1	=	+	<	0.1±0.0	>	+	=	+	<	0.1±0.1	=	0.3±0.1	>	+	=	0.1±0.1	=	+	<	+	=	0.2±0.1	0.1±0.1	0.1±0.0	>	+	=	0.4±0.1	=	+	=
16:2/16:4	+	=	+	=	+	>	-	<	+	=	+	>	+	<	+	=	+	=	+	=	+	=	+	=	+	+	+	=	-	=	+	=	-	=
18:4/14:1+ 16:2/16:3	0.1±0.0	=	0.1±0.0	>	+	=	+	=	+	=	+	<	+	<	0.1±0.1	>	+	=	+	=	+	=	+	=	+	0.1±0.1	0.1±0.0	=	+	=	0.1±0.0	>	+	=
16:1/16:4+ 14:1/18:4	0.1±0.0	<	0.1±0.0	=	0.2±0.1	>	0.1±0.0	=	0.1±0.0	>	+	=	0.1±0.0	<	0.2±0.0	>	0.1±0.0	=	+	=	+	=	+	=	0.1±0.1	0.1±0.0	0.1±0.0	=	0.1±0.0	=	0.3±0.0	>	+	=
18:3/14:1	+	=	+	=	+	=	+	=	+	=	+	=	+	=	+	>	+	=	+	=	+	=	+	=	+	+	+	=	+	=	0.1±0.0	=	+	=
16:2/16:2	+	=	+	=	+	=	+	=	+	=	+	=	+	=	+	=	+	=	+	>	+	<	+	=	+	+	+	=	+	=	0.1±0.0	=	+	<
16:1/16:3	+	>	-	<	+	=	+	=	+	>	-	=	+	=	-	=	-	<	+	>	-	=	-	<	-	+	+	=	-	=	-	=	+	=
14:0/18:4	1.7±0.2	>	1.1±0.1	=	1.4±0.3	=	0.9±0.5	=	1.2±0.2	=	1.0±0.6	=	1.0±0.1	>	0.4±0.1	=	0.3±0.1	>	0.1±0.1	<	0.2±0.0	=	0.3±0.2	=	0.7±0.1	0.5±0.1	1.8±0.0	=	0.7±0.0	=	0.6±0.1	<	0.3±0.2	=
16:0/16:4	+	=	+	=	+	>	+	=	+	<	+	=	+	=	+	=	+	=	+	=	+	=	+	=	+	0.1±0.0	+	=	+	=	0.1±0.0	=	-	=
16:2/16:1	+	=	+	=	+	=	+	=	+	=	+	=	+	=	+	=	+	<	+	>	+	<	+	=	1.3±1.8	+	-	=	+	=	+	=	-	=
16:1/16:2	+	=	+	>	+	=	+	=	+	=	+	=	+	=	+	>	+	=	0.3±0.4	=	+	=	+	=	+	+	+	=	+	=	0.1±0.0	>	+	=
14:0/18:3	3.1±0.4	>	2.4±0.2	=	2.4±0.5	=	1.9±1.0	<	3.3±0.3	>	1.2±0.2	=	3.1±0.3	>	2.0±0.2	=	1.4±0.4	>	0.7±0.1	<	1.4±0.2	=	1.2±0.5	=	2.8±0.3	1.4±0.3	4.4±0.0	>	1.1±0.1	=	3.1±0.0	=	1.4±0.0	=
16:3/16:0	+	=	+	=	+	=	+	<	+	=	0.1±0.0	>	+	=	+	=	+	=	+	<	+	=	+	=	+	+	+	=	0.1±0.0	=	-	=	+	=

16:0/16:3	-	<	+	=	+	=	+	=	+	=	+	=	-	<	+	>	+	=	+	=	+	=	+	=	+	+	+	=	+	=	+	=	-	=
16:1/16:1	+	=	+	=	+	=	+	=	+	<	+	>	0.1±0.0	=	+	=	+	=	+	>	+	=	+	=	0.1±0.1	+	0.1±0.0	=	+	=	0.2±0.0	=	+	=
14:0/18:2	1.6±0.3	=	1.4±0.1	=	1.5±0.3	<	2.4±0.7	=	3.0±0.1	>	1.5±0.4	=	1.3±0.0	=	1.3±0.2	=	1.0±0.2	=	1.1±0.2	<	1.4±0.2	=	1.3±0.1	=	1.2±0.3	1.0±0.1	2.0±0.2	=	1.2±0.2	=	1.5±0.0	>	1.1±0.1	=
14:0/18:2	+	=	+	=	+	>	-	<	+	=	+	>	+	>	+	=	+	=	+	<	+	=	+	>	+	-	+	=	+	=	+	=	+	=
16:0/16:2	+	=	+	=	+	=	+	=	+	=	+	=	+	=	+	=	+	=	+	=	+	=	+	=	-	+	+	=	+	=	+	=	+	=
14:0/18:1	2.3±0.5	>	1.3±0.2	>	0.9±0.2	<	2.1±0.2	=	2.0±0.2	=	1.9±0.9	=	1.9±0.1	>	1.5±0.2	>	1.1±0.0	<	1.6±0.3	>	1.0±0.1	=	0.9±0.4	<	1.9±0.0	1.1±0.3	3.1±0.4	=	1.3±0.0	=	2.4±0.1	>	0.7±0.1	=
16:0/16:1	+	>	+	=	+	<	+	=	+	=	+	=	+	=	+	=	+	>	-	<	+	=	+	>	+	+	+	=	+	=	+	=	+	=
16:0/16:0	+	>	-	<	+	=	+	=	+	=	+	=	+	>	-	<	+	>	-	=	-	<	+	>	+	+	+	=	+	=	+	=	+	=
15:2/18:4	+	=	+	=	0.1±0.0	=	+	=	+	=	+	=	+	=	0.1±0.0	=	0.1±0.0	=	+	>	+	>	-	<	+	-	+	=	-	=	+	=	-	=
15:1/18:4	+	=	0.1±0.0	=	0.1±0.0	=	0.1±0.0	=	0.1±0.0	=	+	<	0.1±0.0	<	0.1±0.0	=	0.1±0.0	>	0.1±0.0	=	0.1±0.0	>	+	<	0.1±0.0	+	0.1±0.0	=	+	=	0.1±0.0	=	+	=
16:1/17:3	+	=	+	<	+	=	+	=	+	=	+	=	+	=	+	=	+	=	+	<	+	=	+	=	+	+	+	=	+	=	+	=	+	=
15:0/18:4	+	=	+	=	+	=	+	=	+	=	+	=	+	>	+	>	+	=	+	=	+	=	+	=	+	+	+	=	+	=	+	=	+	=
15:0/18:4	0.1±0.0	>	+	<	+	>	+	=	+	=	+	>	+	=	+	>	+	=	+	=	+	<	+	>	+	+	+	=	+	=	+	=	+	=
18:2/15:1	+	=	+	=	+	=	0.1±0.0	=	0.1±0.0	>	+	<	+	<	+	=	+	=	+	=	+	=	+	=	+	+	+	=	+	=	+	=	+	=
18:3/15:0	+	=	+	=	+	=	+	>	-	<	+	=	+	=	+	=	+	>	+	<	+	=	+	=	+	+	+	=	+	=	+	=	-	=
15:0/18:3	+	=	0.1±0.0	=	0.1±0.0	=	+	=	0.1±0.0	>	+	=	0.1±0.0	>	0.1±0.0	=	0.1±0.0	>	+	<	+	<	0.1±0.0	>	0.1±0.0	0.2±0.1	0.1±0.0	=	+	=	0.2±0.0	>	0.1±0.1	=
16:0/17:3	-	=	-	<	+	=	+	>	-	<	+	=	-	=	-	=	+	=	+	<	+	=	+	=	+	+	+	=	-	=	-	=	-	=
15:0/18:2+ 18:2/15:0	+	=	+	=	+	=	0.1±0.0	=	+	=	+	=	+	=	0.1±0.0	=	0.1±0.0	>	+	<	0.1±0.0	>	+	=	0.1±0.0	0.1±0.0	0.1±0.0	>	0.1±0.0	=	0.1±0.0	=	0.1±0.0	=
15:0/18:1	0.1±0.0	=	+	=	+	<	0.1±0.0	>	+	<	0.1±0.0	>	0.1±0.0	=	0.1±0.0	>	+	=	+	=	0.1±0.0	<	0.1±0.0	>	0.1±0.0	0.1±0.0	0.1±0.0	=	0.1±0.0	=	0.1±0.0	=	+	=
18:5/16:4	+	=	+	=	+	>	-	=	-	<	+	>	+	<	+	=	+	=	+	=	+	=	+	=	+	-	+	=	+	=	+	=	-	=
16:4/18:4	0.4±0.1	<	2.0±0.1	<	2.6±0.4	>	1.2±0.1	=	1.5±0.2	>	0.2±0.1	<	0.4±0.1	<	1.5±0.2	=	1.2±0.2	>	0.6±0.1	<	1.1±0.2	>	0.3±0.2	<	0.3±0.1	0.2±0.1	0.6±0.0	=	0.3±0.1	=	0.5±0.1	=	0.4±0.1	=
16:3/18:5	+	>	-	=	-	=	-	<	+	=	+	>	+	>	-	=	-	=	-	<	+	=	+	>	+	0.2±0.1	-	=	+	=	+	=	+	=
16:3/18:4	0.3±0.1	<	0.9±0.1	<	1.3±0.2	=	1.1±0.1	=	1.0±0.1	>	0.4±0.1	<	0.4±0.0	<	0.7±0.1	=	0.8±0.1	>	0.5±0.2	<	0.8±0.2	>	0.3±0.1	=	0.3±0.1	0.2±0.0	0.4±0.0	=	0.4±0.1	=	0.4±0.0	=	0.5±0.1	=
18:3/16:4	-	<	+	>	-	<	+	=	+	=	+	=	+	=	+	>	-	<	+	=	+	<	0.1±0.0	>	+	0.1±0.1	+	=	+	=	+	=	0.1±0.0	=
18:4/16:3	+	=	+	=	+	=	+	=	+	=	+	=	+	=	+	=	+	>	-	<	+	<	0.1±0.0	>	+	0.1±0.1	+	=	+	=	+	=	+	=
16:3/18:3	0.1±0.0	=	0.2±0.0	<	0.3±0.0	=	0.3±0.1	=	0.4±0.1	>	0.2±0.1	<	0.1±0.1	=	0.2±0.0	=	0.2±0.1	=	0.2±0.1	<	0.3±0.1	=	0.2±0.1	=	0.1±0.0	0.2±0.0	0.1±0.0	=	0.2±0.0	=	0.2±0.0	=	0.1±0.1	=
16:2/18:4	0.2±0.0	<	0.3±0.1	=	0.4±0.0	<	0.4±0.0	>	0.3±0.0	>	0.2±0.1	<	0.3±0.0	<	0.4±0.0	=	0.4±0.0	=	0.3±0.1	=	0.3±0.1	=	0.1±0.1	<	0.1±0.0	0.1±0.1	0.3±0.1	=	0.3±0.0	=	0.2±0.1	=	0.2±0.1	=
18:3/16:2	+	<	0.1±0.0	<	0.1±0.0	=	0.1±0.0	=	0.1±0.1	=	0.1±0.1	=	+	=	0.1±0.0	=	0.1±0.0	>	+	<	0.1±0.0	=	0.1±0.0	=	+	+	+	=	0.1±0.0	=	+	=	0.1±0.0	=
16:1/18:4	0.4±0.1	<	0.7±0.1	=	0.7±0.0	=	1.0±0.3	=	0.7±0.2	=	0.6±0.1	<	0.4±0.1	<	0.6±0.1	=	0.5±0.2	=	0.6±0.2	=	0.6±0.1	>	0.2±0.1	<	0.2±0.1	0.1±0.1	0.7±0.0	=	0.5±0.1	=	0.4±0.0	=	0.3±0.3	=
16:1/18:4	0.2±0.1	<	0.5±0.1	<	0.8±0.1	>	0.5±0.2	=	0.4±0.1	>	0.2±0.0	<	0.2±0.1	<	0.4±0.2	=	0.4±0.1	=	0.3±0.1	=	0.4±0.1	>	0.1±0.0	=	0.1±0.0	0.1±0.0	0.2±0.1	=	0.1±0.0	=	0.2±0.1	=	0.1±0.0	=
18:1/16:4	0.2±0.1	>	-	=	-	=	-	<	0.1±0.0	=	0.2±0.1	>	0.1±0.1	=	-	=	-	=	-	<	+	<	0.1±0.0	>	0.1±0.0	0.1±0.0	0.1±0.0	=	0.1±0.0	=	0.1±0.0	=	0.1±0.0	=
20:5/14:0	+	=	+	=	+	=	0.1±0.1	=	0.1±0.0	>	+	=	+	=	+	=	+	=	+	<	0.1±0.0	>	+	=	0.1±0.0	0.1±0.0	+	=	+	=	0.1±0.0	=	+	=
14:0/20:5	+	=	+	=	+	=	+	=	+	=	+	=	+	>	+	<	+	=	+	>	+	<	0.1±0.0	>	0.1±0.0	0.1±0.0	+	=	-	=	0.1±0.0	=	+	=
16:2/18:2	0.1±0.0	<	0.3±0.1	=	0.2±0.0	=	0.3±0.2	=	0.3±0.1	>	0.1±0.0	=	0.2±0.0	<	0.3±0.1	>	0.2±0.0	>	0.1±0.0	=	0.2±0.1	=	0.1±0.0	=	0.1±0.1	0.1±0.0	0.2±0.1	=	0.2±0.1	=	0.1±0.0	=	0.1±0.1	=
16:1/18:3	0.1±0.0	=	-	=	0.1±0.1	=	0.2±0.1	=	0.1±0.0	=	0.1±0.0	=	0.1±0.0	>	-	<	+	=	0.1±0.1	>	-	<	0.1±0.0	=	0.1±0.0	+	+	=	0.1±0.0	=	+	=	0.1±0.0	=
18:1/16:3	0.1±0.0	=	0.1±0.0	=	0.1±0.1	=	+	=	+	<	0.1±0.0	>	0.1±0.0	=	0.1±0.0	>	0.1±0.0	>	+	<	0.1±0.0	=	+	=	0.1±0.0	0.1±0.0	0.1±0.0	=	0.1±0.0	=	0.2±0.0	=	0.1±0.1	=
14:0/20:4	+	=	+	=	+	=	0.1±0.1	=	0.1±0.1	=	0.1±0.0	=	+	>	+	=	+	=	+	=	+	=	+	=	+	0.1±0.1	-	=	+	=	-	=	0.1±0.0	=
16:0/18:4	3.2±0.2	>	2.6±0.5	=	3.1±0.4	=	2.2±0.9	=	2.1±0.1	=	2.5±1.3	=	1.3±0.1	>	0.8±0.4	=	0.7±0.2	=	0.4±0.2	=	0.6±0.1	=	0.9±0.6	=	1.2±0.1	1.6±0.2	3.0±0.5	=	1.7±0.5	=	0.9±0.0	<	0.7±0.1	=
16:1/18:2	0.1±0.1	<	0.2±0.0	=	0.2±0.0	<	0.4±0.1	>	0.1±0.0	>	0.1±0.0	<	+	<	0.1±0.0	=	0.1±0.0	=	0.1±0.0	=	0.1±0.1	>	+	<	+	+	0.1±0.0	=	0.1±0.0	=	0.1±0.0	=	+	=
18:1/16:2	+	=	+	=	+	<	0.1±0.0	=	0.1±0.0	>	+	<	+	<	0.1±0.0	=	+	=	0.1±0.0	=	+	=	0.1±0.0	=	+	+	+	=	+	=	0.1±0.0	>	+	=
14:0/20:3	1.2±2.0	=	+	=	+	=	+	=	0.1±0.0	>	+	=	+	=	+	=	+	=	+	<	0.1±0.0	=	+	=	+	+	+	=	+	=	-	=	-	=
16:0/18:3	3.5±0.2	=	4.4±0.8	=	4.1±0.5	=	3.1±1.4	=	3.3±0.1	>	2.0±0.7	=	2.8±0.4	=	2.6±0.8	=	2.2±0.4	>	1.5±0.4	<	2.1±0.2	=	2.0±0.6	=	3.1±0.5	2.8±0.4	4.5±0.6	=	2.0±0.4	=	3.4±0.0	=	2.2±0.5	=

16:1/18:1	0.1±0.0	=	0.1±0.0	>	+	<	0.1±0.0	=	0.1±0.0	=	+	=	+	=	0.1±0.0	=	0.1±0.0	=	0.1±0.0	=	+	=	+	<	+	+	0.1±0.0	=	+	=	+	=	+	=
16:0/18:2	1.8±0.4	<	2.6±0.3	=	2.9±0.4	=	4.0±0.9	=	3.8±0.3	>	2.1±0.4	<	1.5±0.0	<	2.2±0.5	=	2.0±0.2	<	2.5±0.3	=	2.5±0.2	>	1.6±0.4	<	1.8±0.2	1.7±0.5	1.9±0.2	=	1.8±0.3	=	1.9±0.1	>	1.6±0.6	=
16:0/18:2	+	=	+	=	+	=	+	=	+	=	+	=	+	=	+	=	+	=	+	=	+	=	+	=	+	+	+	=	+	=	+	=	-	=
18:1/16:1	+	=	+	=	+	<	+	=	+	=	+	=	+	=	+	=	+	=	+	=	+	=	+	=	+	+	+	=	+	=	+	=	-	=
16:0/18:1	3.0±0.5	=	2.3±0.3	=	2.3±0.4	<	3.8±0.6	>	2.8±0.1	=	2.9±1.3	=	2.6±0.1	=	2.8±0.3	=	2.5±0.5	<	4.0±0.4	>	2.3±0.3	>	1.3±0.4	<	3.6±0.4	2.2±0.5	3.3±0.2	=	2.4±0.1	=	3.5±0.3	=	1.3±0.5	=
16:0/18:1	+	=	+	=	+	=	+	=	+	>	-	<	+	=	+	=	+	=	+	=	+	=	+	=	+	-	-	=	+	=	+	=	+	=
16:0/18:0	-	=	-	<	+	>	-	<	+	=	+	>	+	=	-	=	+	>	-	=	+	=	+	>	+	+	+	=	+	=	+	=	-	=
20:5/15:0	+	>	-	<	+	=	+	=	+	>	+	=	+	>	-	<	+	>	-	=	+	=	+	>	+	+	+	=	+	=	+	=	+	=
15:0/20:5	+	>	+	=	+	=	+	=	+	=	+	<	+	>	+	=	+	>	+	=	+	>	-	<	+	+	+	=	-	=	+	<	+	=
15:0/20:4	+	=	+	=	+	=	+	>	+	=	+	=	+	=	+	=	+	=	+	=	+	=	+	=	+	+	+	=	+	=	+	=	+	=
17:0/18:4	+	=	+	=	+	=	+	<	0.1±0.0	=	+	=	+	=	+	=	+	=	+	=	+	=	+	=	+	+	+	=	+	=	+	=	+	=
17:0/18:3	+	>	+	=	+	=	+	=	+	=	+	=	+	>	+	=	+	=	+	=	+	<	+	>	+	+	+	=	+	=	+	=	+	=
17:1/18:1	+	=	-	=	-	<	+	>	-	<	+	=	-	=	-	=	-	=	-	=	-	=	-	=	-	+	+	=	+	=	+	=	-	=
17:0/18:2	+	=	+	=	+	=	+	=	+	=	+	=	+	=	+	=	+	=	+	=	+	=	+	=	+	+	+	=	+	=	+	=	+	=
16:0/19:1	-	=	-	=	-	<	+	>	-	<	+	=	+	>	-	<	+	>	-	<	+	>	-	=	+	+	-	=	-	=	-	=	+	=
17:0/18:1	+	>	+	=	+	=	+	=	+	=	+	=	+	<	+	>	+	=	+	<	+	<	+	>	+	+	+	=	+	=	+	=	+	=
18:5/18:5	+	<	+	>	-	<	+	>	-	=	-	<	-	<	+	>	-	=	-	=	-	<	+	>	+	-	+	=	-	=	+	=	+	=
18:5/18:4	0.1±0.0	<	0.4±0.0	>	0.4±0.0	>	0.2±0.0	=	0.2±0.1	>	0.1±0.0	<	0.2±0.0	<	0.6±0.0	>	0.5±0.0	>	0.2±0.0	=	0.2±0.0	>	0.1±0.1	<	0.2±0.0	0.2±0.1	0.2±0.0	=	0.1±0.0	=	0.2±0.1	=	0.2±0.1	=
20:5/16:4	0.2±0.1	=	0.4±0.3	=	0.3±0.1	=	0.2±0.2	=	0.1±0.0	>	0.1±0.0	=	0.4±0.3	<	1.5±0.8	>	0.3±0.1	=	0.5±0.3	=	0.3±0.1	>	0.2±0.0	=	0.7±0.4	0.6±0.3	0.4±0.2	=	0.1±0.0	=	2.4±0.1	>	0.1±0.0	<
18:4/18:4	30.7±3.1	<	38.4±3.1	=	39.5±0.5	>	31.5±2.5	=	32.0±2.4	>	20.0±4.4	<	34.8±4.1	=	33.9±2.8	=	34.6±3.7	=	31.6±1.9	>	27.4±2.2	>	22.8±2.0	<	36.2±2.7	30.2±1.8	27.0±5.8	=	20.5±1.3	=	29.4±0.6	=	26.3±7.4	=
18:3/18:4	9.8±1.3	>	4.7±0.3	>	3.1±0.6	=	3.6±1.0	=	4.1±0.2	<	9.6±2.2	>	11.1±0.4	>	6.1±0.5	=	6.9±0.6	>	5.6±0.6	=	5.9±0.2	<	14.6±1.9	>	9.4±0.8	10.8±2.3	9.1±1.1	=	10.8±2.4	=	10.1±0.3	=	11.9±2.4	=
18:4/18:3	3.0±0.7	=	3.2±0.6	=	3.5±1.7	=	4.6±1.0	=	5.0±0.5	=	5.4±1.3	=	1.7±0.2	<	2.6±0.6	=	2.4±0.8	=	3.0±0.6	=	3.0±0.5	=	2.2±0.6	=	1.8±0.4	3.3±0.5	3.8±0.0	=	4.1±0.8	=	1.9±0.2	=	3.1±1.3	=
18:3/18:3	1.5±0.4	>	1.0±0.1	=	0.8±0.1	=	1.0±0.5	=	0.8±0.1	<	2.1±1.1	=	2.5±0.3	>	1.4±0.2	<	2.2±0.3	>	1.8±0.1	<	2.3±0.3	<	4.2±1.3	>	2.0±0.3	2.5±0.8	1.5±0.2	=	2.7±0.0	=	2.7±0.1	=	3.6±1.2	=
18:3/18:3	0.8±0.0	=	0.3±0.0	=	0.5±0.0	=	0.8±0.2	=	0.5±0.4	=	1.6±0.0	=	-	<	0.3±0.0	>	-	<	0.7±0.0	=	0.9±0.3	=	0.9±0.4	=	0.4±0.0	-	-	=	-	=	-	=	-	=
18:2/18:4	1.1±0.7	=	0.4±0.2	=	0.4±0.1	=	0.4±0.2	=	0.7±0.5	<	3.4±0.7	>	0.8±0.2	>	0.5±0.1	=	0.9±0.4	=	1.2±0.3	=	0.9±0.6	=	1.1±0.4	=	0.9±0.2	1.9±0.5	1.1±0.2	=	3.0±0.5	=	0.7±0.0	=	2.3±1.2	=
20:5/16:1	0.1±0.0	=	0.1±0.0	=	0.2±0.2	=	0.2±0.1	=	0.2±0.1	=	0.4±0.1	>	0.1±0.1	=	0.1±0.0	>	0.1±0.0	<	0.2±0.0	=	0.2±0.0	=	0.2±0.1	=	0.1±0.1	0.2±0.1	0.1±0.1	=	0.5±0.3	=	0.1±0.0	=	0.2±0.1	=
18:3/18:2	1.4±0.2	=	1.6±0.1	>	1.2±0.2	<	2.0±0.1	>	1.6±0.1	<	3.1±0.7	>	1.3±0.3	=	1.5±0.0	=	1.6±0.1	<	1.9±0.2	=	2.3±0.3	=	2.6±0.2	>	1.4±0.0	1.8±0.3	1.4±0.2	=	3.5±0.3	=	1.3±0.0	=	2.9±1.0	=
18:2/18:3	0.5±0.6	=	0.1±0.0	=	0.1±0.1	=	0.2±0.0	=	0.2±0.0	<	0.7±0.5	>	0.1±0.1	=	0.1±0.0	=	0.1±0.0	<	0.1±0.0	=	0.2±0.0	>	0.1±0.0	=	0.1±0.0	0.4±0.1	0.2±0.0	=	0.6±0.0	=	+	=	0.3±0.0	>
18:4/18:1	0.2±0.0	>	0.1±0.0	=	+	=	+	>	+	<	0.2±0.1	>	0.2±0.0	>	0.1±0.0	=	0.1±0.0	=	0.1±0.0	=	+	<	0.1±0.0	=	0.2±0.1	0.2±0.0	0.2±0.0	=	0.1±0.0	=	0.1±0.1	=	0.1±0.0	=
18:1/18:4	+	=	+	=	+	=	+	=	+	<	0.1±0.0	=	+	=	+	=	+	=	0.1±0.1	=	+	=	+	=	-	+	+	=	+	=	+	=	-	=
20:5/16:0	+	>	+	>	-	<	+	=	+	=	+	=	+	=	+	=	+	=	+	=	+	=	+	=	0.1±0.0	+	+	=	+	=	+	=	+	=
20:5/16:0	+	=	+	=	+	=	+	=	+	=	0.1±0.0	=	+	<	0.1±0.0	=	0.1±0.0	=	0.1±0.0	=	0.1±0.0	=	+	=	0.1±0.1	0.1±0.0	+	=	0.1±0.0	=	0.1±0.0	=	0.1±0.0	=
16:0/20:5	0.1±0.0	=	+	=	+	=	0.1±0.0	=	+	=	0.1±0.0	=	+	=	0.1±0.0	=	0.1±0.0	=	+	<	0.1±0.0	=	0.1±0.0	=	0.1±0.1	0.2±0.1	+	=	+	=	0.1±0.0	=	-	=
18:2/18:2	0.8±0.1	<	1.2±0.1	=	1.1±0.3	=	1.8±0.6	=	1.5±0.1	=	1.6±0.5	=	0.6±0.1	<	1.1±0.1	>	0.9±0.0	<	1.5±0.1	=	1.7±0.4	>	0.6±0.1	<	0.6±0.0	0.7±0.1	1.0±0.2	=	1.9±0.0	=	0.5±0.0	=	0.7±0.1	=
18:3/18:1	0.3±0.1	=	0.3±0.1	>	0.1±0.0	=	0.3±0.2	=	0.2±0.0	=	0.3±0.1	=	0.4±0.1	=	0.4±0.1	=	0.3±0.1	=	0.4±0.2	=	0.3±0.0	=	0.2±0.1	<	0.6±0.1	0.4±0.1	0.4±0.1	=	0.3±0.0	=	0.5±0.0	=	0.2±0.0	=
18:1/18:3	0.3±0.1	=	0.3±0.1	>	0.1±0.0	>	0.1±0.0	=	+	<	0.3±0.1	>	0.4±0.1	=	0.4±0.1	=	0.3±0.1	>	+	>	+	<	0.2±0.1	>	0.6±0.1	0.4±0.1	0.4±0.1	=	0.3±0.0	=	0.5±0.0	=	0.2±0.0	=
16:0/20:4	+	=	+	=	+	<	+	=	+	=	0.1±0.1	=	0.1±0.0	>	+	=	+	=	+	=	+	=	+	=	0.1±0.0	0.1±0.0	+	=	+	=	+	=	0.2±0.3	=
20:4/16:0	0.1±0.0	>	+	=	+	<	+	>	+	=	+	=	+	=	+	=	+	=	+	=	+	<	0.1±0.0	>	0.1±0.0	0.2±0.2	+	=	+	=	0.1±0.1	=	+	=
16:0/20:4	+	=	-	<	+	=	+	>	+	=	+	=	+	>	-	<	+	=	+	=	+	<	+	=	+	+	+	=	+	=	+	=	+	=
20:4/16:0	+	=	+	<	+	=	+	=	+	<	0.1±0.0	>	+	=	+	=	+	=	+	=	+	<	+	>	+	+	+	=	+	=	-	=	+	=
16:0/20:4	0.8±0.4	>	0.2±0.1	=	0.2±0.1	>	0.1±0.0	=	0.2±0.2	=	0.2±0.1	>	0.2±0.1	=	0.1±0.1	=	0.1±0.0	=	0.1±0.1	=	0.1±0.0	<	0.1±0.0	=	0.2±0.0	0.1±0.1	0.6±0.1	=	0.1±0.1	=	0.1±0.0	=	0.2±0.1	=
18:0/18:4	1.1±0.1	>	0.2±0.1	=	0.1±0.1	=	0.1±0.0	=	0.1±0.1	=	0.2±0.1	>	0.1±0.0	>	0.1±0.0	=	0.1±0.1	=	0.1±0.0	=	0.1±0.0	=	0.1±0.1	=	0.1±0.0	0.2±0.1	0.3±0.0	=	0.1±0.1	=	0.2±0.0	=	0.1±0.0	=
18:2/18:1	0.3±0.1	=	0.3±0.0	>	0.2±0.0	<	0.4±0.1	=	0.3±0.1	=	0.4±0.2	=	0.3±0.1	<	0.4±0.0	=	0.4±0.0	<	0.5±0.1	>	0.3±0.0	>	0.1±0.1	<	0.3±0.0	0.3±0.1	0.4±0.1	=	0.4±0.1	=	0.2±0.0	=	0.1±0.0	=

16:0/20:3	-	=	-	=	-	<	+	=	+	>	-	<	-	=	-	=	-	<	+	>	-	=	-	<	-	-	-	=	-	=	-	=	-	=
20:3/16:0	+	>	+	=	+	=	+	=	+	=	+	=	+	>	+	>	-	<	+	=	+	=	+	=	+	+	+	=	+	=	+	=	+	=
16:0/20:3	+	=	+	=	+	=	+	=	+	=	0.1±0.0	=	+	=	+	=	+	>	+	=	+	<	0.1±0.0	>	0.1±0.0	+	+	=	+	=	0.1±0.0	=	+	=
16:0/20:3	0.2±0.0	=	0.1±0.0	>	+	=	+	=	+	<	0.1±0.1	=	0.1±0.0	>	+	=	+	>	-	<	+	=	0.1±0.1	=	0.1±0.0	0.1±0.0	-	=	0.1±0.0	=	0.1±0.0	<	-	=
18:0/18:3	0.3±0.2	>	0.1±0.0	=	0.1±0.0	>	+	=	0.1±0.1	=	0.1±0.1	=	0.1±0.1	=	0.1±0.0	>	+	=	+	=	0.1±0.1	=	0.2±0.2	=	0.1±0.0	0.1±0.0	0.2±0.1	=	0.1±0.0	=	0.1±0.0	=	0.3±0.1	=
18:1/18:1	0.2±0.1	=	0.1±0.0	=	0.1±0.0	<	0.2±0.0	>	0.1±0.0	=	0.1±0.0	=	0.1±0.0	<	0.1±0.0	=	0.1±0.0	=	0.1±0.0	>	0.1±0.0	=	0.1±0.0	<	0.2±0.1	0.1±0.0	0.2±0.0	=	+	=	0.1±0.0	>	+	=
18:0/18:2	0.1±0.0	=	+	=	+	=	+	=	0.1±0.0	>	+	=	+	<	+	=	+	=	+	=	+	=	+	=	+	+	0.1±0.0	=	+	=	+	=	0.1±0.0	=
18:0/18:1	0.2±0.0	>	0.1±0.0	=	0.1±0.0	<	0.1±0.0	=	0.1±0.0	=	0.1±0.0	=	0.1±0.0	=	0.1±0.0	>	0.1±0.0	=	0.1±0.0	>	+	=	+	<	0.1±0.0	0.1±0.0	0.2±0.0	=	+	=	0.1±0.0	=	+	=
18:4/19:2	0.1±0.0	>	+	>	+	=	+	=	+	<	+	=	0.1±0.0	=	0.1±0.0	=	+	=	+	=	+	=	+	=	+	+	0.1±0.0	=	+	=	+	=	+	=
18:3/19:2	+	=	+	=	+	=	+	=	+	=	+	=	+	=	+	=	+	=	+	=	+	=	+	=	+	+	+	=	+	=	+	=	+	=
19:1/18:4	+	=	+	=	+	=	+	<	+	>	+	=	+	=	+	=	+	=	+	=	+	<	+	>	+	+	+	=	+	=	+	=	+	=
18:3/19:1	+	=	+	=	-	<	+	=	+	=	+	=	+	=	+	=	+	=	+	=	+	=	+	=	+	+	+	=	+	=	+	=	+	=
20:5/18:5	0.1±0.0	<	0.1±0.0	=	0.1±0.0	>	0.1±0.0	=	0.1±0.0	>	+	<	0.1±0.0	<	0.1±0.0	=	0.1±0.0	=	0.1±0.0	<	0.1±0.0	>	+	=	+	+	0.1±0.0	=	+	=	+	=	+	=
20:5/18:4	13.7±0.9	<	16.4±0.9	=	15.2±3.6	=	15.1±4.1	=	15.7±0.9	=	14.5±2.1	=	16.4±1.0	<	21.5±1.8	=	24.0±1.8	>	21.4±0.6	<	23.3±1.0	=	19.1±3.7	=	15.8±0.5	17.0±3.9	14.5±0.1	=	14.8±2.3	=	16.9±0.3	=	18.0±2.1	=
20:4/18:4	1.9±0.3	>	1.2±0.1	<	1.4±0.1	=	1.1±0.5	=	0.6±0.0	<	1.6±0.4	=	2.0±0.2	>	0.8±0.1	=	0.8±0.2	=	0.7±0.4	=	0.8±0.2	=	0.8±0.1	=	1.1±0.2	0.7±0.6	2.2±0.3	=	1.0±0.3	=	1.1±0.0	<	1.5±0.7	=
20:5/18:3	0.4±0.2	=	0.3±0.1	=	0.3±0.1	<	0.9±0.5	=	0.6±0.2	=	0.9±0.7	=	1.1±0.2	=	0.9±0.3	<	1.5±0.4	<	2.8±0.9	=	2.3±0.4	=	4.5±2.2	=	1.4±0.2	3.0±0.5	0.3±0.1	=	1.6±0.4	=	1.6±0.5	=	2.9±0.8	=
20:4/18:4	2.8±0.3	>	2.1±0.2	=	2.5±0.3	<	3.6±0.5	=	3.8±0.4	<	9.6±2.8	>	2.5±0.4	=	2.3±0.5	=	2.7±0.6	<	4.6±1.0	=	4.9±0.3	<	5.6±0.4	=	1.8±0.1	2.9±0.7	2.9±0.2	=	11.6±1.9	=	2.0±0.3	=	5.7±2.5	=
20:4/18:3	0.2±0.1	>	+	=	+	=	0.1±0.0	=	0.1±0.0	=	0.1±0.1	=	0.4±0.1	>	0.1±0.0	<	0.1±0.0	<	0.2±0.0	=	0.2±0.1	<	0.5±0.2	>	0.3±0.1	0.4±0.2	0.1±0.0	=	0.2±0.0	=	0.4±0.1	=	0.5±0.1	=
20:3/18:4	1.2±0.1	>	0.4±0.0	=	0.7±0.3	=	0.8±0.2	>	0.3±0.2	<	1.1±0.6	=	0.3±0.1	=	0.2±0.1	=	0.3±0.1	=	0.2±0.0	=	0.3±0.1	=	0.3±0.1	=	0.2±0.1	0.3±0.2	1.2±0.1	=	0.4±0.2	=	0.2±0.0	=	0.3±0.2	=
20:4/18:3	0.4±0.2	=	0.2±0.1	<	0.3±0.0	<	0.5±0.2	=	0.3±0.1	<	0.9±0.4	=	0.4±0.1	>	0.2±0.1	=	0.2±0.2	=	0.6±0.3	=	0.6±0.3	=	1.3±1.0	=	0.3±0.0	0.6±0.4	0.2±0.2	=	1.6±0.8	=	0.4±0.0	=	1.3±1.6	=
20:5/18:2	0.1±0.1	=	0.1±0.0	=	0.1±0.0	=	0.3±0.1	=	0.2±0.1	=	1.1±1.1	=	0.1±0.0	=	0.1±0.0	=	0.1±0.1	<	0.4±0.2	=	0.3±0.1	=	0.8±0.2	=	0.1±0.0	0.3±0.0	-	=	0.8±0.8	=	+	=	-	=
18:3/20:4	0.1±0.0	>	+	=	0.1±0.0	=	0.1±0.1	=	0.1±0.0	<	0.8±0.5	>	0.1±0.0	=	+	=	+	<	0.1±0.0	=	0.1±0.1	=	0.2±0.1	>	0.1±0.0	0.1±0.1	0.1±0.0	=	0.6±0.1	=	+	=	0.3±0.3	=
20:3/18:3	0.2±0.1	>	0.1±0.0	=	0.1±0.0	=	0.1±0.0	=	0.1±0.0	<	0.3±0.0	>	0.2±0.1	>	0.1±0.0	<	0.1±0.0	<	0.2±0.0	=	0.2±0.0	=	0.2±0.1	=	0.1±0.0	0.2±0.1	0.2±0.1	=	0.3±0.0	=	0.1±0.0	=	0.3±0.1	=
20:4/18:2	0.1±0.1	=	0.1±0.0	=	0.1±0.0	<	0.2±0.1	=	0.2±0.1	<	0.7±0.4	>	0.1±0.0	=	0.1±0.0	=	0.1±0.0	<	0.2±0.0	=	0.2±0.1	=	0.4±0.2	=	0.1±0.0	0.2±0.1	0.1±0.0	=	0.5±0.1	=	0.1±0.0	=	0.4±0.1	=
20:5/18:1	+	=	0.1±0.0	=	0.1±0.0	=	0.1±0.1	=	0.1±0.0	=	0.1±0.1	=	0.1±0.0	=	0.1±0.0	=	0.1±0.0	=	0.2±0.1	=	0.1±0.1	=	0.1±0.0	=	0.1±0.0	0.2±0.1	0.1±0.0	=	-	=	0.1±0.0	=	+	<
20:3/18:2	0.1±0.0	>	0.1±0.0	=	0.1±0.0	<	0.2±0.0	=	0.2±0.0	=	0.2±0.0	>	0.1±0.0	>	0.1±0.0	=	0.1±0.0	<	0.2±0.0	=	0.1±0.0	=	0.1±0.0	=	0.1±0.0	0.1±0.0	0.1±0.0	=	0.2±0.0	=	0.1±0.0	<	0.1±0.0	=
20:4/18:1	0.1±0.0	>	+	=	+	<	+	=	+	<	0.1±0.1	=	+	=	+	=	+	=	0.1±0.0	>	+	=	+	=	0.1±0.1	0.1±0.0	0.1±0.0	=	0.1±0.0	=	+	=	+	=
20:5/18:0	+	=	+	=	+	=	+	=	+	=	+	=	+	=	+	=	+	=	+	>	+	<	+	=	+	+	+	=	-	=	+	=	+	<
20:3/18:1	+	=	+	=	+	=	+	=	0.1±0.0	=	0.1±0.0	>	+	=	+	=	+	<	0.1±0.0	=	+	=	0.1±0.0	=	+	0.1±0.0	0.1±0.0	=	+	<	+	=	+	=
20:0/18:4	0.1±0.0	>	0.1±0.0	=	0.1±0.0	=	0.1±0.0	>	+	<	0.1±0.0	>	0.1±0.0	=	0.1±0.0	=	0.1±0.0	=	0.1±0.0	=	0.1±0.0	=	0.1±0.1	=	+	+	0.1±0.0	=	0.1±0.1	=	+	<	0.1±0.0	=
20:0/18:3	+	=	+	<	+	=	+	=	+	<	+	>	+	=	+	>	+	=	+	=	+	=	+	=	+	+	+	=	+	<	+	=	+	=
20:5/20:5	+	<	0.1±0.0	=	0.1±0.0	<	0.4±0.2	=	0.4±0.0	>	+	<	0.2±0.0	=	0.2±0.1	=	0.2±0.1	<	0.5±0.0	<	0.9±0.2	>	0.2±0.1	<	0.3±0.0	0.5±0.1	+	=	+	=	0.2±0.1	=	0.2±0.2	=
20:5/20:4	0.1±0.0	>	+	<	0.1±0.0	=	0.1±0.1	=	0.1±0.0	>	+	<	0.2±0.0	=	0.1±0.1	=	0.1±0.0	<	0.2±0.0	<	0.3±0.1	>	0.2±0.0	=	0.2±0.0	0.2±0.1	0.1±0.0	=	0.1±0.0	=	0.3±0.1	=	0.2±0.2	=
20:4/20:5	0.1±0.0	=	0.1±0.0	=	0.1±0.0	<	0.5±0.3	=	0.7±0.1	>	0.2±0.2	=	0.2±0.1	=	0.2±0.1	=	0.2±0.1	<	1.0±0.1	<	1.6±0.3	>	0.8±0.4	=	0.5±0.1	0.9±0.3	0.1±0.0	=	0.3±0.0	=	0.4±0.1	=	1.1±0.4	=
20:5/20:3	0.1±0.0	=	0.1±0.0	=	0.1±0.0	<	0.2±0.1	=	0.2±0.0	>	0.1±0.0	<	0.1±0.0	=	0.1±0.0	=	0.2±0.1	=	0.3±0.1	<	0.4±0.1	=	0.3±0.1	=	0.1±0.0	0.2±0.1	0.1±0.0	=	0.1±0.0	=	0.1±0.0	=	0.2±0.0	=
20:4/20:4	+	=	+	=	+	=	0.1±0.1	<	0.2±0.0	=	0.5±0.3	=	0.1±0.0	>	0.1±0.0	=	0.1±0.0	<	0.2±0.1	=	0.3±0.0	<	1.3±0.9	>	0.1±0.1	0.7±0.1	0.1±0.0	=	0.6±0.1	=	0.2±0.0	=	1.2±0.1	=
20:4/20:3	-	=	-	=	-	<	+	>	-	<	+	=	+	>	-	=	-	=	-	<	+	=	+	>	-	+	-	=	-	=	-	=	-	=
SFA/SFA	+	>	+	=	+	=	+	=	+	<	0.1±0.0	>	+	=	+	>	+	=	+	=	+	<	+	>	0.1±0.0	0.1±0.1	0.1±0.0	>	0.1±0.1	=	0.1±0.0	=	+	=
SFA/MUFA	5.5±1.0	>	3.8±0.5	=	3.4±0.6	<	6.1±0.8	>	5.0±0.3	=	5.0±2.3	=	4.7±0.1	=	4.5±0.3	>	3.7±0.5	<	5.8±0.6	>	3.5±0.3	>	2.4±0.8	<	5.8±0.4	3.5±0.8	6.6±0.5	=	4.0±0.1	=	6.5±0.4	=	2.1±0.7	=
MUFA/SFA	-	=	-	=	-	=	-	=	-	=	-	=	-	=	-	=	-	=	-	=	-	=	-	=	-	-	-	=	-	=	-	=	-	=
SFA/PUFA	15.4±1.5	>	12.9±1.5	=	13.4±1.7	=	13.1±4.3	=	15.7±0.5	>	8.9±1.9	=	10.9±0.4	=	9.4±1.5	=	7.8±1.1	=	6.6±0.9	<	8.4±0.5	=	7.7±1.5	=	10.7±1.4	9.0±0.7	16.4±1.4	=	7.5±0.0	=	12.2±0.2	>	7.7±1.0	=
PUFA/SFA	0.2±0.0	=	0.1±0.1	=	0.1±0.0	<	0.3±0.0	=	0.3±0.0	=	0.3±0.1	=	0.2±0.1	=	0.3±0.0	=	0.2±0.0	=	0.2±0.0	=	0.3±0.1	=	0.3±0.1	=	0.5±0.1	0.6±0.3	0.2±0.0	=	0.2±0.0	=	0.4±0.1	=	0.3±0.0	=
MUFA/MUFA	0.3±0.1	=	0.2±0.0	>	0.1±0.0	<	0.3±0.0	>	0.2±0.1	=	0.1±0.1	<	0.1±0.0	<	0.2±0.0	=	0.2±0.1	=	0.2±0.0	>	0.1±0.0	=	0.1±0.0	<	0.3±0.0	0.2±0.1	0.3±0.0	=	0.1±0.0	=	0.3±0.0	>	0.1±0.0	=

MUFA/PUFA	1.4±0.5	<	2.1±0.3	=	2.4±0.3	=	2.3±0.4	>	1.7±0.2	=	1.5±0.3	<	1.5±0.2	<	2.1±0.2	>	1.7±0.2	=	1.6±0.2	=	1.4±0.2	>	0.8±0.2	<	1.4±0.4	1.1±0.2	2.0±0.1	=	1.3±0.1	=	1.9±0.1	=	0.9±0.3	=
PUFA/MUFA	1.1±0.3	=	0.9±0.1	=	0.7±0.2	=	1.2±0.5	=	0.9±0.1	<	1.5±0.4	=	1.2±0.3	=	1.2±0.1	=	1.1±0.2	<	1.5±0.2	>	1.0±0.0	>	0.9±0.1	<	2.3±1.4	1.5±0.1	1.3±0.2	=	1.4±0.4	=	1.2±0.2	=	0.8±0.0	=
PUFA/PUFA	76.1±2.0	<	80.0±2.0	=	80.0±2.2	=	76.8±3.1	=	76.4±0.2	<	82.7±4.7	=	81.3±0.6	=	82.3±1.6	<	85.3±1.2	=	84.1±1.6	=	85.3±0.4	=	88.0±2.4	>	79.1±1.5	84.1±1.0	73.2±2.3	=	85.4±0.5	=	77.6±0.7	<	88.0±1.3	=

DGDG	The lower part of the blades of infected *U. pinnatifida* samples												The upper intact part of the blades of infected *U. pinnatifida* samples												The upper part of the blades of infected *U. pinnatifida* samples with endophyte		The lower part of the blades of uninfected *U. pinnatifida* samples				The upper part of the blades of uninfected *U. pinnatifida* samples			
	Nov.		Dec.		Jan.		Feb.		Apr.		Jun.	T	Nov.		Dec.		Jan.		Feb.		Apr.		Jun.	T	Nov.	Jun.	Nov.	S	Jun.	S	Nov.	S	Jun.	S
14:0/16:2	+	=	+	=	+	=	+	=	+	=	0.1±0.0	=	0.1±0.1	=	+	=	+	=	+	=	+	=	0.1±0.0	>	+	+	+	=	-	=	0.1±0.0	=	0.1±0.0	=
14:0/16:1	0.1±0.0	=	+	=	+	=	+	=	0.1±0.0	=	0.1±0.0	=	0.1±0.1	=	0.1±0.0	=	+	=	+	=	0.1±0.0	=	0.1±0.0	=	0.3±0.2	0.1±0.0	0.1±0.0	=	0.2±0.0	=	0.5±0.0	>	0.1±0.1	=
14:0/16:0	0.1±0.0	>	-	=	-	<	+	=	+	=	0.1±0.1	=	+	=	-	=	+	=	+	=	+	>	-	<	0.1±0.0	0.1±0.0	0.1±0.0	=	0.1±0.0	=	0.1±0.1	=	+	=
16:1/16:4	0.1±0.0	>	+	=	+	=	0.1±0.0	=	+	=	0.1±0.2	=	0.1±0.1	=	+	=	0.1±0.0	=	0.1±0.0	=	0.1±0.0	=	0.1±0.0	=	0.1±0.1	0.2±0.0	+	=	-	=	0.1±0.1	=	+	=
16:2/16:2	+	=	+	=	+	=	0.1±0.0	=	+	>	-	<	+	=	0.1±0.0	>	+	=	+	>	+	>	-	<	+	-	-	=	-	=	0.1±0.0	=	0.1±0.0	=
16:3/16:1	+	>	-	=	-	<	+	=	+	>	-	<	+	=	-	=	+	=	+	>	+	<	0.2±0.0	>	+	-	-	=	-	=	+	=	0.1±0.0	=
14:0/18:4	2.0±0.4	=	2.7±1.0	<	4.8±0.8	=	3.8±1.7	=	3.0±0.2	>	0.5±0.1	<	1.9±0.1	=	2.0±0.5	=	2.0±0.3	>	1.3±0.5	=	1.0±0.2	=	0.6±0.4	=	1.7±0.4	0.4±0.2	2.9±0.1	>	0.9±0.6	=	1.9±0.0	=	0.5±0.4	=
16:1/16:2	+	>	-	<	+	=	+	=	+	>	-	<	-	=	-	=	+	=	1.2±1.6	=	+	<	0.1±0.0	=	+	-	+	=	-	=	0.1±0.1	=	-	=
14:0/18:3	4.0±1.3	>	1.4±0.7	<	2.6±0.2	=	2.8±0.6	=	3.7±0.6	=	3.6±0.7	=	6.4±0.9	>	3.7±0.9	=	3.8±0.4	>	2.1±0.3	=	2.5±0.6	=	4.1±2.0	=	5.5±0.5	3.3±0.4	4.9±0.6	=	3.0±0.8	=	5.4±1.0	=	4.4±2.0	=
16:1/16:1	+	=	+	>	+	<	+	>	+	>	-	<	0.1±0.1	=	+	=	+	=	+	=	-	<	0.2±0.0	>	0.2±0.1	0.2±0.2	+	=	-	=	0.3±0.1	>	0.1±0.0	=
14:0/18:2	0.4±0.1	>	0.2±0.1	=	0.3±0.2	<	0.8±0.1	=	0.7±0.1	=	1.2±0.8	=	0.3±0.0	<	0.4±0.0	=	0.3±0.1	=	0.3±0.1	=	0.4±0.2	=	0.6±0.3	=	0.5±0.2	0.6±0.0	0.3±0.0	=	0.7±0.4	=	0.5±0.1	=	0.5±0.2	=
14:0/18:1	0.3±0.1	=	0.1±0.0	=	0.1±0.1	=	0.2±0.1	=	0.1±0.0	<	0.6±0.2	>	0.2±0.1	=	0.2±0.1	=	0.1±0.0	<	0.3±0.1	=	0.2±0.0	=	0.3±0.1	=	0.6±0.0	0.5±0.1	0.2±0.0	=	0.4±0.2	=	0.6±0.0	>	0.2±0.3	=
16:0/16:1	+	>	-	=	-	<	+	=	+	=	+	=	-	=	-	=	-	=	-	=	-	=	-	=	0.1±0.0	-	+	=	-	=	-	=	-	=
16:0/16:0	-	=	-	=	-	=	-	=	-	=	-	=	-	=	-	=	-	=	-	=	-	=	-	=	-	-	-	=	-	=	-	=	+	=
15:0/18:4	+	=	0.1±0.0	=	0.1±0.0	=	0.1±0.1	=	+	<	0.2±0.0	=	0.1±0.0	=	0.1±0.0	=	0.1±0.0	>	+	=	+	=	0.1±0.1	=	0.1±0.0	0.1±0.0	0.1±0.0	=	-	=	0.1±0.0	=	-	=
15:0/18:3	0.1±0.0	>	0.1±0.0	=	0.1±0.0	=	0.1±0.0	<	0.1±0.0	=	0.1±0.0	=	0.2±0.0	=	0.1±0.0	=	0.1±0.1	>	+	<	0.1±0.0	<	0.4±0.1	>	0.2±0.0	0.3±0.1	0.1±0.1	=	0.2±0.0	=	0.2±0.1	=	0.4±0.0	=
15:0/18:2	-	=	-	<	+	=	+	>	+	>	-	<	0.1±0.0	>	-	<	+	=	+	=	+	>	-	<	+	0.1±0.0	-	=	0.2±0.0	=	+	=	+	=
18:3/16:2	+	=	+	<	0.1±0.0	=	0.2±0.1	>	0.1±0.0	=	+	<	+	<	0.1±0.0	<	0.1±0.0	=	0.1±0.0	<	0.3±0.1	=	0.2±0.1	=	+	0.2±0.0	+	=	-	=	+	=	0.1±0.0	=
16:2/18:3	-	=	-	=	-	<	0.3±0.0	>	-	=	-	<	-	=	-	=	-	<	0.3±0.0	=	0.4±0.0	>	-	<	-	-	-	=	-	=	-	=	-	=
16:1/18:4	0.1±0.0	<	0.2±0.0	=	0.2±0.1	=	0.2±0.1	=	0.2±0.0	=	0.2±0.1	=	0.2±0.0	<	0.3±0.1	=	0.4±0.1	=	0.4±0.2	=	0.5±0.2	>	+	<	0.1±0.0	0.1±0.0	0.1±0.0	=	0.1±0.0	=	0.2±0.1	=	0.1±0.1	=
20:5/14:0	0.1±0.0	=	+	=	0.1±0.0	=	0.1±0.0	=	+	=	0.1±0.0	=	+	=	+	=	0.1±0.0	=	+	=	+	=	+	=	0.1±0.0	0.1±0.0	+	=	0.2±0.0	=	0.1±0.0	=	0.1±0.0	=
16:1/18:3	0.1±0.1	=	0.2±0.1	<	0.3±0.0	<	0.7±0.3	>	0.2±0.0	>	0.1±0.1	<	0.3±0.2	=	0.5±0.1	=	0.6±0.0	=	0.8±0.1	=	0.7±0.2	>	0.3±0.1	<	0.2±0.0	0.1±0.0	0.1±0.0	=	0.3±0.1	=	0.2±0.1	=	0.2±0.1	=
18:1/16:3	+	=	+	=	+	=	+	=	+	<	0.1±0.0	=	+	=	+	=	+	=	+	=	+	=	0.1±0.0	>	0.1±0.0	0.1±0.0	-	=	-	=	0.1±0.0	=	0.1±0.0	=
20:4/14:0	-	=	-	=	-	<	+	>	-	<	0.2±0.0	>	-	=	-	=	-	<	+	=	+	<	0.3±0.0	>	-	-	-	=	0.1±0.0	=	-	=	-	=
16:0/18:4	3.0±0.2	<	6.3±1.3	<	11.2±1.5	=	9.5±4.4	=	6.1±0.3	>	1.3±0.6	<	2.3±0.1	<	3.7±1.2	=	3.6±0.8	>	1.9±0.9	=	1.9±0.4	>	0.6±0.3	<	2.4±0.1	1.4±0.4	4.6±0.2	>	0.8±0.5	=	2.0±0.2	=	0.6±0.1	=
16:1/18:2	+	>	+	=	+	=	0.1±0.0	=	1.6±2.7	=	+	=	+	=	+	=	+	<	0.1±0.0	=	0.1±0.1	>	-	<	3.1±4.4	-	+	=	+	=	+	=	0.1±0.0	=
14:0/20:3	+	>	-	=	-	<	+	>	-	=	-	<	-	=	-	<	+	=	+	>	-	=	-	<	-	-	-	=	-	=	+	=	0.1±0.0	=
16:0/18:3	4.9±1.1	>	2.7±0.6	<	3.9±0.2	<	6.0±0.5	=	5.0±0.9	=	4.5±0.4	<	5.3±0.5	=	4.7±0.5	<	6.0±0.6	>	4.1±0.6	=	4.3±0.4	=	4.6±1.0	=	6.7±0.6	6.4±1.3	5.0±0.4	=	3.7±0.6	=	6.6±0.0	>	5.0±0.7	=
16:1/18:1	-	=	-	<	+	=	+	>	-	=	-	<	+	>	-	<	+	=	+	=	+	>	-	<	0.1±0.1	-	+	=	-	=	+	=	-	=
16:0/18:2	0.3±0.0	>	0.1±0.0	=	0.3±0.1	<	0.6±0.1	=	0.7±0.2	=	0.8±0.3	=	0.3±0.2	=	0.4±0.1	=	0.4±0.0	<	0.6±0.1	=	0.6±0.1	=	0.7±0.3	=	0.6±0.1	1.1±0.4	0.2±0.1	=	1.2±0.1	=	0.5±0.0	=	0.8±0.2	=
16:0/18:1	0.1±0.1	=	0.2±0.1	=	0.1±0.0	<	0.3±0.1	=	0.2±0.0	=	0.4±0.3	=	0.3±0.0	>	0.2±0.0	<	0.3±0.1	=	0.5±0.2	>	0.3±0.0	=	0.5±0.2	=	1.2±0.3	1.3±0.5	0.1±0.0	=	0.4±0.3	=	0.5±0.0	>	0.2±0.3	=
18:5/18:4	+	=	0.1±0.1	=	0.2±0.1	=	0.1±0.1	=	0.1±0.0	>	-	<	+	<	0.4±0.2	>	0.1±0.1	=	0.1±0.1	=	0.1±0.1	=	0.2±0.1	=	0.1±0.1	0.1±0.0	+	=	0.1±0.0	=	0.1±0.0	=	+	=

20:5/16:4	0.1±0.0	=	+	=	+	=	+	>	-	<	+	=	-	<	0.1±0.0	=	0.1±0.0	=	0.1±0.1	=	+	>	-	=	-	0.1±0.0	-	=	-	=	+	=	-	=
18:4/18:4	2.3±0.5	=	2.4±0.3	=	2.5±0.3	=	2.0±0.3	>	1.3±0.1	=	1.1±0.5	<	2.8±0.5	<	4.0±0.5	=	4.3±0.5	=	3.5±0.9	=	2.6±0.1	>	1.9±0.3	<	3.5±0.3	2.7±0.2	1.7±0.1	=	0.9±0.5	=	3.2±0.3	=	2.6±1.0	=
20:5/16:3	+	=	+	=	+	>	-	<	+	=	+	>	+	=	+	>	+	=	+	<	+	>	-	<	+	+	-	=	-	=	-	=	+	=
18:3/18:4	1.1±0.4	=	1.1±0.3	=	1.1±0.7	=	1.0±0.5	=	0.8±0.4	=	1.0±0.6	=	1.7±0.7	=	3.2±1.2	=	4.3±0.7	=	4.4±1.3	=	3.3±0.4	>	1.9±1.0	<	3.5±1.5	2.0±2.3	0.8±0.3	=	0.3±0.2	=	5.3±0.6	>	4.0±1.5	=
20:5/16:2	0.3±0.2	>	-	<	0.4±0.1	=	0.4±0.2	=	0.1±0.1	=	0.5±0.4	=	1.2±0.5	>	-	=	-	<	0.2±0.2	=	0.1±0.0	<	2.6±0.4	>	2.1±0.0	2.9±0.5	0.4±0.1	=	0.9±0.4	=	-	=	0.5±0.0	=
16:3/20:4	0.1±0.0	=	0.1±0.1	=	0.1±0.0	=	0.1±0.1	=	0.1±0.1	=	0.2±0.2	=	0.1±0.0	=	0.1±0.1	=	0.1±0.0	=	0.1±0.1	=	0.1±0.0	=	0.1±0.0	=	0.3±0.0	0.1±0.0	0.2±0.0	=	-	=	0.2±0.1	=	0.1±0.1	=
18:3/18:3	0.6±0.2	<	1.3±0.5	=	1.8±0.4	=	1.8±1.2	=	1.2±0.2	=	1.1±0.6	=	2.0±0.4	<	4.0±1.0	<	5.9±0.4	=	5.1±1.1	=	5.2±1.0	=	4.9±1.1	=	2.6±0.1	3.1±0.7	0.7±0.2	=	1.0±0.4	=	2.8±0.6	=	3.8±1.7	=
18:3/18:3	+	<	0.1±0.0	=	0.2±0.0	>	-	<	0.1±0.0	=	0.1±0.1	>	0.1±0.0	=	0.2±0.1	=	0.2±0.1	>	-	<	0.1±0.0	=	0.3±0.3	>	0.1±0.0	0.1±0.0	0.1±0.0	=	0.1±0.0	=	0.1±0.1	=	0.1±0.1	=
20:5/16:1	0.3±0.2	=	0.1±0.1	=	0.1±0.0	=	0.2±0.1	=	0.1±0.0	<	0.4±0.1	>	0.1±0.0	=	0.1±0.1	=	0.2±0.0	=	0.3±0.1	>	+	=	0.3±0.2	=	0.1±0.1	0.3±0.1	0.1±0.0	=	0.2±0.0	=	0.3±0.1	=	0.3±0.0	=
18:5/18:1	+	=	+	=	+	=	0.1±0.1	=	0.1±0.0	=	0.1±0.0	=	+	=	+	<	0.1±0.0	<	0.2±0.0	>	+	<	0.3±0.0	=	0.1±0.2	0.1±0.1	+	=	-	=	+	=	+	=
18:3/18:2	0.1±0.1	=	0.2±0.1	=	0.2±0.1	=	0.6±0.3	=	0.5±0.1	=	0.7±0.4	=	0.3±0.1	=	0.4±0.2	<	0.7±0.1	<	1.6±0.6	=	1.1±0.4	=	0.8±0.8	=	0.4±0.1	0.8±0.4	0.1±0.0	=	0.5±0.0	=	0.4±0.1	=	0.8±0.3	=
20:4/16:1	+	=	0.1±0.0	=	+	=	+	=	+	<	0.2±0.1	>	0.1±0.1	=	0.1±0.0	=	0.1±0.0	=	+	=	0.1±0.0	=	0.1±0.1	=	+	0.1±0.0	+	=	0.2±0.0	=	+	=	0.1±0.0	=
20:5/16:0	0.3±0.2	=	0.1±0.1	=	0.2±0.1	=	0.2±0.1	=	0.2±0.1	<	0.7±0.3	>	0.3±0.1	=	0.4±0.1	=	0.3±0.1	=	0.3±0.1	=	0.5±0.1	=	0.4±0.1	=	0.7±0.1	0.4±0.3	0.3±0.0	=	0.6±0.2	=	0.5±0.0	=	0.5±0.1	=
18:2/18:2	0.1±0.0	=	0.1±0.0	=	0.1±0.0	<	0.3±0.0	=	0.2±0.2	=	0.2±0.1	=	0.1±0.0	<	0.1±0.0	<	0.2±0.0	<	0.6±0.1	=	0.4±0.2	=	0.2±0.1	<	0.1±0.1	0.3±0.3	0.1±0.0	=	0.2±0.2	=	0.2±0.0	>	0.2±0.0	=
18:1/18:3	0.1±0.0	=	0.1±0.0	=	0.1±0.0	=	0.1±0.0	=	0.1±0.1	=	+	=	0.1±0.1	<	0.2±0.0	=	0.2±0.1	=	0.2±0.1	=	0.2±0.1	>	0.1±0.0	=	0.2±0.1	0.2±0.1	0.1±0.0	=	0.3±0.0	=	0.1±0.0	=	0.1±0.0	=
16:0/20:4	+	=	+	=	+	=	0.1±0.0	=	+	=	0.1±0.1	=	0.1±0.0	=	+	=	+	=	+	=	+	<	0.2±0.1	>	0.2±0.0	0.4±0.3	+	=	-	=	0.1±0.0	=	0.2±0.0	=
20:4/16:0	0.1±0.0	>	-	=	-	<	+	=	+	<	0.3±0.2	>	+	=	-	=	+	=	+	=	+	<	0.1±0.1	>	+	0.2±0.0	+	=	0.4±0.2	=	+	=	0.2±0.0	=
18:0/18:4	0.5±0.1	=	0.7±0.2	=	0.5±0.0	=	0.6±0.3	=	0.5±0.1	>	0.2±0.0	<	0.4±0.1	=	0.6±0.2	=	0.5±0.1	>	0.2±0.1	=	0.2±0.1	=	0.2±0.1	=	0.4±0.1	0.3±0.1	0.5±0.2	=	0.1±0.0	=	0.3±0.1	=	0.1±0.0	=
18:2/18:1	+	=	-	<	+	=	0.1±0.1	=	+	=	0.1±0.0	=	-	=	-	=	+	<	0.1±0.0	>	0.1±0.0	=	+	<	0.1±0.0	0.1±0.1	+	=	0.1±0.0	=	0.1±0.0	=	+	=
18:0/18:3	0.3±0.1	>	0.1±0.0	=	0.1±0.0	<	0.2±0.1	=	0.2±0.1	<	0.5±0.2	>	0.4±0.2	=	0.3±0.1	=	0.3±0.1	=	0.2±0.0	>	0.2±0.0	<	0.8±0.4	>	0.5±0.1	0.3±0.0	0.4±0.0	=	0.5±0.1	=	0.4±0.2	=	0.5±0.0	=
18:1/18:1	+	>	-	=	-	<	+	=	+	=	+	<	+	=	-	=	+	=	+	=	+	=	0.1±0.1	=	+	0.1±0.0	+	=	-	=	+	=	0.1±0.0	=
18:0/18:2	+	>	-	<	+	=	+	=	+	=	0.1±0.0	>	+	=	-	=	-	=	-	<	+	>	-	=	+	+	+	=	0.2±0.0	=	+	=	+	=
18:0/18:1	0.1±0.0	>	-	<	+	=	+	>	+	=	0.1±0.1	=	+	>	-	=	+	=	+	=	+	=	+	=	0.1±0.1	0.1±0.0	+	=	0.1±0.0	=	+	=	0.1±0.0	=
18:2/19:2	-	=	-	=	-	=	-	=	-	=	-	=	+	>	-	<	+	<	0.1±0.0	=	0.1±0.0	=	0.1±0.0	=	0.2±0.1	0.5±0.4	-	=	-	=	0.1±0.1	=	0.1±0.2	=
18:3/19:1	-	=	-	=	-	=	-	=	-	=	-	=	-	=	-	=	-	<	+	=	+	>	-	<	-	-	-	=	-	=	-	=	+	=
20:5/18:5	+	=	0.1±0.0	=	+	>	-	<	+	>	-	=	+	=	+	>	-	<	+	=	+	=	+	=	+	-	+	=	-	=	+	=	+	=
20:5/18:4	67.2±3.5	=	73.2±4.6	>	61.1±1.9	=	55.5±4.7	=	61.3±2.3	>	50.7±3.6	=	62.0±1.1	=	63.6±4.2	=	58.2±2.3	=	60.4±0.6	=	62.7±5.0	>	49.0±2.7	<	57.0±2.8	56.7±3.7	65.2±1.9	=	45.8±6.0	=	56.2±2.2	=	50.5±5.4	=
20:5/18:3	1.6±0.5	>	1.0±0.2	>	0.7±0.1	<	1.5±0.2	=	1.1±0.3	<	2.5±0.4	>	1.4±0.5	=	1.0±0.3	=	1.1±0.2	<	1.4±0.1	=	1.6±0.2	<	7.4±4.6	>	1.6±0.3	4.7±1.0	1.2±0.2	=	1.4±0.1	<	2.0±0.1	=	4.9±0.3	=
20:4/18:4	8.6±0.8	>	4.6±0.2	<	6.3±1.0	<	8.8±0.8	=	9.6±0.9	<	23.4±1.4	>	8.2±0.8	>	4.1±0.5	=	4.6±0.8	<	6.7±1.2	=	7.2±1.1	=	12.1±4.5	>	4.6±0.3	7.2±3.3	8.8±0.4	=	30.0±6.4	=	6.3±0.0	=	14.5±7.5	=
20:4/18:3	0.5±0.1	>	0.2±0.1	=	0.1±0.1	<	0.5±0.1	>	0.3±0.1	<	1.7±0.9	>	0.6±0.1	>	0.2±0.0	>	0.1±0.1	<	0.5±0.1	>	0.3±0.0	<	3.5±1.3	>	0.4±0.1	1.1±0.6	0.5±0.0	=	3.0±0.3	=	0.5±0.3	=	2.5±1.1	=
20:5/18:2	0.2±0.1	=	+	=	0.1±0.1	=	0.1±0.0	=	0.1±0.0	=	0.8±0.5	>	-	<	+	=	0.1±0.1	=	0.1±0.0	=	0.3±0.3	=	0.1±0.0	=	0.1±0.0	0.4±0.0	+	=	0.5±0.4	=	0.1±0.1	=	0.2±0.1	=
18:3/20:4	0.1±0.1	=	+	=	0.1±0.1	=	+	=	0.1±0.1	=	0.5±0.6	=	-	<	+	=	+	=	+	=	+	<	+	=	0.1±0.0	0.1±0.1	+	=	0.6±0.3	=	+	=	+	=
20:4/18:2	0.1±0.0	>	+	=	+	=	+	=	+	=	0.1±0.1	=	+	=	+	=	+	=	+	=	+	<	0.2±0.1	>	0.1±0.0	0.1±0.1	+	=	-	=	+	=	+	=
20:5/18:1	+	>	-	<	+	=	+	=	+	<	0.2±0.1	>	+	=	-	<	+	=	+	=	+	=	0.2±0.3	=	+	0.1±0.1	+	=	0.4±0.2	=	0.1±0.0	=	0.1±0.0	=
SFA/SFA	0.1±0.0	>	+	=	+	=	+	=	+	=	0.1±0.1	=	+	=	+	=	+	=	+	=	+	=	+	=	+	+	0.1±0.0	=	0.1±0.0	=	0.1±0.1	=	0.1±0.0	=
SFA/MUFA	0.4±0.2	=	0.3±0.2	=	0.3±0.1	<	0.5±0.2	=	0.4±0.0	<	1.1±0.5	=	0.7±0.2	=	0.5±0.1	=	0.5±0.1	=	0.9±0.3	=	0.6±0.1	=	0.7±0.4	=	2.1±0.3	1.8±0.6	0.5±0.0	=	0.8±0.3	=	1.6±0.0	>	0.6±0.4	=
MUFA/SFA	-	=	-	=	-	=	-	=	-	=	-	=	-	=	-	=	-	=	-	=	-	=	-	=	-	-	-	=	-	=	-	=	-	=
SFA/PUFA	15.7±3.1	=	14.3±3.9	<	23.9±2.2	=	24.5±6.6	=	20.1±1.6	>	12.8±0.7	<	17.8±1.0	=	15.9±1.8	=	17.2±1.7	>	10.8±2.5	=	11.3±1.5	=	12.9±3.3	=	18.7±1.0	14.5±0.9	18.9±1.2	=	11.2±0.5	=	18.2±1.1	=	12.9±1.9	=
PUFA/SFA	0.4±0.2	>	0.1±0.1	<	0.3±0.1	=	0.3±0.1	=	0.3±0.1	<	1.1±0.2	>	0.4±0.1	=	0.5±0.1	=	0.4±0.0	=	0.4±0.1	<	0.5±0.1	=	0.6±0.2	>	0.8±0.1	0.4±0.3	0.3±0.0	=	1.2±0.1	=	0.6±0.1	=	0.6±0.2	=
MUFA/MUFA	+	=	+	=	+	<	+	>	+	=	+	<	0.1±0.1	=	+	=	+	=	+	=	+	<	0.1±0.1	=	0.2±0.1	0.2±0.1	+	=	+	=	0.3±0.0	>	0.1±0.1	=
MUFA/PUFA	0.4±0.2	=	0.5±0.1	=	0.6±0.1	<	1.1±0.2	=	2.1±2.6	=	0.4±0.1	<	0.7±0.2	<	1.2±0.2	<	1.4±0.0	=	2.3±1.1	=	1.5±0.5	>	0.5±0.1	<	2.7±3.8	0.5±0.3	0.4±0.0	=	0.7±0.2	=	0.9±0.3	=	0.5±0.1	=
PUFA/ MUFA	+	=	+	=	+	=	0.1±0.1	=	+	=	0.2±0.2	=	+	<	+	=	0.1±0.0	<	0.2±0.1	=	0.1±0.0	=	0.2±0.2	=	0.1±0.1	0.2±0.0	+	=	0.5±0.2	=	0.1±0.0	=	0.1±0.0	=
PUFA/PUFA	83.1±3.6	=	84.8±4.1	>	74.9±2.1	=	73.5±6.2	=	77.1±2.4	<	84.5±1.3	>	80.4±1.5	=	81.9±2.0	=	80.4±1.8	<	85.5±3.2	=	86.0±2.0	=	85.0±3.4	=	75.4±2.7	82.3±1.4	79.8±1.1	=	85.6±0.3	=	78.3±1.3	=	85.3±1.7	=

SQDG	The lower part of the blades of infected *U. pinnatifida* samples												The upper intact part of the blades of infected *U. pinnatifida* samples												The upper part of the blades of infected *U. pinnatifida* samples with endophyte		The lower part of the blades of uninfected *U. pinnatifida* samples				The upper part of the blades of uninfected *U. pinnatifida* samples			
	Nov.		Dec.		Jan.		Feb.		Apr.		Jun.	T	Nov.		Dec.		Jan.		Feb.		Apr.		Jun.	T	Nov.	Jun.	Nov.	S	Jun.	S	Nov.	S	Jun.	S
14:0/14:0	0.7±0.2	>	0.2±0.1	=	0.1±0.0	<	0.3±0.1	=	0.2±0.2	<	0.5±0.1	=	1.1±0.6	=	0.8±0.3	>	0.2±0.1	<	0.4±0.2	>	0.1±0.0	<	0.9±0.5	=	1.5±0.8	1.2±0.2	0.7±0.2	=	0.9±0.2	=	3.7±0.3	>	0.7±0.2	=
14:0/16:2	0.1±0.0	=	0.3±0.4	=	0.2±0.1	=	0.1±0.1	=	0.1±0.1	=	0.1±0.1	=	0.1±0.1	=	0.5±0.4	=	0.3±0.1	=	0.2±0.0	=	0.2±0.1	=	0.2±0.0	=	0.1±0.0	0.2±0.2	0.2±0.1	=	0.2±0.0	=	0.3±0.0	>	+	=
14:0/16:1	0.5±0.2	=	0.2±0.0	=	0.4±0.2	=	0.5±0.1	>	0.2±0.1	<	0.5±0.1	=	0.9±0.3	=	0.9±0.2	>	0.5±0.2	=	0.5±0.1	>	0.4±0.1	=	0.5±0.3	=	1.2±0.8	0.5±0.2	0.6±0.1	=	0.8±0.3	=	1.2±0.4	=	0.4±0.2	=
14:0/16:0	7.9±1.4	>	0.7±0.4	=	1.0±0.0	=	0.7±0.4	=	1.0±0.2	<	7.5±2.1	>	5.8±1.1	>	1.2±0.2	=	1.2±0.3	>	0.7±0.3	=	0.8±0.2	<	4.6±2.5	>	6.5±0.4	6.0±1.7	7.7±1.2	=	7.4±0.6	=	5.5±0.3	=	5.4±0.3	=
15:1/16:1	-	=	-	=	-	<	+	>	-	=	-	<	+	=	-	=	+	=	+	=	+	>	-	<	0.1±0.0	+	+	=	-	=	+	=	-	=
16:1/15:0	+	=	-	<	0.1±0.0	=	+	=	0.1±0.1	>	-	<	+	>	-	<	0.1±0.0	=	0.1±0.0	=	0.1±0.0	=	0.2±0.0	=	+	-	+	=	-	=	+	=	0.1±0.0	=
15:0/16:0	+	>	-	=	-	=	-	=	-	<	0.2±0.0	>	0.1±0.0	>	-	=	+	=	+	=	+	<	0.3±0.0	>	0.1±0.0	0.1±0.0	+	=	0.1±0.0	=	0.1±0.0	=	0.1±0.1	=
14:0/18:4	0.4±0.1	=	0.4±0.2	=	0.3±0.2	=	0.1±0.0	=	0.1±0.0	=	0.1±0.0	=	0.3±0.2	<	0.8±0.3	>	0.4±0.1	=	0.3±0.1	=	0.2±0.1	=	0.1±0.1	=	0.2±0.1	0.2±0.0	0.2±0.0	=	0.1±0.0	=	1.1±0.1	>	0.1±0.0	=
14:0/18:3	6.3±1.2	=	5.2±0.8	=	4.9±0.5	>	3.3±0.1	=	3.3±0.4	=	4.0±1.4	=	5.2±1.5	=	6.0±1.0	>	4.7±0.3	>	2.3±0.5	=	2.4±0.4	=	3.4±0.9	=	3.5±0.3	2.2±0.3	6.4±1.0	=	4.2±0.5	=	3.9±0.7	=	3.9±0.3	=
14:0/18:2	3.7±1.2	>	1.4±0.7	=	2.1±0.8	<	4.4±1.6	=	4.3±0.8	<	6.0±1.2	=	4.4±0.2	>	2.3±0.8	=	2.7±0.9	=	2.8±0.8	=	3.3±0.4	<	6.7±1.3	>	3.0±0.5	3.9±1.7	2.8±0.1	=	4.7±2.0	=	2.9±0.1	<	7.0±3.0	=
14:0/18:1	2.0±0.3	>	1.1±0.7	=	1.8±0.8	=	2.1±0.5	=	1.4±0.6	<	2.3±0.5	=	2.1±0.5	=	1.8±0.9	=	1.6±0.7	<	2.6±0.2	>	1.9±0.5	=	2.4±1.4	=	2.0±0.8	2.1±0.8	2.5±0.0	=	1.8±0.5	=	2.7±0.1	=	2.2±0.6	=
16:1/16:0	0.9±0.0	=	0.8±0.0	=	0.8±0.0	=	0.5±0.3	=	1.1±0.7	=	0.6±0.0	=	1.0±0.0	=	0.6±0.0	=	1.2±0.0	>	0.4±0.1	<	1.3±0.5	=	1.3±0.0	>	0.9±0.0	1.4±0.5	-	=	-	=	-	=	-	=
16:0/16:0	2.0±0.4	>	0.9±0.1	>	0.5±0.0	=	0.4±0.1	<	0.7±0.2	<	4.0±0.5	>	1.9±1.7	=	0.6±0.1	<	0.9±0.2	=	0.8±0.4	=	1.4±0.4	<	7.4±0.8	>	3.4±0.3	6.0±0.8	1.7±0.2	=	3.8±0.8	=	3.6±0.5	=	6.3±1.5	=
15:0/18:3	0.2±0.1	>	-	<	0.1±0.1	=	0.1±0.1	=	0.2±0.1	=	0.2±0.1	=	0.2±0.1	>	-	<	0.2±0.1	=	0.1±0.1	=	0.2±0.1	=	0.2±0.0	=	0.1±0.0	0.5±0.3	0.2±0.1	=	0.2±0.0	=	0.2±0.0	=	0.3±0.3	=
15:0/18:2	-	=	-	=	-	=	-	=	-	=	-	=	+	=	-	=	-	=	-	=	-	=	-	=	-	-	-	=	-	=	-	=	-	=
15:0/18:1	0.3±0.2	=	0.4±0.2	=	0.4±0.2	=	0.5±0.3	=	0.3±0.1	=	0.5±0.3	=	0.6±0.1	=	0.6±0.2	=	0.6±0.1	=	0.6±0.1	=	0.4±0.2	=	0.5±0.3	=	0.5±0.1	0.6±0.2	0.4±0.2	=	0.7±0.0	=	0.7±0.1	=	0.5±0.3	=
16:0/17:0	+	=	-	=	-	<	0.1±0.0	>	-	<	0.1±0.1	=	+	>	-	<	+	>	-	<	+	=	0.1±0.1	=	-	+	-	=	+	=	0.1±0.1	=	0.1±0.0	=
20:5/14:0	+	<	0.1±0.0	=	0.1±0.0	=	0.1±0.1	=	0.1±0.0	>	-	<	0.1±0.0	=	0.1±0.0	=	0.1±0.1	=	0.1±0.0	=	0.1±0.0	>	-	<	+	0.1±0.0	+	=	-	=	+	=	+	=
14:0/20:5	-	<	0.1±0.0	>	-	<	+	=	+	=	-	<	-	<	+	=	+	=	0.1±0.0	>	-	=	-	<	0.1±0.0	0.1±0.0	-	=	-	=	+	=	0.1±0.0	=
16:1/18:3	0.1±0.1	=	0.5±0.4	=	0.3±0.1	=	0.2±0.1	=	0.2±0.1	=	0.2±0.1	=	0.1±0.1	<	0.6±0.2	>	0.2±0.1	>	+	<	0.2±0.1	>	+	=	0.1±0.0	0.2±0.2	0.2±0.1	=	0.1±0.0	=	0.1±0.1	=	0.1±0.0	=
14:0/20:4	-	<	0.7±0.3	>	0.1±0.0	=	0.2±0.2	=	+	=	+	=	0.1±0.0	=	0.1±0.1	=	0.1±0.1	=	0.1±0.0	=	+	=	0.2±0.2	=	0.3±0.3	0.3±0.0	0.1±0.0	=	-	=	0.1±0.0	=	0.2±0.1	=
18:3/16:0+<br>16:0/18:3	5.9±1.3	<	8.3±0.5	=	7.8±1.2	=	6.6±1.0	>	3.8±1.1	=	6.1±1.8	=	6.4±1.8	<	10.6±1.4	=	10.7±0.3	>	7.4±1.0	=	8.8±0.8	=	10.2±1.2	>	6.3±0.9	11.5±1.8	5.7±0.3	=	8.0±1.8	=	6.6±0.6	=	12.5±4.2	=
18:2/16:0	3.4±0.3	<	9.5±0.5	=	11.3±2.6	=	13.7±2.4	>	9.3±0.5	>	7.2±1.6	<	3.7±0.7	<	6.2±0.5	=	6.8±0.8	<	11.7±0.5	>	9.7±0.3	=	11.5±3.4	=	4.1±0.4	12.6±3.5	3.0±0.5	=	6.6±1.2	=	3.9±0.6	=	11.8±1.8	=
18:1/16:0	60.2±3.7	=	58.9±2.6	=	59.9±1.5	=	58.5±2.9	<	67.5±1.4	>	55.8±5.1	=	60.3±2.1	>	56.0±1.9	=	57.9±1.7	=	61.2±3.2	=	61.1±1.0	>	45.4±2.3	<	58.1±1.3	45.2±2.3	63.0±3.6	=	56.9±7.9	=	56.9±0.9	=	41.4±4.5	=
18:1/16:0	2.5±0.3	=	3.4±2.6	=	4.9±0.3	=	5.0±0.8	>	3.6±0.4	>	1.6±0.4	<	1.2±0.5	<	2.3±0.8	=	2.9±0.7	=	3.2±0.3	>	2.7±0.2	>	0.7±0.6	<	1.8±0.5	1.0±0.5	1.5±0.1	<	1.3±0.0	=	1.5±0.3	=	1.4±0.7	=
18:0/16:0	0.1±0.0	=	0.2±0.0	>	0.1±0.0	=	0.1±0.0	=	0.1±0.0	<	0.4±0.1	>	0.1±0.0	=	0.1±0.0	=	0.1±0.0	=	0.1±0.0	=	0.1±0.0	<	0.4±0.2	>	0.3±0.2	0.3±0.1	0.1±0.0	=	0.4±0.2	=	0.2±0.1	=	0.5±0.3	=
18:4/18:4	0.4±0.3	=	1.4±1.6	=	0.3±0.1	=	0.4±0.2	>	0.1±0.1	=	0.2±0.1	<	0.6±0.2	=	0.4±0.2	=	0.3±0.0	=	0.4±0.5	=	0.6±0.7	=	0.5±0.3	=	2.5±1.3	0.4±0.2	0.2±0.1	=	0.1±0.0	=	0.6±0.0	=	0.4±0.1	=
18:3/18:4	0.4±0.2	=	0.9±0.5	=	0.5±0.1	>	0.1±0.1	=	0.2±0.0	=	0.2±0.1	=	0.9±0.1	=	1.4±0.5	=	1.1±0.2	>	0.5±0.2	=	0.5±0.1	=	0.5±0.2	=	1.3±0.6	0.8±0.4	0.4±0.0	=	0.4±0.5	=	1.0±0.3	=	0.6±0.3	=
18:3/18:3	0.8±0.4	=	1.5±0.7	=	1.0±0.4	=	0.6±0.3	=	0.4±0.2	=	0.4±0.2	=	1.1±0.4	=	1.7±0.6	=	1.8±0.2	>	0.8±0.1	=	0.8±0.1	=	0.6±0.4	=	0.9±0.6	0.6±0.3	0.8±0.2	=	0.4±0.0	=	0.8±0.1	=	0.8±0.1	=
18:3/18:2	0.3±0.1	=	0.7±0.5	=	0.2±0.1	<	0.3±0.0	=	0.2±0.1	=	0.2±0.2	=	0.2±0.1	<	0.5±0.2	=	0.4±0.1	=	0.4±0.1	=	0.3±0.1	=	0.3±0.2	=	0.2±0.1	0.2±0.1	0.2±0.0	=	0.2±0.0	=	0.2±0.0	=	0.7±0.3	=
20:5/16:0	0.1±0.1	<	0.5±0.3	>	0.1±0.0	=	0.1±0.0	=	0.2±0.1	=	0.1±0.1	=	0.2±0.1	=	0.3±0.1	=	0.2±0.1	=	0.4±0.3	=	0.1±0.1	=	0.2±0.2	=	0.2±0.0	0.2±0.2	0.1±0.0	=	0.4±0.4	=	0.2±0.1	=	0.2±0.0	=
18:2/18:2	0.2±0.0	>	+	>	-	<	0.1±0.0	=	0.1±0.1	=	0.2±0.1	>	0.1±0.0	=	0.1±0.0	=	0.1±0.0	=	0.1±0.1	=	0.1±0.1	=	0.2±0.2	=	0.1±0.1	0.2±0.1	+	<	0.2±0.0	=	0.1±0.0	=	0.2±0.0	=
20:4/16:0	-	<	0.2±0.0	>	0.1±0.0	<	0.2±0.1	>	0.1±0.0	=	+	<	-	<	0.1±0.0	=	+	=	+	=	0.1±0.1	<	0.3±0.0	>	+	0.1±0.1	+	=	0.2±0.0	=	-	=	0.1±0.0	=
16:0/20:4	0.2±0.0	=	0.3±0.0	=	0.1±0.1	>	-	<	0.1±0.0	=	0.1±0.1	>	0.1±0.0	=	0.1±0.0	<	0.2±0.1	=	0.2±0.0	=	0.2±0.0	=	0.4±0.0	=	+	0.2±0.2	+	=	0.1±0.0	=	-	=	0.1±0.2	=

18:2/18:1	+	=	-	=	0.1±0.1	=	0.1±0.0	<	0.6±0.4	=	0.1±0.1	=	+	>	-	<	0.1±0.1	=	+	<	0.3±0.2	>	+	=	+	0.3±0.1	-	=	0.3±0.0	=	0.1±0.1	=	0.1±0.0	=
20:3/16:0	0.3±0.0	=	0.3±0.3	=	0.1±0.0	=	0.1±0.2	=	0.4±0.3	=	0.3±0.1	=	0.4±0.3	=	0.4±0.2	=	0.5±0.1	=	0.5±0.2	=	0.8±0.8	=	0.4±0.1	=	0.3±0.2	0.4±0.1	0.2±0.2	=	0.3±0.0	=	0.2±0.1	=	0.3±0.1	=
18:0/18:3	+	<	0.6±0.4	=	0.1±0.0	=	0.1±0.0	=	+	=	+	=	-	<	0.1±0.0	>	-	=	0.2±0.2	=	0.1±0.2	=	-	=	0.1±0.1	-	+	=	-	=	0.1±0.0	=	-	=
16:0/20:0	0.1±0.1	=	0.1±0.0	=	+	=	0.1±0.0	=	0.1±0.0	<	0.2±0.1	>	0.2±0.0	>	+	=	+	=	+	=	+	<	0.6±0.1	>	0.4±0.1	0.4±0.2	0.1±0.0	=	0.5±0.1	=	0.2±0.1	=	0.5±0.3	=
20:5/18:4	0.3±0.2	<	1.8±1.2	=	0.9±0.1	=	0.7±0.4	=	0.5±0.2	=	0.3±0.2	=	1.1±0.2	<	2.1±0.5	=	1.8±0.3	>	1.2±0.3	>	0.7±0.3	>	0.3±0.1	<	1.3±0.3	0.6±0.3	0.6±0.1	=	0.2±0.0	=	0.7±0.1	=	0.9±0.1	=
20:5/18:3	0.2±0.2	=	0.3±0.2	=	0.1±0.1	=	0.2±0.1	=	0.1±0.0	<	0.2±0.1	=	0.1±0.1	=	0.4±0.2	=	0.4±0.0	=	0.2±0.2	=	0.1±0.1	=	0.1±0.1	=	0.1±0.1	0.2±0.0	0.1±0.0	=	0.1±0.0	=	0.1±0.0	=	0.1±0.0	=
20:4/18:4	0.2±0.2	=	0.3±0.2	=	0.1±0.1	=	0.1±0.0	=	0.1±0.1	=	0.2±0.1	=	0.1±0.1	=	0.4±0.2	=	0.4±0.1	>	0.1±0.1	=	0.2±0.0	=	0.1±0.1	=	0.1±0.1	0.2±0.0	0.1±0.0	=	0.1±0.0	=	0.1±0.0	=	0.1±0.0	=
20:4/18:3	-	=	-	=	-	<	0.1±0.0	>	-	=	-	<	-	=	-	=	-	<	+	>	-	<	0.1±0.0	=	0.3±0.0	+	-	=	-	=	0.1±0.0	=	-	=
20:3/18:4	-	<	0.2±0.2	=	+	>	-	<	+	>	-	=	+	=	+	=	+	=	+	=	0.1±0.0	=	0.1±0.0	=	+	0.1±0.0	+	=	-	=	+	=	0.1±0.0	=
20:4/18:3	0.1±0.1	=	-	=	-	<	0.1±0.1	=	0.1±0.0	>	+	<	0.1±0.0	=	-	=	0.1±0.1	>	-	<	0.1±0.0	=	+	>	+	-	-	=	+	=	+	=	+	=
20:5/20:5	-	=	-	=	-	<	+	>	-	=	-	<	-	=	-	=	-	<	+	>	-	=	-	<	-	-	-	=	-	=	-	=	-	=
20:4/20:4	-	=	-	=	-	<	+	>	-	=	-	<	-	=	-	=	-	<	+	>	-	=	-	<	+	-	+	=	-	=	-	=	-	=
SFA/SFA	10.8±1.1	>	1.7±0.3	=	1.7±0.0	=	1.5±0.5	=	2.0±0.1	<	12.7±1.7	>	9.1±1.7	>	2.8±0.3	=	2.5±0.3	=	2.0±0.4	=	2.4±0.2	<	14.2±4.0	>	11.9±0.6	14.0±2.0	10.3±0.8	=	13.0±1.3	=	13.5±0.4	>	13.7±1.1	=
SFA/MUFA	2.9±0.2	>	1.7±0.6	=	2.6±0.8	=	3.1±0.9	=	1.9±0.4	<	3.2±0.9	=	3.6±0.7	=	3.4±0.8	=	2.7±0.6	<	3.8±0.1	>	2.7±0.6	=	3.4±0.8	=	3.7±0.2	3.2±1.0	3.5±0.3	=	3.0±0.7	=	4.6±0.2	=	3.2±0.7	=
MUFA/SFA	-	=	-	=	-	=	-	=	-	=	-	=	-	=	-	=	-	=	-	=	-	=	-	=	-	-	-	=	-	=	-	=	-	=
SFA/PUFA	13.7±2.2	=	12.3±0.6	>	11.6±0.1	=	11.6±1.5	=	9.9±1.2	<	13.7±1.2	=	13.5±2.3	=	15.4±1.3	=	14.0±1.3	>	9.6±1.5	=	11.0±0.7	<	15.8±1.6	>	10.4±0.6	13.0±1.2	12.7±1.3	=	13.3±3.8	=	12.0±0.2	=	18.0±1.2	=
PUFA/SFA	6.8±0.5	<	14.4±0.7	=	15.4±2.1	=	17.4±2.0	>	11.8±0.6	=	10.7±2.6	<	7.5±0.4	<	12.4±0.7	=	13.0±1.0	<	16.3±0.6	=	15.1±1.2	=	17.4±4.1	=	7.6±0.7	19.1±4.4	6.2±0.8	=	11.3±1.7	=	7.6±0.6	=	18.5±4.2	=
MUFA/MUFA	+	=	+	=	+	=	+	=	+	=	+	=	+	=	+	=	+	=	+	=	+	=	+	=	+	+	+	=	+	=	+	=	+	=
MUFA/PUFA	0.1±0.1	=	0.5±0.4	=	0.3±0.1	=	0.1±0.1	=	0.1±0.1	=	0.2±0.1	=	0.1±0.1	<	0.6±0.2	>	0.2±0.1	>	+	<	0.2±0.1	>	+	=	+	0.1±0.1	0.2±0.1	=	0.1±0.1	=	0.1±0.1	=	0.1±0.1	=
PUFA/ MUFA	+	=	0.1±0.1	=	0.1±0.1	=	+	=	0.4±0.4	=	0.1±0.1	=	+	=	+	<	0.1±0.1	>	+	<	0.3±0.2	>	+	=	+	0.2±0.2	+	=	0.1±0.2	=	0.1±0.1	=	+	=
PUFA/PUFA	2.6±0.3	<	6.7±1.9	>	3.3±0.3	>	2.5±0.6	>	1.7±0.3	=	1.8±0.6	=	4.3±0.6	<	7.1±1.8	=	6.3±0.5	>	3.6±0.5	=	3.2±1.0	=	2.7±0.8	=	6.0±1.5	3.4±1.2	2.5±0.3	=	1.0±0.4	=	3.8±0.2	=	3.8±0.3	=

PG	The lower part of the blades of infected *U. pinnatifida* samples												The upper intact part of the blades of infected *U. pinnatifida* samples												The upper part of the blades of infected *U. pinnatifida* samples with endophyte		The lower part of the blades of uninfected *U. pinnatifida* samples				The upper part of the blades of uninfected *U. pinnatifida* samples			
	Nov.		Dec.		Jan.		Feb.		Apr.		Jun.	T	Nov.		Dec.		Jan.		Feb.		Apr.		Jun.	T	Nov.	Jun.	Nov.	S	Jun.	S	Nov.	S	Jun.	S
14:0/16:3	+	=	+	>	+	>	-	<	+	>	-	=	+	>	+	>	-	<	+	>	-	=	-	<	+	+	-	=	0.1±0.0	=	+	=	-	=
14:0/16:2	-	<	+	>	-	<	+	=	+	>	-	<	+	=	+	=	+	=	+	=	+	<	+	>	+	+	-	=	-	=	0.1±0.0	>	+	=
14:0/16:2	+	>	-	<	+	=	+	>	-	<	+	=	+	=	-	=	-	=	-	=	-	<	+	>	-	+	+	=	-	=	+	=	-	=
14:0/16:1	0.1±0.0	>	+	=	+	=	+	=	+	=	+	=	0.1±0.0	=	0.1±0.1	>	+	<	0.1±0.1	>	+	<	0.1±0.0	=	0.1±0.0	0.2±0.1	+	=	-	=	0.4±0.1	=	+	=
14:0/16:1	-	=	-	=	+	=	+	=	+	<	+	=	0.1±0.0	>	-	<	+	=	+	>	+	<	0.1±0.0	=	0.1±0.0	-	+	=	-	=	+	=	+	=
15:0/15:0	-	=	-	=	-	<	+	=	+	>	-	<	-	=	-	=	-	=	-	=	-	<	+	>	+	0.1±0.0	-	=	-	=	+	=	-	=
14:0/16:0	0.1±0.0	=	+	=	+	=	+	=	+	=	0.1±0.1	=	0.1±0.0	>	+	<	+	=	+	=	+	<	0.1±0.1	>	0.1±0.1	0.2±0.1	0.1±0.0	=	+	=	0.1±0.0	=	0.1±0.0	=
14:0/16:0	+	=	+	>	-	<	+	=	+	=	+	>	-	<	+	=	+	>	-	<	+	=	+	>	+	+	+	=	0.1±0.0	=	+	=	-	=
16:1/15:0	+	=	+	=	+	=	+	=	+	=	+	>	+	=	+	=	+	=	+	=	+	=	+	=	+	+	+	=	-	=	+	=	+	=
16:1/16:4	-	=	-	<	+	>	-	<	+	>	-	=	+	>	-	<	+	=	+	>	-	=	-	<	+	+	+	=	-	=	+	=	-	=
14:1/18:3	0.1±0.0	=	0.1±0.0	=	0.1±0.0	=	0.1±0.1	=	0.1±0.0	>	+	<	0.1±0.1	=	0.1±0.0	=	0.1±0.0	=	0.1±0.0	=	0.1±0.0	=	0.1±0.0	=	0.2±0.0	0.1±0.0	0.1±0.0	=	-	=	0.2±0.0	=	+	=
16:1/16:2	-	<	+	=	+	=	+	=	+	=	+	=	+	=	+	=	+	=	+	=	+	<	0.1±0.0	>	+	0.1±0.0	+	=	+	=	+	=	-	=
18:3/14:0	0.2±0.0	>	0.1±0.0	=	0.1±0.0	=	+	=	0.1±0.0	<	0.2±0.1	>	0.2±0.1	>	0.1±0.0	=	0.1±0.0	>	+	<	0.1±0.0	<	0.3±0.1	>	0.2±0.0	0.2±0.0	0.2±0.0	=	0.2±0.1	=	0.2±0.1	=	0.2±0.1	=
14:0/18:3	+	=	+	<	0.1±0.0	=	+	<	0.1±0.0	=	0.1±0.0	=	+	=	+	=	+	>	+	<	+	=	+	=	+	+	+	=	+	=	+	=	0.1±0.0	=

16:1/16:1	+	=	+	=	+	=	+	>	+	<	+	=	+	<	0.1±0.0	>	+	<	0.1±0.0	=	0.1±0.0	=	+	=	+	0.1±0.0	+	=	-	=	0.1±0.0	>	0.1±0.0	=
14:0/18:2	0.2±0.1	>	0.1±0.0	<	0.2±0.1	<	0.3±0.0	<	0.4±0.0	>	0.2±0.1	<	0.2±0.1	=	0.2±0.1	=	0.2±0.0	=	0.2±0.1	=	0.3±0.1	=	0.4±0.2	=	0.2±0.1	0.5±0.1	0.3±0.0	=	0.2±0.1	=	0.4±0.1	=	0.2±0.1	=
16:0/16:2	+	=	+	=	+	=	+	>	-	<	+	<	-	<	+	=	+	>	+	<	+	<	0.1±0.0	>	+	+	+	=	-	=	+	=	+	=
14:0/18:1+ 16:1/16:0+ 16:0/16:1	0.6±0.1	>	0.3±0.1	<	0.5±0.1	=	0.6±0.3	>	0.1±0.1	<	0.5±0.1	=	0.8±0.3	=	0.6±0.1	=	0.5±0.1	>	0.2±0.2	<	0.9±0.2	>	0.6±0.0	>	0.9±0.2	0.9±0.1	0.8±0.1	=	0.5±0.1	=	2.0±0.5	=	0.6±0.1	=
16:0/16:0	+	=	+	=	+	=	+	=	+	<	0.1±0.0	>	+	>	+	=	+	=	+	=	+	<	0.1±0.0	>	+	0.1±0.0	+	=	+	=	+	=	+	=
15:0/18:4	0.1±0.0	=	0.1±0.0	=	0.1±0.0	=	0.1±0.0	=	0.1±0.0	>	+	=	0.2±0.0	=	0.2±0.0	=	0.2±0.0	>	0.1±0.0	=	0.1±0.0	=	0.1±0.2	=	0.1±0.1	0.1±0.1	0.1±0.0	=	-	=	0.1±0.0	=	0.1±0.1	=
15:0/18:3	0.1±0.0	=	0.1±0.0	=	0.1±0.0	>	+	=	+	<	0.1±0.1	>	0.1±0.0	=	0.1±0.0	=	0.1±0.0	>	+	=	+	<	0.2±0.1	>	0.1±0.0	0.2±0.1	0.1±0.0	=	0.2±0.0	=	0.1±0.0	=	0.4±0.1	=
17:3/16:0	+	>	-	=	-	=	-	=	-	<	+	>	+	>	-	=	+	>	-	=	-	<	+	>	+	+	-	=	-	=	-	=	-	=
15:0/18:2	+	=	+	=	+	=	+	=	+	=	0.1±0.1	=	0.1±0.0	=	+	=	0.1±0.0	=	+	=	0.1±0.0	=	0.1±0.2	=	0.1±0.0	0.1±0.1	+	=	0.1±0.0	=	0.1±0.0	=	0.1±0.1	=
17:2/16:0	-	=	-	<	+	>	-	<	+	=	+	>	-	=	-	=	+	>	-	<	+	>	-	=	-	+	-	=	+	=	-	=	-	=
15:0/18:1	+	=	-	=	-	<	+	=	+	>	-	<	+	>	-	=	-	<	+	<	+	<	0.2±0.0	>	+	0.1±0.0	+	=	+	=	0.1±0.0	=	0.1±0.0	=
15:0/18:1	0.1±0.1	>	+	=	+	=	+	=	+	=	0.1±0.0	=	0.1±0.0	=	0.1±0.0	=	0.1±0.0	=	+	=	+	<	0.1±0.1	=	0.1±0.1	0.2±0.2	0.1±0.1	=	-	=	0.1±0.0	=	0.1±0.0	=
17:1/16:0	-	=	-	<	+	=	+	=	+	<	0.1±0.0	>	+	>	-	<	+	=	+	=	+	=	+	=	+	0.1±0.0	-	=	+	=	+	=	-	=
15:0/18:0	+	=	+	>	+	<	+	=	+	=	+	=	+	=	+	=	+	=	+	=	+	=	+	>	-	+	+	=	+	=	+	=	+	=
18:3/16:2	+	=	+	=	+	>	-	<	+	=	+	>	0.1±0.1	=	+	=	+	>	+	=	+	<	+	>	+	+	+	=	-	=	-	=	+	=
14:0/20:5	0.1±0.0	=	0.1±0.1	=	0.1±0.0	=	0.1±0.0	>	0.1±0.0	=	+	<	0.2±0.1	=	0.3±0.0	=	0.3±0.1	>	0.1±0.0	=	0.1±0.0	>	0.1±0.0	<	0.2±0.1	0.2±0.2	0.1±0.0	=	+	=	0.3±0.0	=	0.1±0.0	=
18:3/16:1	0.4±0.0	=	0.4±0.1	=	0.4±0.1	=	0.3±0.1	=	0.3±0.0	=	0.5±0.4	=	0.3±0.0	=	0.3±0.1	=	0.3±0.1	>	0.2±0.0	=	0.2±0.1	=	0.3±0.2	=	0.3±0.1	0.2±0.1	0.4±0.0	=	0.4±0.1	=	0.3±0.0	<	0.3±0.0	=
18:3/16:1	23.8±3.2	<	28.8±2.7	=	29.1±3.9	=	33.0±5.1	>	23.9±1.1	>	14.6±6.8	<	38.3±1.1	>	33.9±1.6	=	35.8±3.6	<	46.6±1.1	>	40.1±3.5	=	28.6±10.9	<	34.0±3.2	26.6±7.5	24.0±0.8	=	9.6±5.5	=	37.3±2.6	=	30.6±1.5	=
18:3/16:0	11.4±1.5	>	9.0±0.5	<	12.0±1.6	>	7.8±2.3	=	9.7±0.9	<	26.3±4.1	>	7.2±0.1	>	5.9±0.6	<	7.1±0.9	>	3.4±0.7	<	5.6±0.4	<	15.2±6.7	>	6.9±0.4	12.8±5.5	12.1±0.6	=	31.8±3.9	=	6.9±0.3	=	18.6±3.6	=
16:0/18:3	0.3±0.1	=	0.5±0.1	=	0.5±0.2	=	0.8±0.4	=	0.8±0.1	>	0.4±0.1	=	-	<	0.1±0.0	=	0.2±0.0	=	0.2±0.1	=	0.2±0.0	=	0.2±0.0	=	0.3±0.0	0.1±0.0	0.4±0.3	=	0.3±0.3	=	0.1±0.1	=	0.2±0.0	=
18:2/16:1	0.3±0.1	>	0.1±0.0	=	0.2±0.2	<	0.5±0.2	>	0.3±0.0	<	0.9±0.4	=	0.4±0.1	=	0.3±0.1	=	0.4±0.1	<	1.1±0.4	>	0.6±0.2	=	0.9±0.6	=	0.4±0.1	0.9±0.4	0.1±0.0	=	0.7±0.6	=	0.5±0.0	=	0.7±0.1	=
18:1/16:1	0.2±0.0	>	0.1±0.0	>	+	=	0.1±0.1	=	0.1±0.0	=	0.1±0.1	=	0.2±0.1	=	0.2±0.1	=	0.1±0.0	<	0.4±0.3	=	0.5±0.1	>	0.3±0.0	=	0.3±0.2	0.8±0.6	0.2±0.0	=	0.1±0.0	=	0.7±0.2	=	0.2±0.1	=
16:0/18:2	10.5±1.0	<	12.4±0.8	<	16.0±2.0	<	23.5±1.1	=	24.1±1.2	>	13.9±1.8	<	9.0±0.5	<	13.4±1.4	=	14.2±1.1	<	18.1±1.2	=	18.4±1.0	>	13.3±0.8	<	9.3±0.1	10.3±0.4	11.1±0.5	=	15.1±2.6	=	10.3±1.0	=	10.8±1.0	=
16:0/18:2	0.1±0.0	=	0.1±0.0	=	0.1±0.0	<	0.2±0.0	>	0.1±0.0	=	0.2±0.0	<	0.1±0.0	<	0.1±0.0	=	0.1±0.0	=	0.1±0.0	=	0.1±0.0	=	0.1±0.0	=	0.1±0.0	0.1±0.0	0.1±0.0	=	0.3±0.0	>	0.1±0.1	=	+	=
18:1/16:1	+	>	+	=	+	=	+	=	+	>	+	<	+	=	+	=	+	=	+	=	+	=	0.1±0.0	=	+	+	+	=	+	=	+	=	0.1±0.0	=
16:0/18:1	13.7±2.5	>	6.6±0.7	>	5.3±0.5	=	6.8±1.5	<	9.4±0.4	<	15.1±4.5	>	14.4±0.9	>	8.9±0.5	=	7.9±1.1	=	8.3±0.2	<	9.2±0.5	=	10.5±2.3	=	15.4±1.5	9.8±1.7	13.8±0.3	=	12.8±0.4	=	14.7±0.5	=	9.2±0.2	=
16:0/18:1	+	=	+	=	+	=	+	=	+	=	+	=	+	=	+	=	+	=	+	=	+	=	0.1±0.1	=	+	+	+	=	+	=	+	=	0.1±0.0	=
16:0/18:0	+	>	-	=	-	=	-	<	+	<	+	>	+	>	-	<	+	>	-	<	+	<	+	>	-	+	-	=	-	=	-	=	+	=
17:1/18:3	+	>	-	=	-	=	-	=	-	<	+	>	+	>	-	=	-	=	-	=	-	=	-	=	-	+	+	=	-	=	+	=	+	=
18:2/17:1	+	=	+	=	+	>	-	=	-	=	-	=	-	<	+	>	+	=	+	=	+	>	-	<	-	-	+	=	+	=	+	=	0.1±0.0	=
17:0/18:3	0.1±0.0	=	0.1±0.0	=	+	=	0.1±0.0	=	0.1±0.0	=	0.1±0.0	=	0.1±0.0	>	0.1±0.0	=	0.1±0.0	>	+	=	+	<	0.1±0.1	>	0.1±0.0	0.1±0.0	0.1±0.0	=	+	=	0.1±0.0	=	0.2±0.0	=
19:2/16:0	+	>	+	=	+	=	+	=	+	=	+	=	+	=	+	=	+	=	+	=	+	=	+	=	0.1±0.1	+	+	=	+	=	+	=	-	=
17:0/18:2	0.1±0.1	=	0.1±0.1	=	0.1±0.0	=	0.2±0.0	=	0.2±0.1	=	0.2±0.1	=	0.2±0.0	=	0.2±0.0	=	0.2±0.1	>	0.1±0.0	=	0.2±0.0	<	0.3±0.1	>	0.2±0.0	0.1±0.1	0.2±0.1	=	0.2±0.0	=	0.3±0.0	>	0.4±0.2	=
18:1/17:0	-	=	-	=	-	=	-	=	-	=	-	=	+	>	-	=	-	=	-	=	-	<	+	>	+	-	+	=	-	=	-	=	-	=
17:0/18:1	0.2±0.0	>	0.1±0.0	=	+	=	+	=	0.1±0.0	=	0.2±0.0	>	0.2±0.0	>	0.1±0.0	=	0.1±0.0	>	+	<	0.1±0.0	<	0.2±0.1	>	0.3±0.0	0.2±0.1	0.2±0.0	=	0.1±0.0	=	0.2±0.0	=	0.2±0.0	=
17:0/18:0	+	=	+	=	+	=	+	=	+	=	+	=	+	>	+	=	+	=	+	=	+	=	+	=	+	+	+	=	-	=	+	<	-	=
18:5/18:5	+	=	+	=	+	>	-	<	+	=	+	>	+	=	+	=	+	>	-	<	+	>	-	=	0.1±0.1	+	+	=	-	=	+	=	+	=
16:4/20:5	+	=	+	=	+	=	+	=	+	=	0.1±0.0	=	+	=	+	>	-	<	+	=	+	>	-	<	+	-	+	=	+	=	-	=	+	=
18:4/18:4	+	<	0.3±0.1	=	0.2±0.0	=	0.1±0.1	=	0.1±0.0	>	-	<	0.2±0.1	=	0.1±0.0	=	0.1±0.0	>	+	=	0.1±0.0	<	0.2±0.0	>	1.2±0.3	0.8±0.3	+	=	+	=	0.5±0.0	>	0.1±0.0	=
18:3/18:4	0.9±0.4	<	1.7±0.2	=	1.9±0.5	>	0.5±0.3	<	0.9±0.2	>	0.3±0.2	=	0.9±0.0	=	1.0±0.2	=	0.7±0.2	>	0.2±0.1	<	0.7±0.2	<	1.3±0.2	>	4.9±0.6	8.5±0.8	0.9±0.3	=	0.5±0.2	=	1.5±0.3	=	1.5±1.3	=
18:4/18:3	0.7±0.2	<	3.9±0.5	>	2.6±0.1	>	1.9±0.5	=	1.7±0.2	>	+	<	0.9±0.2	<	2.4±0.3	=	2.5±0.1	>	0.9±0.1	=	1.2±0.4	>	0.2±0.0	<	1.1±0.1	0.6±0.4	0.8±0.0	=	0.1±0.1	=	0.8±0.1	=	0.2±0.1	=

18:3/18:3	20.2±4.5	=	23.8±2.2	>	19.2±0.6	>	10.1±4.2	=	14.8±0.8	=	11.2±5.0	=	10.2±0.5	<	15.8±1.9	>	12.5±0.9	>	3.5±0.2	<	6.9±1.5	=	10.2±6.2	=	9.7±0.7	11.9±3.7	19.8±2.9	=	12.8±1.0	=	7.2±0.5	<	10.6±0.7	=
18:3/18:3	0.1±0.0	=	0.1±0.0	=	0.1±0.0	=	0.1±0.1	=	0.1±0.0	=	0.1±0.1	=	0.1±0.0	=	0.1±0.0	=	0.1±0.0	=	0.1±0.0	=	0.2±0.0	=	0.2±0.1	=	0.1±0.0	0.1±0.1	+	=	0.1±0.0	=	0.1±0.0	=	0.1±0.0	=
18:3/18:2	2.0±0.3	>	1.1±0.2	=	1.1±0.3	=	1.2±0.4	=	1.4±0.3	<	3.7±1.3	>	1.6±0.3	=	1.3±0.2	=	1.4±0.1	=	1.5±0.4	=	1.5±0.5	=	4.4±3.2	=	1.8±0.1	3.2±0.8	1.5±0.0	=	4.0±0.5	=	1.4±0.1	=	4.3±1.4	=
18:2/18:3	0.1±0.0	=	+	=	+	=	+	=	+	=	0.1±0.0	=	+	=	0.1±0.0	>	+	=	+	=	+	=	+	=	0.1±0.0	0.1±0.1	+	=	-	=	0.1±0.0	=	0.1±0.0	=
18:1/18:4	+	=	+	>	-	<	+	=	+	=	+	=	+	=	+	=	+	=	+	=	+	=	0.1±0.0	=	+	+	+	=	+	=	+	=	+	=
20:5/16:0	+	<	+	>	-	=	+	=	+	=	+	=	0.1±0.0	=	+	=	+	=	+	=	+	=	0.1±0.0	=	0.1±0.1	0.1±0.1	+	=	-	=	0.3±0.0	=	+	=
16:0/20:5	0.1±0.0	=	0.1±0.1	=	0.1±0.0	=	0.2±0.1	=	0.2±0.0	>	0.1±0.0	<	0.1±0.0	<	0.2±0.0	=	0.2±0.0	=	0.3±0.1	=	0.4±0.1	>	0.2±0.1	=	0.3±0.1	0.5±0.1	0.1±0.0	=	0.1±0.0	=	0.6±0.1	=	0.2±0.0	=
18:2/18:2	0.7±0.1	>	0.4±0.1	=	0.4±0.1	<	0.7±0.2	=	0.6±0.1	<	2.1±0.3	>	0.4±0.1	>	0.3±0.1	<	0.4±0.0	<	0.8±0.2	>	0.6±0.1	=	1.4±1.0	=	0.5±0.2	1.1±0.1	0.4±0.1	<	2.2±0.4	=	0.3±0.0	=	1.2±0.3	=
18:1/18:3+ 18:3/18:1	0.2±0.1	=	0.3±0.1	>	0.2±0.1	=	0.1±0.1	=	0.1±0.0	<	0.3±0.1	>	0.3±0.0	=	0.3±0.1	=	0.2±0.0	>	0.2±0.0	=	0.1±0.0	<	0.2±0.1	=	0.2±0.1	0.2±0.1	0.3±0.0	=	0.3±0.1	=	0.3±0.0	=	0.6±0.6	=
16:0/20:4	0.1±0.0	>	+	=	0.1±0.0	<	0.1±0.0	=	0.1±0.0	=	0.1±0.0	<	0.1±0.1	=	0.1±0.0	=	0.1±0.0	=	0.1±0.0	=	0.2±0.0	<	0.3±0.1	>	0.3±0.0	0.4±0.4	0.1±0.0	=	0.1±0.0	=	0.4±0.3	=	0.3±0.2	=
20:4/16:0	0.1±0.1	=	-	<	+	<	+	>	-	<	+	=	-	=	-	=	+	<	+	>	-	<	0.1±0.1	=	0.1±0.2	+	+	=	+	=	+	=	0.1±0.0	=
16:0/20:4	0.2±0.1	=	0.1±0.0	<	0.2±0.0	=	0.2±0.1	<	0.4±0.1	=	0.5±0.1	>	0.2±0.0	>	0.2±0.0	=	0.1±0.0	<	0.3±0.1	=	0.4±0.1	<	0.6±0.2	>	0.3±0.1	0.5±0.1	0.2±0.1	=	0.5±0.2	=	0.4±0.0	>	0.4±0.0	=
18:1/18:2	0.6±0.0	=	0.6±0.1	>	0.5±0.0	=	0.5±0.1	=	0.3±0.1	<	0.7±0.3	=	0.4±0.1	=	0.6±0.1	=	0.5±0.1	=	0.6±0.1	>	0.4±0.1	=	0.4±0.1	=	0.4±0.0	0.7±0.1	0.5±0.1	=	1.1±0.3	=	0.3±0.0	=	0.5±0.0	=
18:0/18:3	0.1±0.1	=	+	=	0.1±0.0	=	0.1±0.0	=	0.1±0.0	=	0.1±0.0	=	0.1±0.0	=	0.1±0.0	=	0.1±0.0	=	0.1±0.0	<	0.2±0.0	=	0.2±0.1	=	0.2±0.1	0.2±0.2	0.1±0.0	=	0.3±0.0	=	0.2±0.1	=	0.1±0.0	=
18:0/18:3	1.1±0.1	>	0.8±0.1	>	0.5±0.1	>	0.3±0.0	<	0.6±0.1	>	0.3±0.1	=	0.9±0.2	>	0.4±0.0	=	0.4±0.1	>	0.2±0.0	=	0.2±0.1	<	0.6±0.1	>	0.8±0.2	0.3±0.1	1.0±0.2	=	0.2±0.0	=	0.8±0.1	=	0.5±0.0	=
20:2/16:1	0.1±0.1	=	0.1±0.0	=	+	=	+	=	0.1±0.0	=	+	=	+	=	+	>	+	=	+	=	+	=	+	=	+	-	0.1±0.0	=	0.1±0.0	=	+	=	+	=
18:1/18:1	0.4±0.1	=	0.3±0.1	=	0.2±0.1	=	0.2±0.0	=	0.1±0.1	=	0.4±0.4	=	0.2±0.1	=	0.2±0.0	>	0.2±0.0	<	0.2±0.0	>	0.1±0.0	=	0.1±0.1	<	0.2±0.1	0.2±0.0	0.4±0.1	=	0.3±0.1	=	0.3±0.0	=	0.1±0.1	=
18:1/18:1	0.1±0.0	>	-	=	-	=	-	<	0.1±0.0	>	+	>	+	>	-	<	+	>	-	<	0.1±0.0	=	0.1±0.0	>	0.1±0.1	0.1±0.0	-	=	0.1±0.0	=	+	=	0.2±0.0	=
18:0/18:2	2.4±0.3	>	1.5±0.1	>	0.9±0.0	<	1.7±0.3	<	2.5±0.6	=	1.9±0.8	=	2.1±0.0	>	1.7±0.2	=	1.4±0.3	=	1.6±0.3	=	1.7±0.5	=	1.8±0.5	=	1.7±0.2	1.4±0.5	2.1±0.5	=	1.3±0.5	=	1.6±0.2	=	1.3±0.1	=
18:0/18:1	3.4±0.1	>	0.9±0.2	>	0.4±0.1	=	0.7±0.3	=	1.0±0.2	<	2.2±0.9	>	2.4±0.3	>	1.1±0.1	>	0.8±0.1	=	0.7±0.1	=	0.7±0.1	<	1.4±0.6	>	1.5±0.5	0.9±0.3	2.8±0.1	<	1.8±0.2	=	2.2±0.0	=	1.3±0.1	=
18:0/18:0	+	=	0.1±0.1	=	+	=	+	=	-	=	0.1±0.1	=	+	=	+	>	+	=	+	=	+	<	0.1±0.1	>	0.2±0.2	0.1±0.1	+	=	0.3±0.3	=	0.1±0.0	=	0.1±0.0	=
18:4/19:2	0.1±0.0	=	0.3±0.2	=	0.1±0.0	=	0.1±0.1	=	0.1±0.1	=	0.1±0.0	=	0.1±0.0	=	0.2±0.0	=	0.1±0.0	=	0.1±0.0	=	0.1±0.0	=	0.1±0.1	=	0.1±0.1	0.2±0.1	0.1±0.0	=	0.1±0.0	=	0.1±0.0	=	0.2±0.0	=
18:3/19:2	0.1±0.0	>	+	=	+	=	+	=	+	<	0.1±0.0	=	0.1±0.0	>	+	=	+	=	+	=	+	<	0.1±0.0	>	+	+	+	=	0.1±0.0	=	+	=	0.1±0.1	=
19:2/18:3	+	=	+	>	+	=	+	>	+	<	0.1±0.0	=	+	<	+	=	+	=	+	=	+	=	+	=	+	+	+	=	0.1±0.0	=	+	=	+	=
19:1/18:3	+	>	-	<	+	>	-	<	+	>	-	=	+	=	-	=	+	<	+	>	-	<	0.1±0.0	>	-	-	-	=	+	=	+	=	-	=
19:1/18:2	+	>	-	<	+	>	-	=	-	=	-	=	+	=	-	=	-	=	-	<	+	>	-	=	+	-	-	=	-	=	+	=	+	=
19:1/18:1	+	=	+	=	+	=	+	>	-	<	+	>	+	=	+	=	+	=	+	>	-	<	+	=	0.1±0.0	+	+	=	+	=	+	=	+	=
18:1/19:0	-	=	-	=	-	=	-	=	-	=	-	=	-	=	-	=	-	=	-	=	-	=	-	=	-	-	-	=	-	=	-	=	-	=
20:5/18:4	+	=	+	=	+	<	+	=	+	=	-	<	+	=	+	=	+	=	+	=	+	=	0.1±0.1	=	0.1±0.1	0.1±0.1	+	=	0.1±0.0	=	+	=	-	=
18:4/20:5	+	>	-	=	+	=	+	=	+	=	+	=	+	>	-	<	+	=	+	>	-	=	-	<	+	0.1±0.0	+	=	+	=	+	=	+	=
20:5/18:3	+	<	0.1±0.0	=	0.1±0.0	=	0.1±0.0	<	0.2±0.0	>	+	<	0.1±0.0	=	0.1±0.0	=	0.1±0.0	=	+	<	0.2±0.1	=	0.1±0.1	=	0.2±0.0	0.3±0.2	+	=	-	=	0.1±0.0	=	+	=
18:3/20:5	-	<	+	=	+	=	+	=	+	=	+	=	+	=	+	=	+	<	+	>	+	<	0.1±0.0	=	+	+	+	=	+	=	+	=	-	=
20:4/18:3	+	=	+	=	+	=	+	=	+	=	+	=	+	=	+	=	+	=	+	=	+	<	0.1±0.0	>	+	0.1±0.1	+	=	+	=	+	=	+	=
18:3/20:4	0.1±0.1	=	0.1±0.0	=	0.1±0.0	=	0.1±0.0	<	0.2±0.0	>	0.1±0.0	<	0.1±0.0	=	0.1±0.0	=	0.1±0.0	=	0.1±0.0	=	0.1±0.1	>	0.1±0.0	<	0.1±0.1	0.1±0.1	+	=	+	=	0.1±0.0	=	0.1±0.1	=
18:2/20:5	+	=	+	=	+	=	+	>	-	<	+	=	-	<	+	>	+	<	+	=	+	>	-	<	-	+	+	=	-	=	+	=	-	=
18:3/20:3	0.1±0.1	=	+	=	+	=	+	=	+	=	+	=	0.1±0.0	>	0.1±0.0	=	0.1±0.0	=	+	=	+	=	0.1±0.0	=	0.1±0.0	0.1±0.1	+	=	+	=	+	=	0.1±0.1	=
18:2/20:4	+	=	+	=	+	=	+	=	+	=	+	=	+	=	+	=	+	<	0.1±0.0	=	0.1±0.1	=	0.1±0.0	=	+	0.1±0.1	+	=	-	=	+	=	+	=
18:2/20:4	+	=	+	=	+	=	+	=	+	>	+	=	+	=	+	=	+	<	0.1±0.1	=	0.1±0.1	=	0.1±0.1	=	0.1±0.0	0.1±0.1	+	=	-	=	0.1±0.0	=	+	=
18:1/20:5	+	=	+	>	+	=	+	=	+	>	-	=	+	=	+	>	+	<	+	=	+	>	-	<	+	0.1±0.0	-	=	-	=	-	=	+	=
20:3/18:2	+	=	+	=	+	=	+	=	+	=	+	=	+	=	+	=	+	=	+	=	+	=	0.1±0.1	=	+	+	+	=	+	=	+	=	0.1±0.0	=
18:1/20:4	+	=	+	=	+	=	+	=	+	=	0.1±0.1	=	+	=	+	=	+	=	+	=	0.1±0.0	=	0.1±0.1	=	0.2±0.1	0.2±0.1	+	=	0.1±0.0	=	0.1±0.0	=	0.1±0.0	=
20:4/18:0	+	=	+	=	+	<	+	<	+	=	+	=	+	>	+	=	+	<	+	=	+	=	0.1±0.0	=	+	0.1±0.1	+	=	0.1±0.0	=	+	=	0.1±0.0	=

18:0/20:4	-	<	+	>	-	=	-	=	-	=	-	=	+	=	+	>	-	=	-	<	+	<	+	>	-	-	-	=	-	=	+	=	-	=
20:5/20:5	-	=	-	=	+	=	-	<	+	>	-	=	-	=	-	=	-	=	-	<	+	>	-	=	+	-	-	=	-	=	-	=	+	=
20:4/20:5	+	=	0.1±0.0	=	+	=	+	=	+	=	+	=	+	=	+	=	+	=	+	=	+	=	+	=	0.1±0.0	+	+	=	-	=	+	=	+	=
18:3/16:1-OH	+	=	+	<	+	=	+	=	+	=	+	=	+	<	0.1±0.0	=	0.1±0.0	=	+	=	+	=	+	=	+	+	+	=	-	=	0.1±0.0	>	+	=
18:3/16:0-OH	2.9±0.7	=	3.8±0.8	<	5.6±0.8	=	5.5±1.1	>	3.9±0.3	>	1.3±0.6	<	5.1±0.1	<	7.4±0.6	<	9.2±0.4	>	7.9±0.3	>	6.5±0.2	>	2.0±1.3	<	3.2±0.1	1.3±0.7	3.4±0.4	=	1.0±0.4	=	3.8±0.8	=	1.9±1.0	=
18:2/16:0-OH	0.1±0.0	=	+	=	0.1±0.1	=	0.2±0.1	>	0.1±0.0	=	0.2±0.0	=	0.1±0.0	>	0.1±0.0	<	0.1±0.0	<	0.3±0.1	=	0.2±0.1	=	0.2±0.1	<	+	0.1±0.1	+	=	0.1±0.1	=	0.1±0.0	=	0.1±0.1	=
SFA/SFA	0.2±0.0	=	0.1±0.1	=	0.1±0.0	=	0.1±0.0	=	0.1±0.0	<	0.3±0.1	>	0.1±0.1	>	0.1±0.0	=	+	=	+	=	0.1±0.0	<	0.4±0.1	>	0.3±0.2	0.5±0.2	0.1±0.0	=	0.4±0.4	=	0.2±0.1	=	0.2±0.1	=
SFA/MUFA	18.0±2.6	>	7.8±1.0	>	6.2±0.6	=	8.0±1.9	<	10.7±0.5	<	17.9±5.3	>	17.8±0.9	>	10.8±0.7	=	9.3±1.2	=	9.2±0.3	<	10.6±0.4	=	13.0±3.1	>	18.0±2.1	12.0±1.7	17.5±0.3	=	15.0±0.4	=	19.0±0.3	=	11.3±0.5	=
MUFA/SFA	-	=	-	=	-	=	-	=	-	=	-	=	-	=	-	=	-	=	-	=	-	=	-	=	-	-	-	=	-	=	-	=	-	=
SFA/PUFA	15.5±1.1	=	16.1±0.9	<	19.1±2.2	<	27.9±1.3	=	29.8±1.6	>	18.1±2.7	<	13.7±0.4	<	17.6±1.7	=	18.0±1.5	<	21.6±1.4	=	22.5±1.0	>	18.6±1.2	<	14.0±0.5	15.2±0.7	16.1±1.6	=	18.8±1.6	=	15.9±1.4	=	15.3±0.7	<
PUFA/SFA	14.7±0.9	>	12.9±0.3	<	17.7±0.9	>	13.5±1.1	=	13.8±0.7	<	28.0±3.5	>	12.7±0.2	<	13.5±0.2	<	16.5±1.0	>	11.8±0.9	=	12.4±0.3	=	17.8±5.1	>	10.7±0.5	14.5±4.7	15.9±1.0	=	33.1±3.4	=	11.3±0.2	<	20.9±2.8	=
MUFA/MUFA	0.7±0.1	>	0.4±0.1	=	0.3±0.1	=	0.3±0.0	=	0.2±0.1	=	0.6±0.3	=	0.5±0.1	=	0.5±0.1	>	0.3±0.1	<	0.7±0.3	=	0.7±0.1	=	0.6±0.0	=	0.7±0.0	1.1±0.6	0.6±0.1	=	0.5±0.2	=	1.2±0.2	=	0.5±0.3	=
MUFA/PUFA	0.9±0.1	=	0.9±0.1	>	0.6±0.1	=	0.7±0.2	=	0.5±0.1	=	0.9±0.4	=	0.8±0.2	=	0.9±0.1	=	0.8±0.1	=	0.8±0.1	=	0.6±0.2	=	0.8±0.2	=	0.9±0.2	1.1±0.1	0.8±0.0	=	1.3±0.4	=	0.9±0.0	=	0.9±0.2	=
PUFA/ MUFA	24.6±3.3	<	29.6±2.7	=	29.9±3.7	=	34.0±5.2	>	24.5±1.1	>	16.1±6.9	<	39.2±1.1	>	34.7±1.5	=	36.6±3.5	<	48.0±1.5	>	40.9±3.3	=	29.9±11.2	<	34.9±3.2	27.8±7.8	24.7±0.8	=	10.9±6.3	=	38.3±2.5	=	32.0±2.0	=
PUFA/PUFA	25.3±5.2	=	32.1±3.2	>	26.0±1.1	>	15.3±4.9	=	20.3±1.6	=	17.8±6.3	=	14.8±0.2	<	21.7±1.8	>	18.2±1.2	>	7.8±0.5	<	11.8±2.8	=	18.6±10.6	=	20.2±1.1	27.4±3.7	24.0±3.0	=	19.9±1.8	=	12.5±0.9	=	18.7±0.4	=

PE	The lower part of the blades of infected *U. pinnatifida* samples												The upper intact part of the blades of infected *U. pinnatifida* samples												The upper part of the blades of infected *U. pinnatifida* samples with endophyte		The lower part of the blades of uninfected *U. pinnatifida* samples				The upper part of the blades of uninfected *U. pinnatifida* samples			
	Nov.		Dec.		Jan.		Feb.		Apr.		Jun.	T	Nov.		Dec.		Jan.		Feb.		Apr.		Jun.	T	Nov.	Jun.	Nov.	S	Jun.	S	Nov.	S	Jun.	S
16:1/14:0	-	=	-	<	+	<	+	=	+	>	-	<	+	>	-	=	-	<	+	=	+	<	0.3±0.2	>	+	0.4±0.3	-	=	-	=	+	=	0.1±0.1	=
15:0/15:0	-	=	-	=	-	<	+	>	-	=	-	<	-	=	-	=	-	<	+	>	+	=	+	=	+	+	-	=	+	=	+	=	+	=
16:1/16:1	+	=	+	=	+	=	+	=	+	=	0.1±0.1	=	0.1±0.0	=	0.2±0.1	=	0.1±0.1	<	0.3±0.1	>	0.2±0.1	<	0.4±0.1	=	0.3±0.2	0.9±0.7	+	=	+	=	0.3±0.0	=	0.2±0.2	=
14:0/18:2	0.1±0.0	=	+	=	+	=	+	=	+	=	+	=	+	=	+	=	0.1±0.0	=	0.1±0.0	=	0.1±0.0	=	0.1±0.1	=	0.1±0.0	0.1±0.0	0.1±0.0	=	0.1±0.0	=	+	=	+	=
14:0/18:1	0.1±0.0	>	+	<	+	=	+	=	+	<	0.1±0.0	=	0.1±0.0	=	+	=	+	<	0.1±0.0	>	+	=	0.2±0.2	=	0.2±0.0	0.4±0.3	0.1±0.0	=	0.1±0.0	=	0.1±0.0	=	0.1±0.2	=
16:0/16:1	+	>	-	<	+	>	-	<	+	=	+	>	+	>	-	<	+	=	+	<	+	=	+	>	+	+	-	=	+	=	-	=	-	=
16:0/16:0	+	>	+	<	+	=	+	=	+	<	0.1±0.0	>	+	>	+	=	+	=	+	=	+	=	+	=	+	+	+	=	+	=	+	=	+	=
16:3/18:4	+	>	-	<	+	=	+	>	-	=	-	<	+	>	-	<	+	>	+	>	-	<	+	>	+	+	+	=	+	=	+	=	-	=
16:3/18:3	+	=	+	=	+	=	-	<	+	>	-	=	+	>	+	=	+	>	-	<	+	<	+	>	+	+	+	=	-	=	+	=	+	=
20:5/14:0	+	=	+	=	+	=	+	>	+	=	+	=	+	=	+	>	+	=	+	=	+	=	+	=	+	+	+	=	-	=	+	=	+	=
14:0/20:5	1.1±0.2	<	1.4±0.1	=	1.4±0.3	=	1.6±0.5	=	1.9±0.3	>	0.4±0.1	<	1.0±0.2	=	1.0±0.0	>	0.8±0.0	=	0.8±0.2	<	1.0±0.1	>	0.6±0.1	=	1.1±0.1	1.0±0.1	1.6±0.1	>	0.2±0.1	=	1.2±0.0	=	0.6±0.1	=
14:0/20:4	0.1±0.0	=	0.1±0.0	=	0.1±0.0	=	0.1±0.0	=	0.1±0.0	>	+	=	+	=	0.1±0.0	<	0.1±0.0	=	0.1±0.0	=	+	=	+	=	0.1±0.1	+	0.2±0.0	=	+	=	0.1±0.0	=	+	=
14:0/20:4	3.6±0.7	>	1.1±0.1	=	1.2±0.2	=	1.1±0.3	<	2.0±0.3	<	3.9±0.6	>	1.9±0.2	>	1.2±0.1	>	1.0±0.1	=	0.9±0.1	=	1.0±0.1	<	2.4±0.3	>	1.7±0.5	2.3±0.8	4.1±0.2	=	3.6±0.1	=	1.8±0.0	=	2.6±0.3	=
18:2/16:1	+	=	+	=	+	=	+	=	+	=	+	=	+	=	+	=	+	=	0.2±0.3	=	+	<	+	=	0.1±0.0	0.1±0.0	+	=	+	=	+	=	+	=
18:2/16:1	-	=	-	=	-	<	+	=	+	=	+	=	+	>	-	<	+	<	0.4±0.2	>	+	<	0.1±0.1	<	0.1±0.0	0.2±0.1	+	=	-	=	0.4±0.0	=	-	=
18:1/16:2	0.1±0.0	>	+	=	0.1±0.0	=	+	<	0.1±0.0	=	0.1±0.0	=	0.1±0.0	=	0.2±0.1	=	0.1±0.1	=	0.1±0.0	=	0.1±0.0	=	0.1±0.0	=	0.2±0.2	0.1±0.0	0.2±0.1	=	-	=	0.2±0.2	=	0.1±0.1	=
16:0/18:3	0.3±0.0	>	0.2±0.0	=	0.3±0.1	>	0.1±0.0	<	0.2±0.0	=	0.2±0.2	=	0.1±0.1	=	0.1±0.0	=	0.1±0.0	>	0.1±0.0	=	0.1±0.0	=	0.1±0.0	=	0.1±0.1	+	0.4±0.1	=	0.2±0.0	=	0.1±0.0	=	0.1±0.0	=
18:1/16:1	+	=	+	=	+	=	+	=	+	=	+	=	0.2±0.1	=	0.2±0.2	=	0.1±0.1	<	0.4±0.1	=	0.3±0.1	=	0.7±0.4	=	0.7±0.3	1.8±1.5	0.1±0.0	=	+	=	1.0±0.1	>	0.4±0.5	=
16:0/18:2	0.4±0.1	>	0.2±0.1	=	0.2±0.0	=	0.1±0.1	=	0.2±0.1	<	0.5±0.1	>	0.2±0.1	=	0.2±0.0	=	0.2±0.1	=	0.2±0.0	=	0.1±0.0	=	0.1±0.0	=	0.1±0.0	0.1±0.0	0.3±0.0	=	0.4±0.2	=	0.1±0.1	=	0.3±0.2	=
16:0/18:1	0.2±0.1	>	+	>	+	=	+	=	+	<	0.3±0.1	>	0.1±0.0	=	+	=	+	=	0.1±0.0	>	+	=	0.1±0.1	=	0.2±0.3	0.2±0.1	0.1±0.0	=	0.2±0.0	=	0.1±0.0	=	0.1±0.0	=

18:0/16:0	+	>	-	<	+	>	+	=	+	=	+	=	+	=	-	=	-	=	-	=	-	<	+	>	+	-	-	=	-	=	-	=	-	=
18:4/17:2	-	=	-	=	+	=	+	=	+	>	-	<	-	=	-	=	-	<	+	>	-	=	-	<	+	-	+	=	+	=	+	=	-	=
20:5/15:0	0.1±0.0	=	0.1±0.0	=	0.1±0.0	=	0.1±0.0	=	0.1±0.0	>	+	<	0.1±0.0	=	0.1±0.0	<	0.1±0.0	>	0.1±0.0	=	0.1±0.0	=	0.2±0.0	=	0.2±0.0	0.2±0.1	0.1±0.0	=	+	=	0.1±0.0	=	0.1±0.2	=
20:4/15:0	0.2±0.0	>	0.1±0.0	=	0.1±0.0	=	0.1±0.0	=	0.1±0.0	<	0.2±0.0	>	0.2±0.1	>	0.1±0.0	=	0.1±0.0	=	0.1±0.0	=	0.1±0.0	<	0.5±0.1	>	0.2±0.1	0.6±0.2	0.2±0.0	>	0.3±0.1	=	0.2±0.0	=	0.5±0.2	=
17:1/18:2	-	=	-	<	+	=	+	>	+	>	-	<	-	=	-	=	-	<	+	=	+	>	-	<	-	+	-	=	-	=	-	=	+	=
18:3/18:4	0.1±0.0	<	0.2±0.0	>	0.1±0.0	>	+	=	0.1±0.0	=	+	=	+	<	0.1±0.1	=	0.1±0.0	>	+	>	+	=	+	<	0.1±0.0	+	0.1±0.0	=	+	=	+	=	+	=
18:4/18:3	+	=	0.1±0.0	<	0.2±0.1	=	0.1±0.0	=	0.1±0.0	>	+	<	+	=	+	=	0.1±0.0	>	+	<	0.1±0.0	>	+	=	+	+	0.1±0.0	=	+	=	+	<	+	=
18:3/18:3	0.1±0.1	<	0.2±0.0	>	0.1±0.1	=	0.1±0.0	=	0.1±0.0	>	+	=	+	<	0.2±0.0	>	0.1±0.0	=	+	=	+	=	+	=	+	0.1±0.0	0.2±0.0	=	+	=	+	=	+	=
16:1/20:5	0.1±0.0	<	0.4±0.1	=	0.5±0.1	=	0.5±0.2	>	0.2±0.0	>	0.1±0.1	<	0.1±0.0	<	0.2±0.0	=	0.3±0.1	=	0.3±0.1	=	0.2±0.1	>	0.1±0.1	<	0.1±0.1	0.2±0.1	0.1±0.0	=	0.1±0.0	=	0.2±0.1	=	0.1±0.0	=
18:3/18:2	0.1±0.0	<	0.2±0.1	=	0.2±0.1	>	+	<	0.1±0.0	=	+	=	+	=	0.1±0.0	=	0.1±0.0	=	+	=	+	=	+	=	+	+	0.1±0.0	=	0.1±0.0	=	+	=	+	=
18:2/18:3	+	=	+	=	+	>	+	=	+	=	+	=	0.1±0.0	>	+	=	+	=	+	=	+	=	0.1±0.1	=	+	+	+	=	+	=	+	=	+	=
16:1/20:4	0.3±0.1	=	0.3±0.0	=	0.3±0.1	=	0.3±0.0	=	0.2±0.1	=	0.2±0.0	<	0.1±0.0	<	0.2±0.0	=	0.2±0.1	=	0.2±0.0	=	0.2±0.0	>	0.1±0.0	<	0.2±0.1	0.2±0.1	0.3±0.0	=	0.1±0.0	=	0.1±0.1	=	0.1±0.0	=
16:0/20:5	5.3±1.0	<	10.1±0.3	<	12.7±2.2	=	11.9±0.8	=	10.9±0.5	>	2.2±0.8	<	5.5±0.3	<	7.4±0.8	<	8.4±0.2	=	8.4±0.1	<	9.8±0.2	>	3.8±0.4	<	6.4±0.3	6.3±0.5	6.2±0.4	=	1.7±0.2	=	7.5±0.6	=	3.3±1.7	=
18:2/18:2	+	=	+	=	+	=	0.1±0.0	=	+	=	0.1±0.0	=	+	=	+	>	+	<	+	=	+	=	+	=	+	+	+	=	-	=	+	=	-	=
18:3/18:1	+	=	-	<	+	=	+	=	+	=	+	=	+	>	-	<	+	=	+	=	+	<	+	>	0.1±0.0	0.1±0.1	+	=	+	=	+	=	+	=
20:4/16:0	0.6±0.2	=	0.7±0.0	=	0.8±0.3	=	0.6±0.1	=	0.6±0.0	>	0.1±0.1	<	0.3±0.1	=	0.3±0.1	=	0.4±0.1	=	0.4±0.1	>	0.3±0.1	>	0.1±0.0	<	0.5±0.1	0.2±0.1	0.7±0.1	=	0.1±0.1	=	0.2±0.0	=	0.3±0.1	=
16:0/20:4	11.2±0.6	>	4.3±0.4	<	5.4±0.4	<	6.3±0.6	<	8.0±0.4	<	19.1±1.5	>	9.1±0.3	>	6.0±0.1	=	6.2±0.4	<	8.7±0.9	=	8.3±0.8	<	15.8±3.1	>	7.5±0.5	13.3±1.5	12.7±0.5	=	19.8±1.8	=	9.5±0.2	=	15.2±1.0	=
18:1/18:2	0.1±0.0	=	+	=	+	=	+	=	+	=	+	=	+	=	+	=	+	=	+	=	+	<	0.1±0.0	>	0.1±0.0	0.1±0.0	+	=	0.1±0.0	=	0.1±0.0	>	0.1±0.0	=
16:0/20:3	0.4±0.0	>	0.3±0.1	=	0.4±0.0	=	0.3±0.0	=	0.4±0.0	>	0.3±0.0	=	0.2±0.1	=	0.2±0.1	=	0.2±0.1	=	0.3±0.0	>	0.2±0.0	=	0.2±0.2	=	0.1±0.1	0.2±0.0	0.5±0.1	=	0.4±0.0	>	0.2±0.0	=	0.2±0.1	=
18:1/18:1	+	=	+	=	+	=	+	=	+	=	+	=	+	=	+	=	+	=	+	=	+	<	0.1±0.0	>	0.1±0.1	0.1±0.0	+	=	+	=	0.1±0.0	>	0.1±0.1	=
18:1/18:1	0.1±0.1	=	+	>	+	<	+	<	0.1±0.0	>	+	=	+	>	+	=	+	<	+	=	0.1±0.0	=	0.1±0.1	=	0.1±0.1	0.1±0.1	+	=	+	=	+	=	+	=
18:0/18:2	0.1±0.0	=	0.1±0.0	=	0.1±0.0	=	+	>	+	=	+	=	0.1±0.0	=	+	=	0.1±0.0	>	+	=	+	=	+	=	0.1±0.1	+	0.1±0.0	=	+	=	+	=	+	=
16:0/20:1	-	=	-	<	+	=	+	>	-	=	-	<	-	=	-	<	+	>	-	<	+	>	-	=	-	-	+	=	+	=	+	=	-	=
18:0/18:1	+	>	+	=	+	=	+	<	+	=	+	>	+	=	+	=	+	=	+	=	+	=	+	>	+	+	+	=	-	=	0.1±0.1	=	+	=
18:0/18:0	0.1±0.0	=	+	=	+	>	+	=	+	<	0.1±0.1	>	+	=	+	=	+	=	+	>	+	<	0.1±0.1	>	0.1±0.0	0.1±0.0	+	=	0.2±0.0	=	+	=	0.1±0.0	=
20:5/18:5	+	<	+	=	+	>	+	=	+	=	+	=	+	<	+	=	0.1±0.0	=	+	=	+	<	+	=	+	+	+	=	0.1±0.0	=	+	=	+	=
20:5/18:4	0.5±0.1	<	1.1±0.0	>	0.7±0.0	>	0.2±0.1	<	0.4±0.0	>	0.2±0.1	=	0.3±0.0	<	0.8±0.1	=	0.7±0.1	>	0.2±0.0	=	0.2±0.1	=	0.2±0.1	=	0.5±0.1	1.0±0.2	0.8±0.0	=	0.1±0.1	=	0.3±0.0	=	0.2±0.1	=
18:4/20:5	+	=	+	=	+	=	+	=	+	=	+	=	+	=	+	=	+	=	+	<	+	=	+	=	+	0.1±0.1	+	=	-	=	+	=	-	=
20:3/18:5	+	>	+	=	+	=	+	>	-	=	-	<	+	=	+	=	+	=	+	=	+	>	-	<	+	+	-	=	-	=	-	=	-	=
20:5/18:3	0.5±0.0	<	4.6±0.8	=	3.6±1.0	>	1.9±0.4	=	1.8±0.1	>	0.1±0.1	<	0.5±0.0	<	2.7±0.5	=	2.8±0.8	>	0.9±0.2	<	1.2±0.1	>	0.2±0.2	<	0.7±0.2	0.2±0.1	0.8±0.0	>	0.3±0.0	=	0.5±0.0	=	0.2±0.2	=
18:3/20:5	1.4±0.3	=	1.4±0.2	=	1.2±0.4	=	1.1±0.8	=	0.8±0.1	=	1.0±0.2	=	0.4±0.2	<	1.1±0.1	=	1.2±0.1	=	0.4±0.0	=	0.4±0.1	=	0.7±0.4	=	0.8±0.4	1.4±0.5	1.5±0.3	=	0.7±0.0	=	0.3±0.1	=	0.6±0.1	=
20:4/18:4	+	=	+	<	+	>	+	=	+	=	+	=	+	=	+	=	+	=	+	=	+	=	+	=	0.1±0.0	+	+	=	+	=	+	=	+	=
18:4/20:4	+	=	+	<	+	=	+	=	+	>	-	<	+	=	+	=	+	=	+	<	+	=	+	=	0.1±0.0	+	+	=	-	=	+	=	+	=
20:4/18:3	2.6±0.5	<	8.4±1.1	=	7.8±2.0	=	6.4±1.4	=	5.4±0.3	>	1.5±0.7	<	2.0±0.4	<	6.5±0.5	>	5.6±0.5	>	4.5±0.6	=	4.4±0.3	>	1.1±0.4	<	2.0±0.3	1.4±0.1	3.2±0.2	=	1.2±0.0	=	1.7±0.1	=	1.1±0.0	=
18:3/20:4	0.1±0.0	=	0.1±0.0	=	0.1±0.0	>	+	=	0.1±0.0	=	0.1±0.0	>	0.1±0.0	=	0.1±0.0	=	0.1±0.0	=	0.1±0.0	=	0.1±0.0	=	0.1±0.0	>	0.1±0.0	+	0.1±0.0	=	0.1±0.0	=	0.1±0.0	=	0.1±0.0	=
20:4/18:3	+	=	+	=	+	=	+	>	+	>	-	<	+	=	+	=	+	=	+	=	+	=	+	=	+	+	+	=	+	=	+	=	+	=
18:3/20:3	+	<	+	=	+	<	0.2±0.1	=	0.3±0.0	>	+	<	+	<	+	=	+	<	0.1±0.1	=	0.1±0.0	>	+	<	0.1±0.0	+	+	=	+	=	+	=	+	=
20:4/18:2	+	>	-	=	-	=	0.2±0.2	=	-	=	-	=	+	=	-	=	-	<	0.1±0.1	>	+	>	-	<	-	+	-	=	-	=	-	=	+	=
18:2/20:4	1.7±0.3	=	1.8±0.3	=	2.0±0.4	=	2.4±0.5	=	2.4±0.2	=	2.5±0.4	=	1.5±0.0	<	2.9±0.7	=	2.3±0.2	<	2.8±0.4	=	2.8±0.2	>	1.8±0.3	<	1.3±0.2	1.4±0.2	1.8±0.4	=	2.5±0.3	=	1.3±0.1	=	1.9±0.3	=
18:1/20:5	1.4±0.2	<	4.0±0.6	>	2.8±0.4	=	2.3±0.4	=	2.1±0.1	>	0.3±0.2	<	0.8±0.0	<	1.4±0.4	=	1.4±0.1	>	1.0±0.0	=	0.8±0.2	>	0.2±0.0	<	0.8±0.0	0.4±0.1	1.4±0.1	=	0.3±0.2	=	0.7±0.0	=	0.2±0.0	=
18:1/20:5	+	<	+	=	+	=	+	=	+	=	+	=	+	<	+	=	+	=	+	=	+	=	+	=	0.1±0.0	+	+	=	+	=	+	=	+	=
16:1/22:5	+	=	+	=	+	=	+	=	+	=	+	=	+	=	+	=	+	>	+	=	+	=	+	=	+	+	+	=	-	=	+	=	+	=
16:0/22:6	+	=	+	=	+	=	+	=	+	=	+	=	+	=	+	=	+	=	+	=	+	=	+	=	-	+	+	=	+	=	+	=	+	=

18:2/20:3	0.1±0.0	<	0.1±0.0	=	0.1±0.0	=	0.1±0.1	=	0.1±0.0	>	+	<	0.1±0.0	=	0.1±0.0	=	0.1±0.0	=	0.1±0.0	>	0.1±0.0	>	+	<	0.1±0.0	0.1±0.0	0.1±0.0	=	+	=	+	=	+	=
18:1/20:4	1.7±0.4	>	1.0±0.2	>	0.8±0.1	=	0.9±0.3	=	1.0±0.1	<	1.8±0.5	>	0.8±0.1	=	0.8±0.3	=	0.6±0.0	=	0.6±0.1	>	0.5±0.1	=	0.4±0.1	=	0.5±0.1	0.4±0.1	1.5±0.0	=	1.6±0.4	=	0.6±0.0	<	0.4±0.1	=
18:0/20:5	1.3±0.4	=	1.6±0.2	>	1.0±0.1	<	1.3±0.1	<	1.7±0.3	>	0.6±0.2	<	1.5±0.1	=	1.3±0.3	=	1.3±0.0	=	1.1±0.4	=	1.2±0.4	=	1.1±0.7	=	1.7±0.1	1.5±0.7	1.2±0.2	=	0.4±0.3	=	1.4±0.1	=	1.0±0.3	=
18:1/20:3	+	>	+	=	+	<	+	=	+	=	-	<	+	=	+	<	+	=	+	=	+	=	-	<	+	+	+	=	-	=	-	=	-	=
20:4/18:0	0.1±0.1	=	0.1±0.0	>	+	=	+	=	+	=	0.1±0.0	=	+	=	+	=	+	=	+	=	+	<	+	=	+	+	0.1±0.0	=	-	=	+	=	+	=
18:0/20:4	2.2±0.2	>	0.7±0.2	=	0.4±0.1	<	0.7±0.1	<	1.1±0.1	<	4.2±0.8	>	2.6±0.2	>	1.1±0.1	=	1.0±0.1	=	1.0±0.2	=	1.1±0.2	<	4.2±1.2	>	1.9±0.1	2.9±0.4	2.2±0.1	=	3.9±0.5	=	2.0±0.3	=	4.7±1.2	=
20:5/20:5	3.4±0.6	<	10.4±1.0	=	9.7±0.5	>	6.5±0.3	=	6.2±0.2	>	0.4±0.3	<	3.9±0.5	<	8.7±0.6	=	9.8±1.3	>	4.6±0.9	=	4.8±0.3	>	1.1±0.6	<	6.8±0.7	4.9±0.7	3.6±0.1	=	0.1±0.0	=	5.1±0.2	>	0.8±0.3	=
22:5/18:4	0.4±0.0	<	1.2±0.2	>	0.8±0.2	=	0.7±0.2	=	0.7±0.1	>	+	<	0.4±0.1	<	0.7±0.1	<	0.9±0.1	>	0.5±0.3	=	0.6±0.1	>	0.1±0.1	<	0.8±0.1	0.5±0.1	0.4±0.0	=	+	=	0.8±0.0	>	0.2±0.1	=
20:4/20:5	24.0±1.5	<	27.0±1.4	=	28.4±4.5	=	29.9±2.6	>	25.5±0.7	>	9.1±3.2	<	28.2±0.9	=	29.5±1.5	=	30.9±2.9	=	29.8±1.1	<	32.0±1.3	>	13.0±3.3	<	30.3±0.4	19.7±1.4	22.3±3.3	=	6.6±1.6	=	28.8±1.0	=	12.7±3.6	=
18:3/22:5	+	<	+	=	+	=	+	=	+	=	+	=	+	=	+	=	+	=	+	=	+	<	+	=	+	0.1±0.0	+	=	+	=	+	=	+	=
20:3/20:5+ 20:5/20:3	2.1±0.3	<	2.9±0.2	>	2.5±0.1	=	2.6±0.5	=	2.6±0.1	>	0.4±0.1	<	1.5±0.2	<	2.5±0.1	=	2.8±0.4	=	2.5±0.4	>	1.9±0.3	>	0.7±0.1	<	2.0±0.3	1.0±0.1	2.8±0.6	=	0.4±0.1	=	1.9±0.1	=	0.8±0.3	=
20:4/20:4	25.1±0.8	>	8.2±1.3	=	9.9±2.2	=	13.1±1.8	<	15.5±0.7	<	41.2±4.9	>	29.5±0.2	>	15.1±2.6	=	13.9±0.8	<	21.4±2.0	>	18.5±0.9	<	38.4±3.8	>	22.5±0.7	25.6±2.8	21.5±0.6	<	46.1±2.5	=	24.6±1.5	=	38.9±5.4	=
20:4/20:3	1.0±0.1	>	0.3±0.3	=	0.1±0.2	<	0.7±0.4	=	0.7±0.3	>	0.1±0.0	<	0.1±0.1	=	0.1±0.2	<	0.8±0.3	=	0.8±0.4	=	0.7±0.1	>	0.3±0.2	=	0.1±0.1	0.1±0.1	0.6±0.9	=	0.8±0.0	>	+	=	0.1±0.0	=
20:3/20:4	0.4±0.2	=	0.4±0.1	=	0.4±0.0	=	0.3±0.4	=	0.6±0.1	=	0.8±0.1	>	0.3±0.0	=	0.5±0.0	=	0.3±0.0	=	0.3±0.3	=	0.2±0.0	=	0.2±0.0	=	0.1±0.0	0.2±0.1	-	=	-	=	-	=	0.4±0.0	>
20:4/20:2	0.1±0.1	=	0.1±0.0	=	0.2±0.0	=	0.2±0.0	=	0.2±0.0	>	+	<	0.1±0.0	=	0.1±0.0	=	0.2±0.0	=	0.2±0.0	=	0.2±0.1	>	0.1±0.1	<	0.1±0.0	0.1±0.0	0.1±0.1	=	+	=	0.1±0.0	=	0.1±0.0	=
20:4/20:2	+	=	+	=	+	=	+	=	+	=	+	=	+	=	0.1±0.0	=	+	=	+	=	+	=	+	<	0.1±0.0	+	+	=	+	=	+	=	+	=
20:2/20:4	+	=	+	=	+	=	+	=	+	>	-	<	+	=	+	=	+	=	+	=	+	>	-	=	+	+	+	=	+	=	-	=	-	=
20:1/20:5	+	=	+	=	+	=	+	=	+	<	+	=	0.1±0.0	>	+	=	+	=	+	=	+	>	-	<	-	+	+	=	+	=	+	=	+	=
20:4/20:1	0.1±0.0	>	+	>	+	<	+	=	+	=	+	=	+	=	+	>	+	=	+	=	+	=	+	=	+	+	+	=	0.1±0.0	=	+	=	0.7±0.9	=
20:0/20:5	1.5±0.3	<	2.7±0.1	>	1.9±0.3	<	2.6±0.3	=	2.9±0.2	>	0.8±0.3	<	1.9±0.3	<	2.8±0.3	=	2.6±0.3	>	2.1±0.3	<	2.9±0.3	>	1.7±0.9	=	2.1±0.1	2.1±0.4	1.8±0.1	=	0.4±0.2	=	2.2±0.3	=	1.5±0.3	=
20:0/20:4	3.1±0.2	>	1.0±0.1	=	0.8±0.3	<	1.3±0.1	<	1.8±0.1	<	5.7±0.6	>	3.4±0.4	>	2.3±0.3	>	1.8±0.2	=	2.0±0.2	<	2.4±0.1	<	6.9±0.6	>	3.0±0.1	4.8±0.5	2.9±0.4	=	5.8±0.6	=	3.1±0.3	=	7.4±1.3	=
20:4/22:2	+	>	+	>	-	=	-	=	-	<	+	>	-	<	+	=	+	>	-	<	+	=	+	>	-	+	-	=	+	=	+	=	-	=
20:5/22:0	+	<	0.1±0.0	=	0.1±0.0	<	0.2±0.0	=	0.1±0.1	>	+	<	+	<	0.1±0.0	=	0.1±0.0	=	0.1±0.0	<	0.2±0.0	>	0.1±0.1	=	0.1±0.0	0.1±0.1	0.1±0.0	=	+	=	0.1±0.0	=	+	=
22:0/20:5	+	>	-	=	-	<	+	=	+	>	-	<	+	>	-	=	-	<	+	>	+	>	-	<	-	0.1±0.0	-	=	-	=	-	=	+	=
22:0/20:4	0.2±0.1	>	+	>	+	<	0.1±0.0	=	0.1±0.0	<	0.2±0.1	>	0.1±0.0	>	0.1±0.0	=	0.1±0.0	=	0.1±0.0	=	0.1±0.0	<	0.2±0.1	>	0.1±0.0	0.2±0.0	0.1±0.0	=	0.2±0.1	=	0.1±0.0	=	0.2±0.1	=
22:6/22:6	-	=	-	=	-	=	-	=	-	=	-	=	-	=	-	<	+	>	-	=	-	=	-	=	-	-	-	=	+	=	-	=	-	=
22:5/22:6	-	=	-	=	-	=	-	=	-	<	+	>	-	=	-	=	-	=	-	<	+	>	-	=	-	-	+	=	-	=	-	=	-	=
22:4/22:4	-	=	-	=	-	=	-	=	-	<	+	>	-	=	-	=	-	=	-	=	-	=	-	=	-	-	-	=	-	=	-	=	-	=
22:5/22:2	-	=	-	=	-	=	-	=	-	=	-	=	+	>	-	<	+	>	-	=	-	=	-	=	+	-	-	=	-	=	+	=	-	=
22:5/22:1	+	=	+	=	+	=	+	=	+	=	+	=	-	<	+	<	+	=	+	=	+	>	-	<	-	+	+	=	-	=	+	=	+	=
24:0/20:5	0.1±0.1	=	0.2±0.0	=	0.1±0.0	<	0.2±0.1	=	0.2±0.0	>	+	<	0.1±0.0	=	0.1±0.0	=	0.1±0.0	=	0.1±0.1	=	0.1±0.0	=	0.1±0.0	=	0.2±0.1	0.1±0.1	0.1±0.0	=	0.1±0.0	=	0.1±0.0	=	0.1±0.0	=
24:0/20:4	0.2±0.0	>	0.1±0.0	=	+	=	0.1±0.0	=	0.1±0.0	<	0.4±0.0	>	0.1±0.0	=	0.1±0.0	=	+	=	0.1±0.1	=	0.1±0.0	<	0.2±0.1	>	0.1±0.1	0.2±0.1	0.2±0.0	=	0.3±0.1	=	0.1±0.0	=	0.3±0.1	=
SFA/SFA	0.1±0.0	=	+	<	0.1±0.0	=	+	=	+	<	0.2±0.1	>	0.1±0.0	>	+	=	+	=	+	=	+	<	0.2±0.0	>	0.1±0.0	0.2±0.0	0.1±0.0	=	0.2±0.0	=	0.1±0.0	=	0.1±0.0	<
SFA/MUFA	0.3±0.1	>	0.1±0.0	=	0.1±0.0	=	0.1±0.0	=	0.1±0.0	<	0.4±0.1	>	0.1±0.0	=	0.1±0.0	=	0.1±0.0	<	0.2±0.0	>	0.1±0.0	=	0.3±0.3	=	0.4±0.3	0.7±0.4	0.2±0.0	=	0.3±0.0	=	0.3±0.1	=	0.3±0.2	=
MUFA/SFA	-	=	-	=	-	=	-	=	-	=	-	=	-	=	-	=	-	=	-	=	-	=	-	=	-	-	-	=	-	=	-	=	-	=
SFA/PUFA	31.0±0.6	>	24.0±0.9	=	25.9±1.9	=	27.8±1.1	<	31.5±0.5	<	38.6±2.2	>	27.8±0.8	>	23.9±1.4	=	23.9±1.2	<	25.8±0.6	<	28.5±0.7	<	37.6±1.0	>	26.5±0.2	35.1±3.2	34.7±1.1	=	37.7±0.2	=	29.5±0.8	=	37.7±0.5	=
PUFA/SFA	1.0±0.2	=	1.1±0.0	=	1.1±0.3	=	1.0±0.2	=	0.9±0.1	>	0.4±0.1	<	0.6±0.1	=	0.7±0.1	=	0.7±0.1	=	0.7±0.0	=	0.6±0.1	=	0.8±0.1	=	1.0±0.1	1.1±0.3	1.2±0.1	=	0.4±0.1	=	0.7±0.0	=	1.0±0.5	=
MUFA/MUFA	0.1±0.0	>	+	=	+	=	0.1±0.1	=	+	=	0.1±0.0	=	0.3±0.1	=	0.4±0.4	=	0.2±0.1	<	0.7±0.2	=	0.5±0.2	=	1.1±0.6	=	1.0±0.5	2.7±2.3	0.1±0.0	=	0.1±0.1	=	1.4±0.0	>	0.6±0.7	=
MUFA/PUFA	5.4±0.6	<	7.6±0.8	=	6.6±0.9	=	6.5±1.3	=	6.1±0.3	=	5.0±1.1	=	3.4±0.2	<	5.9±0.1	>	4.9±0.2	=	5.1±0.2	>	4.5±0.2	>	2.8±0.4	<	3.3±0.6	2.8±0.3	5.3±0.3	=	4.7±0.2	=	3.3±0.5	=	3.0±0.1	=
PUFA/ MUFA	0.1±0.0	=	0.1±0.0	=	0.1±0.0	=	0.1±0.1	=	0.1±0.0	=	0.1±0.0	=	0.1±0.0	=	+	<	0.1±0.0	<	0.6±0.4	>	0.1±0.0	=	0.2±0.1	=	0.2±0.1	0.3±0.2	0.1±0.0	=	0.1±0.0	=	0.3±0.3	=	0.8±0.9	=
PUFA/PUFA	63.8±0.4	<	68.9±1.1	=	68.1±2.7	=	66.8±2.0	>	63.6±0.7	>	57.7±2.6	<	69.0±1.0	<	71.8±1.5	=	72.4±1.2	>	69.6±0.1	>	68.4±0.7	>	58.3±0.4	<	68.7±0.2	58.0±4.8	60.2±1.0	=	59.0±0.9	=	65.7±0.7	<	58.3±1.4	=

PC	The lower part of the blades of infected *U. pinnatifida* samples												The upper intact part of the blades of infected *U. pinnatifida* samples												The upper part of the blades of infected U. pinnatifida samples with endophyte		The lower part of the blades of uninfected *U. pinnatifida* samples				The upper part of the blades of uninfected *U. pinnatifida* samples			
	Nov.		Dec.		Jan.		Feb.		Apr.		Jun.	T	Nov.		Dec.		Jan.		Feb.		Apr.		Jun.	T	Nov.	Jun.	Nov.	S	Jun.	S	Nov.	S	Jun.	S
14:1/14:0	+	>	-	=	+	=	+	>	-	<	+	=	-	=	-	=	-	=	-	<	+	<	+	>	+	-	-	=	-	=	+	=	-	=
14:0/14:0	0.1±0.0	>	+	>	+	=	+	=	+	<	0.1±0.1	>	+	>	+	=	+	=	+	=	+	<	+	=	0.1±0.0	+	0.1±0.0	=	0.1±0.1	=	+	=	0.1±0.0	=
14:0/16:4	+	=	+	=	+	=	+	>	-	=	-	<	+	=	+	=	+	>	+	>	-	=	-	<	+	-	+	=	+	=	+	=	-	=
16:3/14:0	+	=	+	=	+	=	+	=	+	=	+	=	+	=	+	=	+	=	+	=	+	>	-	<	+	+	+	=	+	=	+	=	-	=
14:0/16:2	0.1±0.0	=	+	<	0.1±0.0	=	0.1±0.0	=	0.1±0.0	>	+	=	0.1±0.0	=	0.1±0.0	=	0.1±0.0	=	+	=	0.1±0.0	=	+	=	+	+	0.1±0.0	=	0.1±0.0	=	0.1±0.0	=	0.1±0.1	=
14:0/16:1	0.1±0.0	>	0.1±0.0	=	0.1±0.0	>	0.1±0.0	=	0.1±0.1	=	0.1±0.1	=	0.1±0.1	=	0.1±0.0	=	0.1±0.0	=	+	=	0.1±0.0	=	0.1±0.0	=	0.1±0.1	0.1±0.1	0.2±0.0	=	0.1±0.0	=	0.1±0.0	=	0.1±0.0	=
14:0/16:1	+	=	+	<	0.1±0.0	=	0.1±0.0	=	+	=	0.1±0.1	=	+	=	0.1±0.0	=	+	=	+	=	+	=	0.1±0.0	=	0.1±0.0	0.1±0.0	+	=	0.1±0.1	=	0.1±0.0	=	0.1±0.1	=
14:0/16:0	0.1±0.0	>	+	=	+	=	+	=	+	<	0.3±0.1	>	0.1±0.0	>	+	=	+	>	+	<	+	<	0.2±0.1	>	0.1±0.0	0.1±0.1	0.2±0.0	=	0.4±0.1	=	0.1±0.0	=	0.2±0.0	=
14:1/18:4	+	=	+	=	+	=	+	>	+	=	+	<	0.1±0.0	=	0.1±0.0	>	+	=	+	=	+	=	+	=	0.1±0.0	0.1±0.0	+	=	+	=	+	=	+	=
14:0/18:4	0.3±0.0	>	0.2±0.0	=	0.2±0.0	>	0.2±0.0	=	0.2±0.0	=	0.1±0.1	=	0.5±0.1	=	0.4±0.1	=	0.4±0.0	>	0.2±0.0	=	0.2±0.0	<	0.5±0.1	>	0.9±0.2	0.9±0.3	0.5±0.0	>	0.3±0.1	=	0.7±0.1	=	0.5±0.1	=
14:0/18:3	2.9±0.2	>	1.3±0.3	=	1.3±0.1	>	1.0±0.2	<	1.4±0.1	=	1.6±0.3	>	3.0±0.4	>	1.9±0.1	>	1.7±0.0	>	0.8±0.0	<	1.2±0.1	<	1.7±0.0	>	2.1±0.2	1.7±0.1	3.1±0.1	=	1.9±0.0	=	2.3±0.3	=	1.8±0.0	=
14:0/18:2	2.5±0.2	>	1.6±0.4	=	1.6±0.1	=	1.7±0.1	<	2.5±0.1	<	3.8±0.8	>	3.4±0.3	>	2.4±0.4	=	2.0±0.1	=	1.8±0.3	<	2.5±0.3	<	3.3±0.6	>	1.9±0.2	2.2±0.1	3.1±0.5	=	4.5±0.8	=	2.5±0.2	=	4.1±1.7	=
14:0/18:1	0.7±0.1	>	0.4±0.1	=	0.3±0.1	=	0.4±0.2	=	0.4±0.1	<	0.8±0.2	>	0.6±0.1	>	0.3±0.1	=	0.2±0.1	<	0.3±0.0	=	0.3±0.1	=	0.4±0.2	=	0.4±0.0	0.5±0.1	0.8±0.1	=	0.7±0.3	=	0.6±0.0	=	0.6±0.1	=
16:0/16:1	0.2±0.0	>	0.1±0.0	=	0.1±0.0	>	+	<	0.1±0.0	=	0.2±0.1	>	0.2±0.0	>	0.1±0.0	=	0.1±0.1	=	0.1±0.0	=	0.1±0.0	=	0.1±0.1	=	0.1±0.0	0.1±0.0	0.3±0.0	=	0.2±0.1	=	0.1±0.0	=	0.1±0.0	=
16:0/16:0	0.1±0.0	=	0.1±0.1	=	+	>	+	<	+	<	0.1±0.0	>	0.1±0.0	>	+	=	+	=	+	=	+	<	0.1±0.0	=	+	0.1±0.0	0.1±0.0	=	0.2±0.2	=	+	=	0.1±0.0	=
15:1/18:4	+	=	+	=	+	=	+	=	+	=	+	=	+	=	+	>	+	=	+	=	+	=	+	=	+	+	+	=	+	=	+	=	+	=
17:3/16:1	0.1±0.0	>	+	=	+	=	+	=	+	=	+	=	0.1±0.0	=	0.1±0.0	=	0.1±0.0	=	+	=	+	<	0.1±0.0	>	0.2±0.1	0.2±0.1	0.1±0.0	=	+	=	0.1±0.0	=	0.1±0.0	=
15:0/18:3	0.1±0.0	=	0.1±0.0	=	0.1±0.0	=	0.1±0.0	=	0.1±0.0	=	0.1±0.0	=	0.2±0.0	=	0.1±0.0	=	0.1±0.0	=	0.1±0.1	=	0.1±0.0	=	0.1±0.1	=	0.1±0.0	0.2±0.1	0.1±0.0	=	0.1±0.0	=	0.2±0.1	=	0.2±0.0	=
15:0/18:2	0.1±0.0	>	0.1±0.0	=	0.1±0.0	=	0.1±0.1	=	0.1±0.0	<	0.2±0.1	>	0.3±0.0	=	0.2±0.0	=	0.2±0.0	=	0.2±0.0	=	0.2±0.0	<	0.4±0.1	>	0.2±0.0	0.3±0.1	0.2±0.0	=	0.2±0.1	=	0.2±0.1	=	0.4±0.1	=
15:0/18:1	+	=	+	=	+	=	+	=	+	=	+	=	+	=	+	=	+	=	+	=	+	=	0.1±0.0	>	+	+	+	=	0.1±0.1	=	+	=	+	=
15:0/18:1	+	=	+	>	+	<	+	=	+	=	+	=	+	=	+	=	+	=	+	<	+	>	-	<	+	+	+	=	+	=	+	=	-	=
16:3/18:3	+	<	0.1±0.0	=	0.1±0.0	=	0.1±0.0	=	+	>	+	<	0.1±0.0	<	0.1±0.0	=	0.1±0.0	>	+	=	+	>	-	=	+	+	+	=	-	=	+	=	+	=
14:0/20:5	4.4±0.5	<	7.1±0.1	<	7.5±0.2	=	6.5±1.1	=	7.7±1.1	>	1.6±0.4	<	7.0±0.9	=	7.9±0.6	>	6.4±0.5	>	3.8±0.6	<	5.7±0.3	=	5.0±2.1	=	7.4±0.3	6.9±1.1	5.0±0.4	=	1.0±0.1	=	7.1±0.4	=	4.3±0.4	=
14:0/20:4	1.2±0.4	>	0.5±0.1	=	0.4±0.1	<	0.7±0.2	<	1.0±0.0	>	0.4±0.2	=	2.0±0.1	>	0.7±0.1	=	0.6±0.1	=	0.6±0.2	=	0.7±0.3	<	1.1±0.2	>	2.3±0.1	1.1±0.1	1.5±0.2	=	0.2±0.0	=	2.6±0.1	>	1.1±0.1	=
14:0/20:4	6.9±1.2	>	3.5±0.5	=	3.9±0.2	=	4.2±1.0	<	5.7±0.5	<	8.1±0.8	>	4.9±0.2	>	3.5±0.2	>	3.2±0.1	=	3.0±0.2	=	3.3±0.6	<	7.5±1.0	>	4.3±0.1	5.8±0.5	7.3±0.3	=	7.5±0.2	=	4.9±0.3	=	6.8±2.0	=
14:0/20:3	1.2±0.2	>	0.7±0.1	=	0.7±0.1	=	0.6±0.0	<	1.0±0.2	<	1.5±0.3	>	0.8±0.1	>	0.6±0.1	=	0.6±0.0	>	0.4±0.0	=	0.4±0.0	<	1.4±0.2	>	0.8±0.1	1.2±0.4	1.3±0.2	=	1.4±0.2	=	0.8±0.1	=	1.4±0.6	=
16:0/18:3	8.6±0.2	>	4.0±0.9	=	4.3±0.2	<	4.7±0.3	<	5.4±0.5	=	6.6±1.6	=	6.0±0.5	=	5.4±0.3	<	6.1±0.5	=	5.6±0.6	=	5.3±0.2	>	4.1±0.6	<	5.7±0.5	4.3±0.1	8.1±0.2	=	7.4±0.2	=	5.6±0.5	=	4.5±0.3	=
18:0/16:3	0.3±0.1	=	0.3±0.1	=	0.2±0.1	=	0.2±0.1	=	0.4±0.2	=	0.3±0.2	=	0.2±0.1	=	0.3±0.2	=	0.4±0.0	=	0.3±0.1	=	0.3±0.0	=	0.2±0.1	=	0.2±0.1	0.2±0.0	0.6±0.3	=	0.4±0.2	=	0.3±0.1	=	0.4±0.0	=
16:0/18:2	4.7±0.3	=	4.3±0.7	=	4.4±0.3	=	4.5±1.1	=	5.3±0.4	<	9.0±0.8	>	6.8±0.5	>	5.4±0.7	<	6.5±0.3	<	8.3±1.0	=	8.8±1.3	=	8.8±2.0	=	5.6±0.8	6.4±1.3	4.7±0.5	=	10.6±1.5	=	5.8±0.3	=	9.7±1.5	=
16:0/18:1	1.3±0.4	>	0.7±0.2	>	0.3±0.0	=	0.2±0.1	<	0.5±0.1	<	1.4±0.2	>	1.2±0.2	>	0.3±0.1	=	0.4±0.1	=	0.3±0.0	=	0.3±0.1	<	1.1±0.3	>	1.1±0.1	1.2±0.3	1.2±0.2	=	1.7±0.1	=	1.2±0.0	=	1.3±0.2	=
16:0/18:0	+	=	+	>	+	=	+	=	+	<	+	>	-	<	+	=	+	>	-	=	-	<	+	>	+	+	-	=	+	=	+	=	+	=
18:4/17:2	0.1±0.0	<	0.1±0.0	=	0.1±0.0	=	0.1±0.0	=	0.1±0.0	>	+	<	0.1±0.0	=	0.1±0.0	>	0.1±0.0	>	+	<	0.1±0.0	=	0.1±0.1	=	0.1±0.0	0.1±0.0	0.1±0.0	=	+	=	0.1±0.0	=	+	=
15:0/20:5	0.1±0.0	<	0.3±0.0	=	0.3±0.1	=	0.2±0.1	=	0.2±0.0	>	0.2±0.1	=	0.3±0.0	=	0.3±0.0	=	0.4±0.0	>	0.2±0.0	<	0.4±0.0	<	0.5±0.1	>	0.4±0.1	0.7±0.2	0.2±0.1	=	0.1±0.0	=	0.5±0.0	>	0.5±0.2	=
15:0/20:4	-	=	-	=	-	<	0.1±0.0	>	+	>	-	<	-	=	-	=	-	<	0.1±0.0	=	0.1±0.0	>	-	<	-	-	-	=	-	=	-	=	-	=
15:0/20:4	0.3±0.0	>	0.2±0.0	=	0.2±0.0	=	0.1±0.1	=	0.2±0.0	<	0.4±0.2	>	0.4±0.1	>	0.2±0.0	=	0.3±0.0	>	0.2±0.0	=	0.2±0.1	<	0.6±0.1	>	0.5±0.1	0.7±0.1	0.4±0.1	=	0.4±0.0	=	0.5±0.0	=	0.7±0.3	=
17:1/18:2	0.2±0.0	>	0.1±0.0	=	0.1±0.0	<	0.1±0.0	=	0.2±0.0	=	0.2±0.1	>	0.2±0.0	>	0.1±0.0	=	0.2±0.0	>	0.1±0.0	<	0.1±0.0	=	0.2±0.1	=	0.2±0.0	0.2±0.1	0.2±0.0	=	0.2±0.0	=	0.2±0.1	=	0.2±0.0	=

17:0/18:2	0.1±0.0 =	0.1±0.0 >	+ >	+ =	+ <	0.2±0.0 >	0.2±0.0 =	0.1±0.0 >	0.1±0.0 =	0.1±0.0 =	0.1±0.0 =	0.2±0.1 =	0.2±0.1	0.1±0.1	0.1±0.0 =	0.2±0.0 =	0.2±0.0 =	0.2±0.1 =
16:0/19:1	0.1±0.1 >	+ =	+ =	+ =	+ <	+ =	0.1±0.0 >	+ =	+ =	+ >	- <	+ =	0.4±0.6	+	+ =	0.1±0.0 =	+ =	+ =
18:4/18:4	0.1±0.0 <	0.1±0.0 <	0.2±0.0 >	+ <	0.1±0.0 >	+ =	+ <	0.1±0.0 =	0.1±0.0 =	0.1±0.0 =	+ =	0.1±0.0 =	0.1±0.1	0.1±0.0	0.1±0.0 =	+ =	0.1±0.0 =	+ =
18:3/18:4	0.3±0.1 <	0.4±0.1 <	0.5±0.0 >	0.3±0.1 =	0.3±0.0 >	0.1±0.0 <	0.2±0.0 <	0.3±0.1 =	0.3±0.0 >	0.2±0.1 =	0.2±0.0 >	0.2±0.0 =	0.3±0.1	0.2±0.1	0.3±0.0 =	0.1±0.0 =	0.2±0.0 =	0.2±0.0 =
18:3/18:3	1.2±0.1 <	1.9±0.2 =	1.7±0.3 =	1.2±0.2 >	0.9±0.0 >	0.5±0.2 <	0.8±0.2 <	1.3±0.2 =	1.2±0.1 >	0.9±0.0 <	1.0±0.0 >	0.4±0.1 <	0.8±0.3	0.7±0.2	1.2±0.1 =	0.3±0.0 =	0.7±0.1 =	0.4±0.1 =
18:3/18:2	0.6±0.3 =	0.8±0.1 >	0.4±0.1 =	0.3±0.1 =	0.2±0.1 =	0.4±0.3 =	0.2±0.0 <	0.5±0.2 =	0.4±0.1 >	0.2±0.0 =	0.2±0.1 =	0.2±0.1 =	0.2±0.1	0.2±0.1	0.5±0.3 =	0.4±0.2 =	0.2±0.0 =	0.1±0.1 =
18:2/18:3	0.6±0.1 >	0.3±0.0 >	0.2±0.0 =	0.3±0.1 =	0.3±0.1 =	0.3±0.1 =	0.3±0.1 =	0.3±0.1 =	0.2±0.0 >	0.1±0.1 <	0.4±0.1 =	0.2±0.1 =	0.3±0.1	0.2±0.1	0.6±0.2 =	0.3±0.0 =	0.2±0.0 =	0.2±0.0 =
20:4/16:1	0.2±0.2 =	0.2±0.0 =	0.1±0.0 =	0.2±0.1 =	0.2±0.1 =	0.3±0.0 =	0.1±0.0 <	0.2±0.0 >	0.1±0.1 <	0.2±0.1 =	0.2±0.1 =	0.2±0.0 =	0.1±0.0	0.1±0.0	0.3±0.0 =	0.2±0.1 =	0.2±0.0 =	0.1±0.0 <
16:0/20:5	7.2±1.3 <	16.4±1.6 <	21.2±1.1 >	18.8±1.0 >	15.3±0.7 >	3.4±1.5 <	12.1±1.4 <	18.5±0.6 =	20.8±2.0 =	18.9±1.6 =	19.1±1.0 >	10.2±2.6 <	14.7±2.1	14.5±1.9	8.1±0.1 =	2.1±0.4 =	14.0±0.4 =	8.0±3.4 =
16:0/20:4	2.1±0.4 >	0.8±0.2 <	1.3±0.1 =	1.8±0.7 =	2.0±0.2 >	0.7±0.2 <	3.3±0.1 >	1.4±0.1 <	1.8±0.1 =	2.6±0.8 =	2.1±0.7 =	2.3±0.3 =	4.4±1.0	3.6±1.7	1.9±0.1 =	1.0±0.4 =	4.6±0.1 >	1.9±1.4 =
16:0/20:4	9.6±0.9 >	6.7±0.7 =	7.5±0.5 =	9.0±1.4 =	9.0±1.0 <	15.4±1.3 >	7.1±0.1 >	5.9±0.4 =	5.9±0.1 <	9.5±1.8 =	7.6±0.7 <	12.2±1.9 =	5.7±0.4	9.6±0.5	9.2±1.5 =	14.9±0.2 =	6.8±0.2 =	12.5±0.1 =
16:0/20:3	6.0±0.4 >	1.8±0.2 <	2.4±0.2 <	4.1±0.0 <	4.4±0.0 <	6.4±1.2 >	3.7±0.3 >	2.1±0.1 <	2.7±0.1 <	4.1±0.6 >	2.6±0.2 <	3.1±0.3 <	2.7±0.2	2.7±0.3	5.3±0.2 =	7.1±0.9 =	3.2±0.1 =	3.2±0.3 =
18:0/18:2	0.6±0.2 =	0.7±0.7 =	0.2±0.1 <	0.3±0.0 =	0.4±0.1 <	1.4±0.3 >	1.1±0.2 >	0.4±0.1 =	0.4±0.0 <	0.6±0.0 =	0.8±0.2 <	1.8±0.2 >	0.9±0.2	0.9±0.2	0.5±0.0 =	1.6±0.4 =	0.9±0.2 =	2.1±0.2 =
18:0/18:1	0.1±0.1 =	0.1±0.1 =	+ =	+ =	+ <	0.2±0.0 >	0.1±0.0 =	+ =	+ =	+ =	+ =	0.1±0.1 =	0.1±0.0	0.1±0.0	0.1±0.1 =	0.2±0.1 =	0.1±0.0 =	0.1±0.0 =
18:0/18:0	+ =	+ >	+ =	+ =	+ <	+ >	+ >	+ =	+ <	+ =	+ =	+ =	-	+	+ =	+ =	+ =	- =
20:5/18:5	+ =	0.1±0.0 =	0.1±0.0 >	- =	+ =	+ =	+ =	+ =	+ =	0.1±0.0 =	0.1±0.0 >	+ =	0.1±0.0	+	+ =	- =	+ =	+ =
20:5/18:4	0.3±0.1 <	0.5±0.0 =	0.5±0.0 >	0.3±0.1 =	0.2±0.0 >	0.1±0.0 <	0.3±0.0 <	0.6±0.2 =	0.7±0.1 >	0.4±0.1 =	0.3±0.0 >	0.2±0.0 <	0.9±0.2	0.7±0.4	0.3±0.0 =	0.1±0.1 =	0.6±0.1 =	0.2±0.1 =
20:5/18:3	2.0±0.2 <	5.0±0.4 >	4.0±0.6 >	2.3±0.1 =	2.1±0.2 >	0.8±0.3 <	1.7±0.2 <	3.1±0.3 =	3.2±0.2 >	2.0±0.4 =	1.9±0.2 >	1.0±0.0 <	2.6±0.5	2.4±0.5	2.1±0.1 =	0.5±0.1 =	1.7±0.2 =	1.0±0.3 =
20:4/18:3	4.5±0.4 <	6.5±0.6 >	4.5±0.4 =	4.3±0.3 >	3.8±0.1 =	2.9±0.9 <	3.3±0.1 <	5.1±0.3 =	4.9±0.3 >	4.0±0.3 =	4.2±0.2 >	2.0±0.5 <	3.0±0.4	2.6±0.3	4.3±0.1 =	3.0±1.0 =	2.9±0.2 =	2.3±0.1 =
20:5/18:2	0.4±0.1 =	0.4±0.1 =	0.5±0.0 =	0.3±0.1 =	0.2±0.1 =	0.3±0.1 =	0.2±0.0 =	0.3±0.0 =	0.3±0.0 =	0.3±0.1 =	0.3±0.0 =	0.4±0.4 =	0.3±0.1	0.3±0.1	0.5±0.0 =	0.3±0.1 =	0.2±0.0 =	0.4±0.2 =
20:4/18:2	3.8±0.3 >	2.9±0.3 >	2.1±0.2 <	2.6±0.3 =	2.7±0.2 <	5.2±1.0 >	3.3±0.5 =	2.8±0.2 >	1.9±0.3 <	3.0±0.4 =	3.4±0.3 =	3.8±1.0 =	2.5±0.2	2.3±0.4	3.8±0.4 =	6.5±0.2 =	2.2±0.1 =	4.3±1.4 =
18:1/20:5	0.5±0.0 >	0.2±0.0 =	0.2±0.0 =	0.2±0.0 =	0.1±0.1 <	0.6±0.3 >	0.3±0.2 =	0.2±0.0 =	0.2±0.1 =	0.4±0.1 >	0.2±0.0 <	0.3±0.0 =	0.3±0.1	0.3±0.1	0.3±0.1 =	0.5±0.1 =	0.2±0.0 =	0.4±0.4 =
20:3/18:2	0.3±0.1 >	0.2±0.1 =	0.1±0.0 =	0.2±0.1 =	0.2±0.0 =	0.3±0.1 =	0.2±0.1 =	0.2±0.0 =	0.2±0.0 =	0.2±0.0 =	0.2±0.0 =	0.2±0.1 =	0.1±0.0	0.1±0.0	0.2±0.0 <	0.5±0.3 =	0.1±0.1 =	0.1±0.0 =
18:2/20:3	0.3±0.1 >	+ =	+ <	0.1±0.0 =	0.1±0.0 =	0.2±0.2 =	0.2±0.1 =	+ =	+ =	0.1±0.1 =	0.1±0.0 =	0.1±0.0 =	0.1±0.1	0.1±0.0	- =	0.2±0.2 =	+ =	0.1±0.1 =
20:4/18:1	0.3±0.1 >	0.2±0.1 >	0.1±0.0 =	0.1±0.0 =	0.2±0.1 <	0.5±0.2 >	0.2±0.1 =	0.1±0.1 =	0.1±0.0 =	0.1±0.0 =	0.1±0.0 <	0.2±0.1 >	0.2±0.1	0.2±0.0	0.5±0.0 =	0.6±0.0 =	0.3±0.1 =	0.2±0.1 =
18:0/20:5	1.2±0.2 <	1.6±0.1 >	0.9±0.1 =	1.2±0.3 =	1.3±0.0 >	0.8±0.3 =	2.0±0.3 >	1.6±0.1 =	1.5±0.2 =	1.2±0.2 =	1.4±0.4 =	1.8±0.5 =	1.9±0.3	1.7±0.0	1.2±0.1 =	0.5±0.0 =	1.9±0.1 =	1.7±0.5 =
18:0/20:4	0.2±0.1 =	0.1±0.0 =	+ <	0.1±0.0 =	0.1±0.0 >	+ <	0.4±0.1 >	0.1±0.0 =	0.1±0.0 =	0.1±0.1 =	0.1±0.0 <	0.3±0.0 >	0.6±0.0	0.2±0.1	0.1±0.1 =	0.4±0.5 =	0.4±0.1 =	0.4±0.1 =
18:0/20:4	1.4±0.1 =	0.9±0.8 =	0.3±0.0 <	0.4±0.1 <	0.6±0.1 <	3.1±0.7 >	0.9±0.0 >	0.4±0.2 =	0.4±0.0 =	0.6±0.2 =	0.5±0.2 <	1.8±0.2 >	0.9±0.2	1.1±0.3	1.3±0.1 =	2.4±0.6 =	0.9±0.1 =	2.1±0.6 =
18:0/20:3	0.4±0.0 >	0.1±0.0 =	+ <	0.1±0.0 <	0.1±0.0 =	0.5±0.3 >	0.2±0.1 =	0.1±0.1 =	0.2±0.1 =	0.2±0.0 =	0.1±0.1 =	0.2±0.1 =	0.1±0.0	0.2±0.1	0.2±0.0 <	0.6±0.1 =	0.2±0.2 =	0.5±0.2 =
20:0/18:3	0.3±0.1 =	0.1±0.1 =	0.1±0.0 <	0.1±0.0 =	0.1±0.1 <	0.6±0.3 >	0.3±0.2 =	0.2±0.1 =	0.2±0.1 =	0.1±0.0 =	0.1±0.0 =	0.3±0.2 =	0.3±0.1	0.1±0.1	0.3±0.0 =	0.5±0.2 =	0.3±0.2 =	0.1±0.1 =
20:0/18:2	0.1±0.0 =	0.1±0.1 =	+ =	+ =	+ <	0.3±0.2 >	0.2±0.0 >	0.1±0.1 =	0.1±0.0 =	0.2±0.0 <	0.2±0.0 <	0.6±0.1 >	0.3±0.1	0.3±0.1	0.1±0.0 =	0.4±0.1 =	0.3±0.0 =	0.6±0.2 =
16:0/22:1	+ =	+ =	+ =	+ =	+ =	+ >	+ =	+ =	+ <	+ =	+ =	+ =	+	+	+ =	- =	+ =	+ =
20:0/18:0	- <	+ >	- <	+ =	+ =	+ =	- <	+ =	+ <	+ >	- <	+ >	-	+	- =	- =	- =	- =
20:5/20:5	1.8±0.5 <	10.1±1.2 =	10.3±0.4 >	6.6±0.4 >	4.8±0.3 >	0.3±0.3 <	3.3±0.3 <	8.1±0.2 =	8.5±0.4 >	5.1±0.9 =	5.4±0.6 >	1.2±0.6 <	5.7±0.4	3.9±0.5	2.1±0.1 =	0.2±0.1 =	4.2±0.5 =	1.2±0.7 =
20:5/20:4	0.6±0.2 <	1.0±0.2 =	0.8±0.1 =	0.7±0.3 =	0.9±0.2 >	0.1±0.0 <	0.8±0.3 =	0.9±0.1 =	0.9±0.1 >	0.7±0.1 <	0.9±0.1 >	0.5±0.1 =	1.7±0.3	1.2±0.1	0.4±0.1 =	0.1±0.1 =	1.6±0.6 =	0.3±0.3 =
20:4/20:5	5.4±0.7 <	8.4±1.5 =	7.9±0.7 <	9.0±0.0 >	7.8±0.3 >	2.5±0.8 <	7.0±0.2 <	8.0±0.6 =	7.4±0.5 <	8.5±0.4 =	8.9±0.8 >	5.3±0.5 <	6.4±0.7	5.5±0.9	5.7±0.1 =	1.4±0.2 =	6.7±0.4 =	4.8±0.1 =
20:4/20:4	1.7±0.4 >	1.1±0.1 =	1.1±0.1 <	1.8±0.5 =	1.7±0.1 >	0.8±0.4 <	2.0±0.1 >	1.2±0.1 =	1.2±0.1 <	1.7±0.3 =	1.5±0.3 =	1.2±0.2 <	1.7±0.3	1.5±0.3	1.9±0.2 =	0.5±0.2 =	2.0±0.1 =	1.3±0.1 =
20:4/20:4	5.4±1.0 >	2.6±0.5 =	2.5±0.2 <	3.9±0.4 =	3.9±0.3 <	8.7±1.2 >	2.9±0.4 >	2.2±0.3 =	1.8±0.4 <	3.7±0.9 =	2.8±0.3 <	6.3±1.6 >	1.9±0.3	3.9±1.0	4.5±0.6 =	7.6±0.8 =	2.0±0.2 =	6.3±2.6 =
20:4/20:3	2.4±0.4 >	0.7±0.2 =	0.7±0.0 <	1.6±0.3 =	1.7±0.1 <	3.0±0.6 >	1.1±0.2 >	0.8±0.1 =	0.8±0.2 <	1.4±0.2 >	0.9±0.1 <	1.3±0.2 =	0.9±0.1	1.1±0.4	2.0±0.3 =	3.2±0.4 =	1.0±0.0 =	1.3±0.6 =
20:4/20:2	0.2±0.1 >	0.1±0.0 =	0.1±0.0 =	0.1±0.1 =	0.1±0.0 <	0.3±0.0 =	0.1±0.1 <	0.2±0.0 =	0.2±0.1 =	0.2±0.0 =	0.2±0.1 =	0.2±0.0 =	0.1±0.0	0.1±0.1	0.2±0.0 =	0.2±0.1 =	0.1±0.0 =	0.2±0.0 =
20:4/20:2	0.1±0.0 =	0.1±0.0 =	0.1±0.0 =	0.1±0.1 =	0.1±0.0 <	0.3±0.1 >	0.1±0.1 =	0.1±0.1 =	0.1±0.1 =	0.1±0.0 =	0.2±0.1 <	0.4±0.1 >	0.1±0.0	0.2±0.1	0.1±0.0 =	0.2±0.2 =	0.1±0.0 =	0.3±0.1 =
20:0/20:5	0.5±0.1 <	0.9±0.1 >	0.7±0.1 <	0.9±0.2 >	0.7±0.1 >	0.3±0.1 <	0.8±0.1 <	1.4±0.3 =	1.1±0.1 =	1.0±0.1 =	1.3±0.2 >	0.8±0.2 =	0.9±0.1	0.8±0.1	0.6±0.1 =	0.2±0.0 =	1.1±0.2 =	0.8±0.4 =
20:0/20:4	0.4±0.1 >	0.1±0.0 =	0.1±0.0 <	0.3±0.0 =	0.3±0.0 <	1.0±0.2 >	0.4±0.1 =	0.3±0.1 =	0.2±0.0 <	0.4±0.1 =	0.3±0.0 <	0.9±0.3 >	0.5±0.1	0.6±0.2	0.4±0.1 =	1.0±0.1 =	0.5±0.1 =	0.8±0.1 =

22:1/18:2	+	=	+	=	+	=	+	=	+	<	+	=	+	=	+	=	+	=	+	=	+	<	+	>	0.1±0.1	+	+	=	0.1±0.1	=	+	=	+	=
20:5/22:2	-	=	-	=	-	=	-	<	+	>	-	=	+	>	-	=	-	<	+	=	+	<	+	>	-	+	+	=	-	=	-	=	-	=
20:4/22:2	-	=	+	=	+	=	+	>	-	=	-	<	+	<	+	>	+	=	+	=	+	>	-	<	-	-	-	=	-	=	-	=	-	=
SFA/SFA	0.3±0.0	>	0.1±0.1	=	0.1±0.0	=	0.1±0.0	=	0.1±0.0	<	0.6±0.1	>	0.2±0.0	>	0.1±0.0	=	+	=	+	=	0.1±0.0	<	0.3±0.1	>	0.2±0.0	0.2±0.1	0.3±0.0	=	0.7±0.4	=	0.2±0.0	=	0.4±0.0	=
SFA/MUFA	2.5±0.6	>	1.3±0.2	>	0.7±0.1	=	0.8±0.3	=	1.0±0.1	<	2.7±0.3	>	2.2±0.4	>	0.9±0.1	=	0.7±0.2	=	0.7±0.1	=	0.8±0.1	<	1.8±0.4	>	2.2±0.6	2.0±0.2	2.5±0.4	=	2.9±0.1	=	2.1±0.0	=	2.2±0.1	=
MUFA/SFA	-	=	-	=	-	=	-	=	-	=	-	=	-	=	-	=	-	=	-	=	-	=	-	=	-	-	-	=	-	=	-	=	-	=
SFA/PUFA	63.7±1.1	>	54.5±2.7	<	60.1±0.6	<	62.4±1.1	<	65.7±1.2	<	67.7±0.8	>	68.4±0.8	>	62.1±1.4	=	64.2±1.7	=	65.2±0.7	=	65.6±1.0	<	71.7±1.1	>	66.4±1.8	69.0±2.9	65.3±0.1	=	69.0±3.1	=	69.5±1.1	=	71.3±3.5	=
PUFA/SFA	+	=	+	=	+	>	+	=	+	>	+	=	+	=	+	=	+	>	+	=	+	>	+	=	+	+	+	=	+	=	+	=	+	=
MUFA/MUFA	-	=	-	=	-	=	-	=	-	=	-	=	-	=	-	=	-	=	-	=	-	=	-	=	-	-	-	=	-	=	-	=	-	=
MUFA/PUFA	0.8±0.0	>	0.3±0.1	=	0.3±0.1	=	0.3±0.1	=	0.3±0.2	<	0.8±0.2	>	0.6±0.2	=	0.4±0.0	=	0.5±0.1	=	0.5±0.1	>	0.3±0.1	<	0.5±0.1	=	0.6±0.0	0.7±0.0	0.6±0.1	=	0.9±0.2	=	0.5±0.0	=	0.7±0.3	=
PUFA/ MUFA	0.6±0.1	>	0.4±0.0	>	0.3±0.0	=	0.4±0.1	=	0.4±0.0	<	0.8±0.2	>	0.3±0.1	=	0.4±0.1	>	0.2±0.0	<	0.3±0.1	=	0.3±0.0	<	0.5±0.1	>	0.5±0.1	0.5±0.1	0.7±0.2	=	0.8±0.1	=	0.5±0.1	=	0.4±0.0	=
PUFA/PUFA	31.9±1.0	<	43.2±2.8	>	38.4±0.5	>	36.0±1.3	>	32.3±1.1	>	27.1±1.2	<	28.1±0.6	<	36.0±1.2	=	34.3±1.6	=	33.0±0.8	=	32.9±1.0	>	25.0±1.3	<	30.0±1.2	27.6±3.3	30.3±0.5	=	25.5±3.2	=	27.1±1.2	=	24.9±3.2	=

PI	The lower part of the blades of infected *U. pinnatifida* samples												The upper intact part of the blades of infected *U. pinnatifida* samples												The upper part of the blades of infected *U. pinnatifida* samples with endophyte		The lower part of the blades of uninfected *U. pinnatifida* samples				The upper part of the blades of uninfected *U. pinnatifida* samples			
	Nov.		Dec.		Jan.		Feb.		Apr.		Jun.	T	Nov.		Dec.		Jan.		Feb.		Apr.		Jun.	T	Nov.	Jun.	Nov.	S	Jun.	S	Nov.	S	Jun.	S
14:0/18:4	0.1±0.2	=	+	=	+	=	+	=	0.1±0.2	=	+	=	+	=	+	=	+	=	+	=	+	=	+	=	+	+	+	=	+	=	+	=	+	
18:3/14:0	0.3±0.4	=	0.6±0.7	=	0.6±0.8	=	+	=	+	=	+	=	+	=	+	=	0.2±0.4	=	0.3±0.3	=	+	=	0.2±0.3	=	1.6±2.7	+	0.6±0.8	=	+	=	0.4±0.6	=	0.6±0.8	=
14:0/18:2	0.8±0.5	=	3.7±4.0	=	1.3±1.3	=	0.1±0.2	=	0.3±0.5	=	1.7±1.9	=	2.2±1.3	=	0.4±0.8	=	0.3±0.3	=	0.2±0.2	=	0.2±0.3	=	1.7±2.2	=	0.1±0.2	1.0±1.0	+	=	1.6±2.3	=	1.1±0.3	=	0.1±0.1	=
14:0/18:1	4.0±1.9	=	2.9±0.7	=	1.9±1.4	=	1.3±0.9	=	3.1±1.5	=	4.2±2.8	=	3.6±3.5	=	0.2±0.3	<	3.9±1.0	=	3.3±3.3	=	3.7±2.0	=	2.0±3.2	=	4.4±0.4	5.3±3.2	3.4±0.3	=	4.5±0.4	=	2.3±0.5	=	0.4±0.3	=
16:0/16:0	1.5±1.3	=	0.4±0.6	=	0.3±0.3	=	0.3±0.2	=	0.1±0.2	=	0.8±0.8	=	0.3±0.5	=	0.4±0.6	=	+	=	1.3±1.8	=	0.3±0.6	=	0.2±0.3	=	0.6±0.5	0.7±1.2	0.4±0.6	=	+	=	1.9±1.8	=	0.6±0.8	=
14:0/20:5	+	=	+	=	+	=	+	=	+	=	+	=	+	=	+	=	+	=	0.3±0.5	=	+	=	0.1±0.2	=	+	0.1±0.2	+	=	+	=	+	=	0.5±0.7	=
16:0/18:4	0.2±0.4	=	0.2±0.4	=	0.5±0.4	=	0.3±0.3	>	+	=	0.2±0.4	=	1.5±1.3	>	+	=	0.2±0.3	=	0.3±0.3	=	+	=	0.4±0.4	=	0.3±0.2	0.3±0.5	1.6±2.3	=	2.0±2.8	=	+	=	0.2±0.3	=
16:0/18:3	2.9±2.8	=	4.9±2.3	=	6.1±1.6	>	2.0±1.0	=	2.4±1.5	=	2.9±3.5	=	5.6±3.7	=	1.3±1.2	<	5.7±1.3	=	3.8±1.2	=	6.3±2.8	=	10.0±2.6	>	9.5±5.5	8.4±4.3	6.0±0.4	=	7.1±10.1	=	5.7±1.5	=	13.6±6.6	=
16:0/18:2	8.2±2.9	<	14.0±1.8	=	14.4±1.3	<	20.8±5.4	=	16.2±3.0	=	14.4±2.7	=	9.0±2.4	=	18.9±18.4	=	15.4±4.9	=	23.0±4.9	=	26.7±2.7	=	28.0±4.5	=	10.7±3.8	17.8±4.5	7.1±2.7	=	12.5±8.4	=	9.1±3.0	=	25.8±0.3	=
16:0/18:1	66.7±3.1	>	60.4±2.2	=	63.8±3.2	=	65.0±4.5	=	64.7±10.1	=	63.5±8.3	=	61.6±6.7	=	73.1±13.4	=	60.6±0.8	=	54.2±5.6	=	48.8±3.6	>	42.6±3.8	<	59.4±3.1	47.1±6.2	67.3±3.1	=	55.1±5.9	=	67.2±9.6	=	40.2±1.0	=
16:0/18:0	8.4±1.5	=	9.3±3.0	=	9.3±1.4	=	7.6±1.1	=	8.1±2.2	=	9.1±3.7	=	8.7±0.3	=	13.0±5.8	=	7.9±2.2	=	7.5±3.0	=	7.1±2.6	=	7.1±5.0	=	8.7±2.3	10.7±5.6	8.2±0.8	=	3.7±2.0	=	7.6±4.2	=	5.5±1.1	=
17:0/18:4	0.6±1.0	=	0.5±0.9	=	0.3±0.4	=	0.3±0.3	=	0.4±0.3	=	0.3±0.4	=	+	=	+	=	0.1±0.2	=	0.4±0.7	=	+	=	0.5±0.9	=	+	1.0±1.7	+	=	+	=	+	=	+	=
17:0/18:3	0.2±0.4	=	0.4±0.6	=	+	=	0.6±0.7	=	+	=	0.1±0.2	=	+	=	+	=	+	=	0.6±1.0	=	0.1±0.2	=	0.7±0.8	=	+	+	+	=	+	=	0.3±0.4	=	0.3±0.5	=
17:0/18:2	0.2±0.4	=	+	=	0.2±0.3	=	0.6±0.3	=	0.2±0.3	=	0.1±0.2	<	0.2±0.4	=	0.1±0.1	=	0.1±0.2	=	0.1±0.2	=	+	=	1.1±1.3	=	+	0.3±0.5	+	=	+	=	+	=	0.2±0.3	=
18:1/17:0	0.6±0.4	=	0.8±1.1	=	1.4±1.7	=	1.3±0.6	>	0.3±0.5	=	0.4±0.4	<	+	=	0.2±0.3	<	0.8±0.2	=	1.6±2.7	=	0.9±0.9	=	0.4±0.7	=	1.3±0.5	1.0±1.2	0.7±1.0	=	2.0±2.8	=	0.7±1.0	=	0.3±0.4	=
16:0/20:5	0.8±0.7	=	0.6±0.8	=	1.2±0.4	=	1.0±1.2	=	0.8±0.9	=	0.4±0.4	=	0.1±0.3	=	0.3±0.5	=	1.5±1.9	=	+	<	1.2±0.5	=	0.6±0.8	=	0.6±0.8	0.6±1.1	0.8±1.1	=	+	=	0.8±0.2	>	1.3±1.8	=
16:0/20:4	0.2±0.2	<	1.8±1.0	=	0.4±0.4	=	0.5±0.6	=	1.7±0.7	=	0.6±0.5	=	0.4±0.4	=	0.3±0.5	<	1.9±1.0	=	1.4±0.4	=	1.9±0.6	=	0.9±0.9	=	0.5±0.4	4.5±1.8	0.8±1.1	=	7.1±2.5	=	+	=	1.1±1.5	=
20:3/16:0	0.1±0.2	=	1.1±1.0	=	0.9±0.9	=	0.6±0.4	=	1.2±1.3	=	1.6±0.7	>	1.2±0.4	>	0.1±0.1	<	1.7±1.0	=	1.3±1.0	=	1.0±0.9	>	+	<	0.7±0.3	1.1±1.5	0.2±0.3	=	+	=	+	<	1.7±2.4	=
18:1/18:1	2.3±1.0	=	1.0±0.9	=	0.7±0.5	=	0.7±0.7	<	2.5±0.7	=	1.9±1.6	=	0.6±1.0	=	0.3±0.5	=	0.5±0.5	=	1.0±0.6	=	0.8±1.4	=	0.2±0.4	=	1.0±1.4	0.3±0.5	0.6±0.3	=	0.6±0.9	=	0.1±0.2	=	0.1±0.2	=
18:0/18:1	1.9±2.3	=	1.4±1.5	=	2.2±0.8	=	1.4±0.4	=	2.9±1.5	=	3.5±0.7	>	3.7±2.3	=	1.2±2.0	=	1.3±0.5	=	1.4±0.3	=	1.3±1.5	=	0.3±0.3	<	0.7±1.1	0.1±0.2	2.0±0.6	=	1.3±1.8	=	2.8±2.1	=	1.2±1.7	=
18:0/18:0	+	=	+	=	+	=	1.1±1.2	=	0.8±0.3	=	0.8±1.0	=	1.2±2.0	=	1.8±1.9	=	0.1±0.1	=	1.2±1.3	=	0.6±0.5	=	0.7±1.3	=	+	1.9±1.0	0.1±0.2	=	0.6±0.9	=	+	=	0.5±0.1	=
SFA/SFA	9.8±0.6	=	9.6±2.5	=	9.7±1.5	=	8.9±1.8	=	8.7±2.1	=	10.7±3.5	=	10.1±2.5	=	15.2±4.1	>	8.0±2.1	=	9.7±5.8	=	8.1±3.2	=	8.0±5.0	=	9.2±2.8	13.3±4.3	8.8±0.0	=	4.3±2.9	=	9.5±2.5	=	6.6±0.4	=
SFA/MUFA	72.6±1.2	>	64.7±0.5	=	67.9±3.3	=	67.6±5.0	=	70.7±8.9	=	71.2±9.4	=	69.0±9.1	=	74.4±13.4	=	65.8±1.4	>	59.0±3.1	=	53.8±4.1	=	44.9±6.8	<	64.4±2.9	52.4±3.1	72.7±3.4	=	60.8±8.0	=	72.3±8.1	=	41.8±2.3	=

MUFA/SFA	0.6±0.4	=	0.8±1.1	=	1.4±1.7	=	1.3±0.6	=	0.3±0.5	=	0.4±0.4	=	+	=	0.2±0.3	=	0.8±0.2	=	1.6±2.7	=	0.9±0.9	=	0.4±0.7	=	1.3±0.5	1.0±1.2	0.7±1.0	=	2.0±2.8	=	0.7±1.0	=	0.3±0.4	=
SFA/PUFA	14.2±1.6	<	26.2±2.4	=	23.9±2.0	=	26.2±4.6	=	22.2±6.1	=	20.1±5.9	=	19.2±6.3	=	21.3±16.7	=	25.1±1.0	=	30.1±5.2	=	36.3±2.1	=	43.9±7.5	>	21.7±3.9	34.0±0.8	20.1±0.0	=	30.4±4.7	=	16.9±5.1	=	43.1±1.6	=
PUFA/SFA	0.4±0.7	<	1.7±0.4	=	1.4±1.7	=	0.6±0.4	=	1.2±1.3	=	1.6±0.7	>	1.2±0.4	>	0.1±0.1	<	1.9±1.3	=	1.6±0.8	=	1.0±0.9	=	0.2±0.3	<	2.3±2.4	1.1±1.5	0.8±1.1	=	+	=	0.4±0.6	=	2.3±3.2	=
MUFA/MUFA	2.3±1.0	=	1.0±0.9	=	0.7±0.5	=	0.7±0.7	<	2.5±0.7	=	1.9±1.6	=	0.6±1.0	=	0.3±0.5	=	0.5±0.5	=	1.0±0.6	=	0.8±1.4	=	0.2±0.4	=	1.0±1.4	0.3±0.5	0.6±0.3	=	0.6±0.9	=	0.1±0.2	=	0.1±0.2	=
MUFA/PUFA	-	=	-	=	-	=	-	=	-	=	-	=	-	=	-	=	-	=	-	=	-	=	-	=	-	-	-	<	-	<	-	<	-	<
PUFA/ MUFA	-	=	-	=	-	=	-	=	-	=	-	=	-	=	-	=	-	=	-	=	-	=	-	=	-	-	-	<	-	<	-	<	-	<
PUFA/PUFA	-	=	-	=	-	=	-	=	-	=	-	=	-	=	-	=	-	=	-	=	-	=	-	=	-	-	-	<	-	<	-	<	-	<

PHEG	The lower part of the blades of infected *U. pinnatifida* samples												The upper intact part of the blades of infected *U. pinnatifida* samples												The upper part of the blades of infected *U. pinnatifida* samples with endophyte		The lower part of the blades of uninfected *U. pinnatifida* samples				The upper part of the blades of uninfected *U. pinnatifida* samples			
	Nov.		Dec.		Jan.		Feb.		Apr.		Jun.	T	Nov.		Dec.		Jan.		Feb.		Apr.		Jun.	T	Nov.	Jun.	Nov.	S	Jun.	S	Nov.	S	Jun.	S
20:5/20:5	0.4±0.1	<	2.6±0.9	=	2.0±0.4	>	1.3±0.2	>	0.8±0.2	>	+	<	0.4±0.3	<	2.0±0.7	=	1.9±0.2	>	1.0±0.4	>	0.5±0.2	=	0.2±0.1	<	1.6±0.3	2.8±1.0	0.8±0.0	>	0.2±0.2	=	0.7±0.1	=	0.3±0.1	=
20:5/20:4	18.3±1.1	<	33.4±1.4	=	33.0±3.8	>	22.8±2.4	=	20.9±0.6	>	5.6±3.5	<	16.2±1.1	<	25.0±0.8	=	24.7±1.7	>	14.3±1.5	<	19.4±1.3	>	7.9±3.2	<	23.0±3.1	18.6±0.2	19.5±0.8	=	2.9±0.8	=	18.6±3.2	=	7.8±2.3	=
20:4/20:4	80.8±0.9	>	63.4±2.2	=	64.4±4.3	<	74.9±2.2	<	77.6±0.8	<	93.9±3.5	>	83.0±1.0	>	72.3±1.5	=	73.0±1.9	<	83.9±1.7	>	79.6±1.5	<	91.3±2.8	>	75.0±2.9	78.4±1.1	79.2±0.9	=	96.0±1.3	=	80.4±3.3	=	91.5±2.4	=
20:4/20:3	0.5±0.2	=	0.6±0.2	=	0.6±0.1	=	0.9±0.3	=	0.7±0.1	=	0.5±0.2	<	0.4±0.1	<	0.7±0.0	>	0.4±0.1	<	0.8±0.0	>	0.5±0.2	=	0.9±0.4	=	0.3±0.0	0.2±0.1	0.5±0.1	=	0.9±0.3	=	0.3±0.1	=	0.4±0.1	=
20:4/20:2	-	=	-	=	+	>	-	<	+	>	-	=	+	>	-	<	+	>	-	<	+	=	0.1±0.0	>	-	-	+	=	-	=	+	=	0.1±0.0	=

TAG	The lower part of the blades of infected *U. pinnatifida* samples												The upper intact part of the blades of infected *U. pinnatifida* samples												The upper part of the blades of infected *U. pinnatifida* samples with endophyte		The upper part of the blades of uninfected *U. pinnatifida* samples				The lower part of the blades of infected *U. pinnatifida* samples			
	Nov.		Dec.		Jan.		Feb.		Apr.		Jun.	T	Nov.		Dec.		Jan.		Feb.		Apr.		Jun.	T	Nov.	Jun.	Nov.	[illegible]	Jun.	S	Nov.	S	Jun.	S
46:2	0.2±0.0	=	0.2±0.1	=	0.1±0.1	=	+	=	+	<	0.1±0.1	=	0.3±0.4	=	0.3±0.1	>	0.1±0.0	>	+	>	+	<	0.2±0.1	>	0.2±0.0	0.1±0.1	0.2±0.0	[illegible]	+	=	0.4±0.1	=	0.1±0.1	=
47:1	1.3±0.9	=	0.9±0.2	>	0.3±0.1	>	0.1±0.0	=	0.1±0.0	<	0.9±0.5	>	0.9±0.2	=	1.2±0.3	>	0.3±0.0	>	0.1±0.0	=	0.1±0.0	<	0.8±0.3	>	0.7±0.1	0.8±0.4	1.2±0.0	[illegible]	0.5±0.1	=	0.5±0.0	=	0.4±0.0	=
47:2	0.8±0.5	=	0.5±0.0	>	0.1±0.1	>	+	<	0.1±0.0	<	0.5±0.1	>	0.6±0.3	=	0.6±0.2	>	0.1±0.0	>	+	<	0.1±0.0	<	0.5±0.2	>	0.5±0.2	0.4±0.1	0.8±0.0	[illegible]	0.3±0.1	=	0.3±0.0	=	0.2±0.1	=
49:2	1.8±1.0	=	1.2±0.1	>	0.5±0.0	>	0.2±0.0	=	0.2±0.0	<	1.1±0.1	>	1.2±0.3	<	1.6±0.2	>	0.5±0.1	>	0.1±0.0	=	0.1±0.0	<	1.3±0.4	>	1.0±0.5	1.0±0.4	1.5±0.3	[illegible]	1.1±0.1	=	0.6±0.0	<	0.9±0.0	=
49:3	0.2±0.1	=	0.2±0.0	>	0.1±0.0	>	+	=	+	<	0.2±0.1	>	0.1±0.0	=	0.1±0.1	>	0.1±0.0	>	+	=	+	<	0.2±0.1	>	0.1±0.1	0.2±0.1	0.2±0.0	[illegible]	0.2±0.2	=	0.1±0.0	<	0.2±0.1	=
49:3	0.4±0.2	=	0.3±0.0	>	0.1±0.0	>	+	=	+	<	0.4±0.2	>	0.3±0.1	=	0.4±0.1	>	0.1±0.0	>	+	=	+	<	0.4±0.1	>	0.2±0.1	0.2±0.1	0.3±0.1	[illegible]	0.3±0.1	=	0.2±0.0	=	0.3±0.1	=
49:4	0.2±0.1	=	0.1±0.1	=	0.1±0.0	>	+	=	+	<	0.3±0.1	>	0.2±0.0	>	0.1±0.0	>	0.1±0.0	>	+	=	+	<	0.2±0.1	>	0.1±0.0	0.1±0.1	0.2±0.1	[illegible]	0.2±0.0	=	0.1±0.0	=	0.2±0.1	=
55:0	0.3±0.2	=	0.2±0.0	>	0.1±0.1	=	+	=	+	<	0.2±0.1	>	0.2±0.1	=	0.2±0.0	>	0.1±0.0	=	+	=	+	<	0.3±0.2	>	0.2±0.1	0.2±0.1	0.2±0.0	[illegible]	0.2±0.0	=	0.1±0.0	=	0.1±0.1	=
55:9	0.1±0.1	=	0.1±0.0	=	0.1±0.0	=	0.1±0.0	>	+	<	0.1±0.1	=	0.1±0.0	=	0.1±0.0	=	0.1±0.0	>	0.1±0.0	=	0.1±0.0	=	0.1±0.1	=	0.1±0.1	+	+	[illegible]	0.5±0.4	=	0.1±0.0	=	0.2±0.1	=
59:1	0.2±0.1	=	0.1±0.0	>	0.1±0.0	>	+	=	+	<	0.2±0.1	>	0.2±0.1	=	0.1±0.0	>	0.1±0.0	=	+	>	+	<	0.2±0.1	>	0.1±0.0	0.1±0.1	0.1±0.0	[illegible]	0.2±0.1	=	+	=	0.1±0.0	=
14:0/10:0/18:1	0.4±0.2	=	0.3±0.1	>	0.1±0.0	>	+	=	+	<	0.7±0.4	>	0.3±0.0	<	0.4±0.1	>	0.1±0.0	>	+	=	+	<	0.5±0.3	>	0.3±0.1	0.3±0.2	0.3±0.0	[illegible]	0.5±0.0	=	0.2±0.0	<	0.4±0.0	=
12:0/14:0/14:0	+	=	+	>	-	<	+	=	+	<	0.1±0.0	>	+	=	+	=	+	=	+	=	+	<	0.2±0.1	>	+	+	+	[illegible]	0.1±0.0	=	+	=	0.1±0.1	=
14:0/12:0/16:0	0.2±0.1	=	0.2±0.0	>	0.1±0.1	>	+	=	+	<	0.3±0.2	>	0.2±0.1	=	0.3±0.1	>	0.1±0.0	>	+	<	+	<	0.3±0.1	>	0.2±0.2	0.2±0.1	0.2±0.0	[illegible]	0.3±0.1	=	0.1±0.0	=	0.3±0.0	=
12:0/12:0/16:0	0.2±0.1	=	0.2±0.0	>	0.1±0.0	>	+	=	+	<	0.3±0.1	>	0.2±0.1	=	0.3±0.1	>	0.1±0.0	>	+	=	+	<	0.4±0.1	>	0.2±0.1	0.3±0.2	0.1±0.0	[illegible]	0.4±0.0	=	0.1±0.0	=	0.3±0.3	=
12:0/16:1/16:0	0.3±0.1	=	0.3±0.1	>	0.1±0.0	>	+	=	+	<	0.2±0.1	>	0.3±0.1	=	0.4±0.0	>	0.1±0.0	>	+	>	+	<	0.3±0.1	>	0.2±0.1	0.2±0.1	0.4±0.0	[illegible]	0.1±0.0	=	0.4±0.0	=	0.1±0.0	=
14:0/15:0/14:0	0.2±0.1	=	0.2±0.0	>	0.1±0.0	>	+	>	+	<	0.2±0.1	>	0.1±0.0	=	0.2±0.0	>	0.1±0.0	>	+	=	+	<	0.2±0.0	>	0.1±0.1	0.1±0.1	0.2±0.0	[illegible]	0.2±0.0	=	0.1±0.0	<	0.2±0.1	=

14:0/16:1/14:0	0.6±0.4	=	0.4±0.0	>	0.1±0.0	>	+	<	+	<	0.3±0.1	>	0.4±0.1	=	0.5±0.1	>	0.1±0.0	>	+	=	+	<	0.4±0.2	>	0.3±0.1	0.3±0.2	0.5±0.0	0.4±0.2	=	0.1±0.0	=	0.2±0.0	=
14:0/18:3/14:0	0.6±0.1	>	0.3±0.0	=	0.3±0.0	>	0.2±0.1	<	0.3±0.0	=	0.3±0.1	=	0.5±0.1	>	0.4±0.0	>	0.2±0.1	>	0.1±0.0	<	0.1±0.0	<	0.4±0.1	>	0.3±0.1	0.2±0.0	0.6±0.0	0.4±0.0	=	0.5±0.0	=	0.4±0.0	=
14:0/16:0/14:0	0.5±0.2	=	0.4±0.0	>	0.1±0.0	>	+	=	0.1±0.0	<	0.5±0.1	>	0.4±0.1	=	0.5±0.2	>	0.1±0.0	>	+	=	+	<	0.4±0.1	>	0.4±0.2	0.4±0.1	0.4±0.1	0.5±0.2	=	0.2±0.0	=	0.3±0.0	=
15:0/14:0/16:0	0.4±0.3	=	0.4±0.0	>	0.1±0.0	>	+	=	+	<	0.3±0.2	>	0.3±0.1	=	0.5±0.1	>	0.1±0.0	>	+	=	+	<	0.3±0.1	>	0.3±0.3	0.3±0.1	0.3±0.0	0.4±0.0	=	0.1±0.0	=	0.2±0.0	=
16:0/14:0/16:0	0.8±0.5	=	0.8±0.0	>	0.2±0.0	>	0.1±0.0	=	0.1±0.0	<	0.8±0.2	>	0.7±0.2	=	0.9±0.3	>	0.2±0.0	>	0.1±0.0	=	0.1±0.0	<	0.9±0.2	>	0.7±0.5	0.8±0.3	0.7±0.0	0.8±0.1	=	0.3±0.0	=	0.7±0.2	=
14:0/18:1/16:0	2.0±0.2	>	1.2±0.1	>	0.9±0.0	<	1.3±0.1	<	1.9±0.1	<	3.0±0.7	>	2.0±0.4	=	1.7±0.2	>	1.3±0.2	<	1.7±0.2	>	1.3±0.2	<	3.0±0.8	>	3.5±0.4	4.6±0.5	2.3±0.2	2.6±0.4	=	3.3±0.2	>	2.7±1.1	=
16:2/14:0/18:3	1.1±0.2	>	0.7±0.2	=	0.8±0.1	=	0.7±0.2	=	0.9±0.1	>	0.7±0.1	=	1.0±0.1	>	0.8±0.1	>	0.5±0.0	=	0.4±0.1	=	0.4±0.1	<	0.6±0.1	>	0.9±0.1	0.5±0.1	1.4±0.2	0.5±0.0	=	1.2±0.0	=	0.6±0.2	=
16:0/14:0/16:1	1.0±0.7	=	0.7±0.1	>	0.2±0.0	>	0.1±0.0	<	0.1±0.0	<	0.5±0.3	>	0.7±0.2	=	1.0±0.2	>	0.2±0.1	>	+	=	+	<	0.7±0.4	>	0.5±0.3	0.5±0.3	0.9±0.1	0.7±0.2	=	0.3±0.0	=	0.5±0.2	=
14:1/16:0/16:1	0.8±0.5	=	0.5±0.1	>	0.1±0.1	>	+	<	0.1±0.0	<	0.4±0.1	>	0.5±0.1	=	0.7±0.1	>	0.1±0.0	>	+	=	+	<	0.4±0.2	>	0.4±0.1	0.3±0.2	0.6±0.1	0.3±0.1	=	0.3±0.0	=	0.4±0.1	=
16:1/14:0/17:1	0.7±0.5	=	0.5±0.2	>	0.2±0.0	>	0.1±0.0	>	0.1±0.0	<	0.3±0.2	>	0.5±0.1	<	0.7±0.1	>	0.2±0.1	>	0.1±0.0	>	+	<	0.5±0.3	>	0.3±0.2	0.3±0.2	0.5±0.1	0.4±0.1	=	0.2±0.0	=	0.5±0.0	=
16:1/16:0/16:1	0.4±0.2	=	0.2±0.1	=	0.3±0.1	=	0.2±0.2	=	0.1±0.0	<	0.3±0.1	=	0.4±0.2	=	0.5±0.1	=	0.4±0.2	=	0.2±0.0	>	0.1±0.0	<	0.6±0.2	>	0.5±0.1	0.5±0.2	0.4±0.1	0.2±0.0	=	1.0±0.2	>	0.3±0.2	=
14:0/18:4/16:1 + 16:4/14:0/18:1	+	=	+	<	0.1±0.0	>	+	=	+	<	0.1±0.0	>	0.1±0.0	=	0.1±0.0	=	0.1±0.0	>	+	=	+	=	0.2±0.1	=	0.1±0.0	+	0.1±0.0	0.1±0.1	=	0.1±0.0	=	0.1±0.1	=
16:2/14:0/18:3	0.1±0.0	=	0.1±0.1	=	0.1±0.0	>	+	=	+	<	0.2±0.1	>	0.1±0.0	=	0.1±0.0	>	0.1±0.0	>	+	=	+	=	0.1±0.2	=	0.1±0.0	+	0.1±0.0	0.3±0.2	=	+	=	0.1±0.0	=
14:0/22:0/18:0	0.7±0.4	=	0.3±0.0	>	0.1±0.0	>	+	=	+	<	0.2±0.1	>	0.6±0.2	>	0.3±0.1	>	0.1±0.0	>	+	=	+	<	0.2±0.0	>	0.5±0.4	0.2±0.1	0.7±0.3	0.2±0.1	=	0.1±0.0	=	0.2±0.1	=
18:1/14:0/18:1	0.9±0.0	>	0.5±0.1	>	0.4±0.0	<	0.8±0.2	<	1.1±0.0	>	0.8±0.2	=	0.6±0.1	=	0.6±0.1	>	0.4±0.1	<	0.6±0.0	>	0.4±0.1	=	0.6±0.3	=	1.1±0.1	1.3±0.1	0.8±0.0	0.6±0.0	=	0.8±0.0	>	0.7±0.3	=
16:1/14:0/18:2	0.1±0.0	>	0.1±0.0	=	+	=	+	>	-	<	0.1±0.0	>	0.1±0.0	=	0.1±0.0	>	+	=	+	=	+	<	0.1±0.1	>	0.1±0.1	+	0.1±0.0	0.1±0.1	=	0.1±0.0	=	0.1±0.0	=
16:3/14:0/18:2	+	=	+	=	+	>	+	<	+	<	0.1±0.1	>	0.1±0.0	=	0.1±0.0	=	+	>	+	=	+	<	0.1±0.0	>	0.1±0.0	+	0.1±0.0	0.2±0.0	=	0.1±0.0	=	0.1±0.1	=
18:1/14:0/18:2	0.8±0.2	=	0.6±0.1	<	0.7±0.1	<	2.0±0.6	=	2.7±0.2	>	1.0±0.1	<	0.8±0.2	=	0.7±0.1	=	0.8±0.2	<	1.4±0.1	=	1.3±0.2	>	0.6±0.3	<	0.9±0.1	0.8±0.1	0.8±0.1	0.7±0.3	=	0.8±0.0	=	0.7±0.1	=
18:2/14:0/18:2	0.8±0.4	=	0.8±0.1	<	1.1±0.1	<	2.1±0.5	=	2.4±0.1	>	0.9±0.0	<	1.2±0.3	=	0.9±0.2	=	1.2±0.2	<	1.6±0.1	<	2.0±0.2	>	0.7±0.2	<	0.9±0.1	0.6±0.1	1.0±0.2	0.7±0.1	=	1.2±0.0	=	0.7±0.2	=
14:0/18:2/18:3	0.5±0.3	=	0.6±0.1	<	1.1±0.2	=	1.2±0.2	=	1.3±0.0	>	0.7±0.0	<	0.9±0.2	=	0.8±0.2	=	1.0±0.1	=	1.0±0.0	<	1.3±0.1	>	0.6±0.2	<	0.4±0.1	0.3±0.1	0.7±0.0	0.6±0.3	=	0.7±0.1	=	0.7±0.3	=
18:3/14:0/18:3	0.3±0.1	<	0.5±0.1	<	0.8±0.2	>	0.3±0.1	=	0.4±0.0	>	0.1±0.0	<	0.6±0.1	>	0.5±0.1	=	0.5±0.1	>	0.2±0.0	<	0.3±0.0	=	0.2±0.1	=	0.3±0.1	0.1±0.0	0.5±0.1	0.2±0.0	>	0.6±0.1	=	0.2±0.2	=
18:2/16:2/18:3 + 14:0/20:4/18:3	0.1±0.1	<	0.2±0.1	<	0.4±0.1	=	0.4±0.0	>	0.3±0.0	=	0.1±0.1	<	0.2±0.1	=	0.2±0.1	<	0.4±0.0	=	0.3±0.0	<	0.5±0.1	>	0.2±0.2	=	0.1±0.0	+	0.1±0.0	0.2±0.1	=	0.1±0.1	=	0.1±0.1	=
14:0/18:1/18:4	0.2±0.1	=	0.2±0.0	<	0.4±0.0	=	0.3±0.1	=	0.3±0.1	>	0.1±0.0	<	0.5±0.3	=	0.3±0.1	=	0.4±0.0	=	0.4±0.0	=	0.3±0.0	=	0.3±0.1	<	0.3±0.1	0.2±0.1	0.3±0.1	0.2±0.1	=	0.7±0.1	=	0.4±0.0	=
14:0/18:4/18:2	0.2±0.1	=	0.2±0.0	<	0.3±0.1	=	0.3±0.0	=	0.3±0.0	=	0.2±0.0	=	0.4±0.1	=	0.4±0.1	=	0.4±0.1	=	0.4±0.0	>	0.4±0.0	=	0.3±0.1	=	0.2±0.1	0.1±0.1	0.3±0.1	0.3±0.1	=	0.3±0.0	=	0.3±0.1	=
14:0/18:3/18:4	0.3±0.1	=	0.3±0.0	<	0.5±0.1	>	0.2±0.1	=	0.2±0.0	<	0.3±0.1	>	0.5±0.1	=	0.5±0.1	=	0.4±0.0	>	0.3±0.0	<	0.3±0.0	=	0.3±0.2	=	0.4±0.1	0.2±0.0	0.4±0.0	0.3±0.1	=	0.7±0.0	=	0.5±0.2	=
16:0/14:0/20:4	0.2±0.0	=	0.1±0.1	=	0.1±0.0	>	0.1±0.0	<	0.1±0.0	<	0.3±0.1	>	0.1±0.1	=	0.1±0.0	=	0.1±0.0	=	0.1±0.0	=	0.1±0.0	<	0.3±0.1	>	0.2±0.0	0.2±0.0	0.2±0.0	0.4±0.1	=	0.2±0.0	=	0.4±0.0	=
18:2/16:0/18:4 + 18:2/14:0/20:4	0.5±0.3	<	1.0±0.1	=	1.1±0.1	>	1.0±0.1	=	1.0±0.1	<	1.4±0.2	>	0.6±0.1	=	0.8±0.1	<	1.1±0.1	<	1.3±0.1	>	1.0±0.1	=	1.2±0.4	=	0.6±0.0	0.5±0.3	0.7±0.1	1.4±0.6	=	0.7±0.1	=	1.4±0.4	=
20:4/14:0/20:4	0.2±0.1	<	0.6±0.2	<	1.2±0.2	>	0.3±0.0	<	0.3±0.0	<	0.9±0.2	>	0.2±0.1	<	0.5±0.0	<	0.8±0.2	>	0.3±0.1	=	0.3±0.0	<	0.6±0.3	>	0.2±0.1	0.4±0.2	0.4±0.1	1.1±0.5	=	0.3±0.0	=	0.9±0.1	=
16:0/14:0/20:5	0.1±0.0	=	0.1±0.0	<	0.1±0.0	=	0.1±0.0	=	0.1±0.0	<	0.1±0.0	>	0.1±0.1	>	+	=	0.1±0.0	=	0.1±0.0	=	0.1±0.0	<	0.2±0.0	>	0.2±0.0	0.2±0.0	0.1±0.0	0.2±0.1	=	0.3±0.0	=	0.2±0.1	=
16:1/14:0/20:5	0.1±0.1	=	0.1±0.0	<	0.4±0.1	>	0.2±0.1	=	0.1±0.0	=	0.1±0.0	=	0.2±0.1	=	0.3±0.1	=	0.3±0.1	>	0.2±0.1	=	0.2±0.0	=	0.2±0.1	=	0.2±0.0	0.1±0.1	0.2±0.0	0.1±0.1	=	0.7±0.1	>	0.1±0.1	=
14:0/18:1/20:5	0.4±0.2	=	0.5±0.0	<	0.8±0.1	=	1.1±0.3	=	0.8±0.0	>	0.6±0.2	<	0.7±0.3	=	0.8±0.3	=	1.1±0.1	=	1.2±0.2	=	1.1±0.1	>	0.7±0.1	<	0.6±0.0	0.6±0.1	0.5±0.0	0.4±0.2	=	1.3±0.1	=	0.7±0.2	=
14:0/18:2/20:5	0.9±0.5	<	2.2±0.3	<	3.1±0.3	>	2.3±0.1	=	2.2±0.1	>	1.2±0.1	<	1.3±0.3	=	1.9±0.5	<	2.5±0.1	>	1.7±0.1	<	2.2±0.1	>	1.4±0.4	=	1.2±0.3	0.9±0.3	1.2±0.0	1.2±0.3	=	1.9±0.1	=	1.5±0.0	=
14:0/18:3/20:5 + 18:4/14:0/20:4	0.6±0.3	<	1.4±0.2	<	2.1±0.3	>	0.9±0.1	=	1.0±0.0	=	0.9±0.2	=	1.0±0.1	=	1.1±0.2	=	1.3±0.3	>	0.7±0.1	<	1.0±0.0	=	0.9±0.2	=	0.7±0.1	0.6±0.2	0.9±0.0	0.9±0.0	=	1.2±0.1	=	1.1±0.2	=
18:4/14:0/20:5	0.5±0.2	=	0.3±0.0	<	0.5±0.1	>	0.3±0.0	<	0.4±0.0	=	0.4±0.2	=	0.8±0.2	>	0.5±0.1	=	0.5±0.1	=	0.5±0.1	=	0.5±0.0	=	0.5±0.1	=	0.4±0.1	0.3±0.1	0.7±0.0	0.3±0.0	=	1.1±0.0	=	0.7±0.1	=
16:1/14:1/16:1 + 14:1/16:1/16:1	0.1±0.0	>	0.1±0.0	=	0.1±0.0	>	+	=	+	<	0.2±0.1	>	0.1±0.0	=	0.1±0.1	>	0.1±0.0	>	+	>	+	<	0.2±0.0	>	0.1±0.1	0.1±0.0	0.1±0.0	0.1±0.0	=	0.1±0.0	=	0.2±0.1	=
14:0/18:2/16:1+ 16:1/14:0/18:2	0.2±0.1	=	0.2±0.0	<	0.2±0.0	=	0.2±0.0	=	0.2±0.0	=	0.2±0.1	=	0.3±0.1	=	0.2±0.0	=	0.2±0.0	=	0.2±0.0	=	0.2±0.0	=	0.2±0.1	=	0.1±0.0	0.1±0.0	0.3±0.0	0.3±0.1	=	0.2±0.0	=	0.1±0.0	=
16:0/15:0/16:0	0.5±0.3	=	0.6±0.0	>	0.1±0.0	>	0.1±0.0	=	+	<	0.5±0.3	>	0.5±0.2	=	0.6±0.2	>	0.1±0.0	>	+	=	+	<	0.4±0.1	>	0.4±0.4	0.4±0.2	0.4±0.0	0.6±0.2	=	0.2±0.0	=	0.3±0.1	=
16:0/15:0/16:1	0.9±0.7	=	0.6±0.1	>	0.2±0.0	>	0.1±0.0	=	0.1±0.0	<	0.5±0.1	>	0.6±0.2	=	0.8±0.1	>	0.2±0.0	>	+	=	+	<	0.5±0.2	>	0.5±0.3	0.6±0.4	0.8±0.1	0.5±0.2	=	0.3±0.0	=	0.5±0.3	=
16:1/15:0/24:0	0.3±0.1	=	0.2±0.1	>	0.1±0.0	>	+	=	+	<	0.3±0.2	>	0.2±0.1	=	0.2±0.0	>	0.1±0.0	>	+	=	+	<	0.2±0.1	>	0.2±0.1	0.2±0.1	0.2±0.1	0.3±0.1	=	0.1±0.0	=	0.1±0.0	=

16:1/15:0/18:2	0.1±0.1	=	+	=	+	>	+	<	+	<	0.1±0.0	>	+	<	0.1±0.0	>	+	>	+	=	+	=	0.1±0.1	=	0.1±0.0	+	0.1±0.0	0.2±0.1	=	+	=	0.1±0.1	=
14:0/16:1/16:0	0.9±0.4	=	0.5±0.1	>	0.4±0.0	>	0.3±0.0	<	0.4±0.0	<	0.8±0.1	>	0.9±0.3	=	0.7±0.1	>	0.4±0.1	>	0.3±0.0	=	0.3±0.0	<	0.8±0.3	>	0.8±0.2	0.9±0.1	1.0±0.1	0.8±0.1	=	1.4±0.0	=	0.5±0.0	=
14:0/18:2/16:0	1.9±0.2	>	1.3±0.1	=	1.3±0.1	<	2.4±0.7	<	3.7±0.3	>	2.1±0.1	=	1.8±0.2	=	1.7±0.1	>	1.3±0.2	=	1.7±0.4	=	1.8±0.2	=	1.7±0.4	=	2.0±0.3	1.9±0.2	2.0±0.1	2.0±0.7	=	2.0±0.0	=	1.9±0.0	=
14:0/16:0/18:4	0.1±0.1	=	0.1±0.0	=	0.1±0.0	>	+	<	0.1±0.0	<	0.2±0.0	>	0.2±0.1	>	0.1±0.0	=	0.1±0.0	=	0.1±0.0	=	0.1±0.0	<	0.3±0.1	>	0.2±0.1	0.2±0.1	0.2±0.0	0.2±0.0	=	0.6±0.1	>	0.2±0.1	=
16:0/16:0/16:0	1.3±0.9	=	1.3±0.1	>	0.3±0.0	>	0.1±0.0	=	0.1±0.0	<	1.0±0.4	>	0.8±0.3	=	1.2±0.3	>	0.3±0.0	>	0.1±0.0	=	0.1±0.0	<	0.9±0.3	>	1.0±0.8	1.0±0.5	0.9±0.2	1.0±0.3	=	0.3±0.0	=	0.6±0.1	=
16:0/16:0/18:0	0.5±0.3	=	0.6±0.1	>	0.1±0.0	>	+	=	+	<	0.4±0.2	>	0.3±0.1	=	0.4±0.1	>	0.1±0.0	>	+	=	+	<	0.4±0.1	>	0.3±0.2	0.4±0.3	0.3±0.1	0.4±0.1	=	0.1±0.0	=	0.3±0.0	=
16:0/18:1/16:0	3.5±0.6	>	2.0±0.1	>	1.1±0.0	<	1.6±0.1	<	2.1±0.1	<	3.3±0.3	>	4.0±0.7	>	2.1±0.5	>	1.3±0.2	<	2.3±0.2	>	1.4±0.3	<	4.7±1.5	>	7.1±1.5	10.5±1.1	4.0±0.3	3.6±0.5	=	3.9±0.5	=	5.3±3.0	=
16:0/18:2/16:0	2.7±0.6	>	2.0±0.1	>	1.4±0.2	<	2.9±0.8	<	3.9±0.4	>	2.7±0.4	=	2.3±0.4	=	2.2±0.4	>	1.4±0.1	<	2.4±0.6	=	1.9±0.5	=	2.4±0.8	=	4.0±0.2	4.0±0.8	2.6±0.1	2.6±0.7	=	2.8±0.4	=	2.5±0.3	=
16:0/16:0/18:3	1.4±0.1	>	1.2±0.1	>	1.0±0.0	=	1.1±0.3	=	1.2±0.1	>	0.9±0.2	=	1.0±0.2	=	0.9±0.2	>	0.6±0.0	=	0.7±0.1	=	0.5±0.1	<	0.9±0.1	>	1.5±0.2	0.9±0.1	1.7±0.3	0.7±0.0	=	1.4±0.1	>	0.9±0.0	=
16:0/24:0/16:0	0.2±0.2	=	0.2±0.0	>	0.1±0.0	=	+	=	+	=	0.3±0.2	=	0.2±0.1	=	0.2±0.1	>	+	>	+	=	+	<	0.2±0.1	>	0.2±0.1	0.2±0.1	0.2±0.1	0.1±0.0	=	0.1±0.0	=	0.2±0.1	=
16:0/16:1/16:0	1.3±1.0	=	0.8±0.1	>	0.2±0.1	>	0.1±0.0	<	0.1±0.0	<	0.6±0.2	>	1.0±0.4	=	1.1±0.3	>	0.3±0.0	>	0.1±0.0	>	+	<	0.7±0.3	>	0.6±0.4	0.6±0.3	1.0±0.0	0.6±0.3	=	0.3±0.0	=	0.4±0.1	=
16:0/16:1/16:1	0.9±0.7	=	0.6±0.1	>	0.1±0.0	>	-	<	0.1±0.0	<	0.4±0.2	>	0.7±0.3	=	0.9±0.1	>	0.2±0.0	>	-	#	-	<	0.5±0.2	>	0.5±0.3	0.5±0.3	0.8±0.1	0.5±0.2	=	0.4±0.1	=	0.2±0.1	=
16:0/18:1/16:4	+	=	+	=	0.1±0.1	=	0.1±0.0	=	+	=	0.1±0.1	=	0.1±0.0	=	+	<	0.1±0.0	>	0.1±0.0	<	0.1±0.0	<	0.1±0.0	>	+	+	+	0.1±0.0	=	0.1±0.0	=	+	=
18:0/16:0/18:0 + 16:0/18:0/18:0	0.2±0.1	=	0.3±0.0	>	0.1±0.0	>	+	=	+	<	0.3±0.2	>	0.2±0.0	=	0.2±0.1	>	0.1±0.0	>	+	=	+	<	0.2±0.1	>	0.2±0.1	0.2±0.1	0.2±0.0	0.2±0.0	=	0.1±0.0	<	0.2±0.0	=
16:0/18:1/18:0	1.7±0.0	>	0.9±0.1	>	0.5±0.1	=	0.4±0.0	<	0.7±0.0	<	1.8±0.2	>	1.5±0.1	>	0.9±0.3	>	0.6±0.0	=	0.7±0.1	=	0.5±0.2	<	1.8±0.5	>	2.3±0.2	5.1±0.6	1.5±0.1	1.6±0.1	=	1.5±0.2	=	2.1±0.7	=
17:1/16:0/18:1	0.4±0.3	=	0.3±0.1	>	0.1±0.0	=	0.1±0.0	<	0.1±0.0	<	0.4±0.2	>	0.3±0.1	=	0.5±0.1	>	0.1±0.0	>	+	=	+	<	0.5±0.2	>	0.3±0.2	0.4±0.3	0.4±0.2	0.6±0.3	=	0.2±0.0	=	0.4±0.2	=
18:1/16:0/18:1	2.9±0.3	>	2.2±0.1	>	1.2±0.2	=	1.6±0.5	=	2.1±0.1	=	2.2±0.4	=	2.1±0.3	=	1.6±0.3	>	1.2±0.0	=	1.3±0.1	>	0.9±0.3	<	2.3±0.6	>	6.2±0.9	5.3±0.3	2.5±0.4	1.9±0.4	=	3.0±0.3	=	2.3±1.6	=
18:1/16:0/18:2	2.4±0.2	=	2.3±0.5	>	1.5±0.3	<	3.0±1.0	=	3.6±0.2	>	2.3±0.8	=	2.0±0.5	=	1.6±0.3	=	1.5±0.1	<	2.3±0.2	=	1.9±0.5	=	1.8±0.7	=	4.2±0.5	2.8±0.3	2.0±0.1	2.1±0.3	=	2.4±0.1	=	1.7±0.5	=
18:1/16:0/20:4	0.6±0.3	=	0.3±0.1	=	0.3±0.1	<	0.7±0.1	<	1.0±0.0	=	0.8±0.4	=	0.6±0.1	>	0.3±0.0	<	0.5±0.0	<	0.8±0.0	>	0.7±0.1	>	0.4±0.2	<	0.6±0.1	0.6±0.1	0.6±0.1	0.7±0.1	=	0.7±0.0	=	0.7±0.1	=
16:1/16:0/18:2	0.2±0.1	=	0.1±0.0	=	0.1±0.0	=	+	>	-	<	0.1±0.0	=	0.1±0.1	=	0.2±0.0	>	0.1±0.0	=	0.1±0.0	>	+	<	0.2±0.0	>	0.1±0.0	0.1±0.1	0.2±0.0	0.2±0.1	=	0.1±0.0	=	0.1±0.0	=
17:1/16:0/18:2	0.1±0.0	=	0.1±0.0	=	+	=	+	=	+	=	0.1±0.1	=	0.1±0.1	=	0.1±0.1	=	+	=	+	=	+	<	0.1±0.0	>	0.1±0.1	0.1±0.1	0.1±0.1	0.2±0.0	=	0.1±0.0	=	0.1±0.0	=
16:1/16:0/18:3 + 16:3/16:0/18:1	0.3±0.0	=	0.2±0.0	=	0.3±0.1	=	0.2±0.1	=	0.2±0.0	=	0.4±0.2	=	0.2±0.0	=	0.2±0.0	=	0.2±0.0	<	0.3±0.0	>	0.2±0.0	<	0.3±0.0	<	0.2±0.1	0.2±0.0	0.4±0.0	0.6±0.3	=	0.2±0.1	=	0.4±0.0	>
18:3/16:0/18:3 + 18:2/16:1/18:3	0.7±0.3	<	1.1±0.0	<	1.7±0.2	>	0.9±0.1	=	0.9±0.1	>	0.5±0.2	<	0.9±0.1	=	0.8±0.2	<	1.1±0.2	>	0.6±0.0	<	0.8±0.0	>	0.5±0.1	<	0.7±0.1	0.3±0.1	1.3±0.2	0.5±0.1	=	1.0±0.2	=	0.5±0.0	=
16:4/16:0/18:4	0.1±0.1	=	0.2±0.0	>	0.1±0.0	>	0.1±0.0	=	0.1±0.0	<	0.3±0.1	>	0.2±0.2	=	0.2±0.1	>	0.1±0.0	>	0.1±0.0	<	0.1±0.0	<	0.2±0.1	>	0.1±0.0	0.1±0.0	0.2±0.0	0.4±0.4	=	0.3±0.0	=	0.2±0.0	=
18:1/16:0/18:4 + 16:0/16:1/20:4	0.4±0.2	=	0.4±0.0	=	0.4±0.0	=	0.4±0.2	=	0.4±0.0	<	1.0±0.1	>	0.6±0.2	=	0.6±0.2	<	0.9±0.1	<	1.4±0.2	>	0.7±0.1	=	0.7±0.1	<	0.7±0.1	0.7±0.1	0.6±0.0	0.9±0.0	=	0.8±0.1	=	0.8±0.0	=
16:0/16:0/20:4	0.1±0.0	=	0.1±0.0	=	0.1±0.0	=	+	<	0.1±0.0	<	0.1±0.0	>	0.1±0.0	>	0.1±0.0	=	+	=	+	=	+	<	0.2±0.1	>	0.1±0.0	0.2±0.0	0.1±0.0	0.2±0.1	=	0.1±0.0	=	0.3±0.1	=
18:0/16:0/20:5	0.4±0.2	=	0.6±0.0	>	0.4±0.1	=	0.4±0.1	=	0.4±0.0	<	1.5±0.4	>	0.3±0.1	=	0.4±0.1	=	0.4±0.1	=	0.5±0.1	=	0.4±0.0	<	0.7±0.1	=	0.3±0.0	0.6±0.1	0.4±0.0	1.3±0.2	=	0.3±0.0	=	0.9±0.2	=
16:0/18:2/20:4	0.4±0.1	<	1.3±0.2	=	0.9±0.3	=	0.7±0.1	<	0.9±0.1	<	1.5±0.3	>	0.3±0.0	<	0.6±0.2	=	0.6±0.2	=	0.5±0.1	=	0.7±0.1	=	1.0±0.4	>	0.2±0.0	0.3±0.2	0.4±0.1	1.6±0.4	=	0.3±0.0	<	1.3±0.6	=
18:2/16:0/20:4	0.6±0.3	=	0.4±0.1	=	0.4±0.1	<	0.5±0.0	=	0.6±0.0	>	0.4±0.1	<	0.5±0.1	>	0.3±0.0	<	0.6±0.1	<	0.7±0.1	>	0.5±0.1	=	0.4±0.1	<	0.5±0.1	0.4±0.1	0.7±0.2	0.3±0.0	=	0.6±0.0	=	0.5±0.1	=
18:3/16:0/20:4	0.2±0.1	=	0.3±0.1	=	0.3±0.0	=	0.3±0.1	=	0.2±0.0	=	0.2±0.1	=	0.3±0.1	=	0.2±0.1	=	0.2±0.1	=	0.3±0.1	=	0.2±0.0	>	0.2±0.0	<	0.2±0.1	0.2±0.1	0.4±0.0	0.3±0.0	=	0.3±0.0	=	0.4±0.0	>
20:4/16:0/20:4	0.2±0.1	=	0.3±0.3	=	0.1±0.0	=	0.1±0.0	<	0.2±0.0	<	0.9±0.4	>	0.2±0.0	=	0.1±0.1	=	0.1±0.0	<	0.2±0.1	=	0.2±0.0	<	0.8±0.2	>	0.1±0.0	0.4±0.1	0.2±0.0	1.5±0.9	=	0.2±0.0	=	1.2±0.1	=
16:0/16:0/20:5	0.1±0.0	=	0.1±0.0	=	0.1±0.0	=	0.1±0.0	=	0.1±0.0	<	0.1±0.1	>	0.1±0.0	=	0.1±0.0	=	0.1±0.0	=	0.1±0.0	=	0.1±0.0	<	0.2±0.0	>	0.2±0.1	0.3±0.0	0.1±0.0	0.2±0.1	=	0.3±0.0	>	0.2±0.0	=
16:0/18:1/20:5	0.8±0.4	<	1.4±0.1	<	1.9±0.2	=	2.5±0.9	>	1.4±0.0	=	1.5±0.4	=	1.0±0.4	=	1.4±0.4	<	2.7±0.6	<	3.4±0.1	>	2.2±0.2	>	1.3±0.3	<	1.6±0.2	1.9±0.1	0.9±0.0	0.9±0.1	=	1.6±0.0	=	1.4±0.7	=
16:0/18:2/20:5	1.2±0.6	<	3.5±0.3	<	5.5±0.5	=	5.1±0.1	>	3.8±0.2	>	1.9±0.5	<	1.3±0.3	<	2.3±0.3	<	3.8±0.4	=	3.9±0.4	=	3.9±0.3	>	1.6±0.4	<	1.5±0.2	1.3±0.3	1.5±0.1	1.6±0.2	=	1.9±0.1	=	2.0±0.3	=
18:3/16:0/20:5	0.9±0.5	<	1.6±0.2	<	3.0±0.4	>	1.8±0.2	>	1.5±0.1	>	0.7±0.2	<	1.0±0.1	=	1.0±0.2	<	1.4±0.2	=	1.3±0.2	=	1.4±0.1	>	0.8±0.2	<	0.8±0.2	0.6±0.2	1.3±0.2	0.9±0.2	=	1.5±0.0	>	1.1±0.2	=
18:4/16:0/20:5	1.0±0.7	=	0.7±0.1	<	1.1±0.1	>	0.9±0.2	<	1.2±0.1	=	1.2±0.2	>	1.2±0.1	>	0.7±0.1	<	1.1±0.2	=	1.1±0.0	>	1.0±0.0	<	1.1±0.1	=	1.2±0.4	1.0±0.2	1.6±0.0	1.1±0.1	=	2.1±0.2	>	1.3±0.0	>
20:4/16:0/20:5	0.5±0.3	=	0.3±0.0	=	0.3±0.1	=	0.2±0.1	<	0.4±0.1	=	0.4±0.1	>	0.7±0.0	>	0.2±0.0	<	0.3±0.0	<	0.5±0.0	=	0.5±0.0	<	0.6±0.1	>	0.5±0.2	0.7±0.1	0.4±0.1	0.6±0.0	=	0.8±0.0	>	0.8±0.1	=
20:5/16:0/20:5	0.1±0.0	<	0.2±0.0	<	0.3±0.0	>	0.1±0.1	=	0.1±0.0	=	0.1±0.1	=	0.1±0.1	=	0.2±0.1	=	0.3±0.0	>	0.2±0.0	<	0.2±0.0	=	0.3±0.2	=	0.3±0.1	0.3±0.1	0.1±0.1	0.4±0.1	=	0.4±0.1	>	0.3±0.0	=
16:0/26:0/16:1 + 16:0/24:0/18:1	0.3±0.2	=	0.1±0.0	>	0.1±0.0	=	+	=	+	=	0.2±0.2	=	0.2±0.1	=	0.2±0.0	>	0.1±0.0	>	+	=	+	<	0.3±0.2	>	0.1±0.1	0.1±0.1	0.2±0.0	0.2±0.1	=	0.1±0.0	=	0.2±0.1	=
14:0/16:1/16:1	0.7±0.1	>	0.3±0.1	=	0.4±0.0	=	0.5±0.2	<	1.0±0.1	>	0.7±0.0	>	0.7±0.1	=	0.7±0.0	>	0.4±0.0	=	0.3±0.0	<	0.4±0.0	<	0.6±0.1	>	0.5±0.1	0.4±0.1	0.7±0.2	0.7±0.2	=	0.6±0.0	=	0.7±0.1	=

16:1/16:1/16:1	0.1±0.0	=	0.1±0.0	=	0.1±0.1	=	0.1±0.1	=	+	<	0.1±0.0	=	0.1±0.1	=	0.2±0.0	=	0.1±0.1	=	0.1±0.0	>	+	<	0.2±0.1	=	0.1±0.1	0.1±0.1	0.1±0.0	0.1±0.0	=	0.3±0.0	>	0.1±0.0	=
16:1/18:2/16:1	+	=	+	=	+	=	+	>	+	=	+	=	+	=	+	=	+	=	+	=	+	<	0.1±0.0	>	+	+	+	0.1±0.1	=	+	=	0.1±0.0	=
18:2/16:0/20:0	0.5±0.0	=	0.2±0.1	=	0.2±0.1	<	0.4±0.1	<	0.6±0.1	=	0.5±0.1	>	0.4±0.1	=	0.3±0.0	>	0.2±0.1	<	0.3±0.0	=	0.4±0.1	=	0.4±0.1	=	0.5±0.0	-	0.4±0.0	0.9±0.3	=	0.2±0.0	=	0.4±0.2	=
16:1/17:0/18:1	0.4±0.2	=	0.3±0.1	>	+	=	+	<	0.1±0.0	<	0.3±0.1	>	0.2±0.1	=	0.3±0.1	>	0.1±0.0	>	+	=	+	<	0.2±0.1	=	0.3±0.1	0.2±0.1	0.3±0.2	0.2±0.0	=	0.2±0.1	=	0.2±0.1	=
18:1/16:1/18:2	0.1±0.0	=	0.1±0.0	=	0.1±0.0	<	0.2±0.0	=	0.2±0.0	=	0.2±0.1	=	0.1±0.0	=	0.1±0.0	>	0.1±0.0	<	0.1±0.0	=	0.1±0.0	=	0.1±0.0	<	0.2±0.0	0.1±0.0	0.1±0.1	0.1±0.1	=	0.1±0.0	=	0.1±0.0	=
18:2/16:1/18:2	0.1±0.1	=	0.1±0.0	=	0.1±0.1	<	0.3±0.1	>	0.2±0.0	>	0.1±0.0	<	0.1±0.0	<	0.1±0.0	=	0.1±0.0	<	0.2±0.0	=	0.2±0.0	>	0.1±0.0	<	+	0.1±0.0	0.1±0.0	0.1±0.0	=	0.1±0.0	=	0.1±0.1	=
16:1/16:1/20:5	0.1±0.1	>	-	<	0.1±0.0	=	0.1±0.1	=	0.1±0.0	=	0.1±0.0	=	0.2±0.1	>	-	<	0.2±0.1	>	0.1±0.0	=	0.1±0.0	<	0.2±0.1	>	0.1±0.0	0.1±0.0	0.1±0.1	0.2±0.1	=	0.3±0.0	=	0.1±0.0	=
16:2/16:1/20:5	0.1±0.0	=	0.1±0.0	=	0.1±0.0	=	0.1±0.1	>	0.1±0.0	=	0.1±0.1	=	0.1±0.0	<	0.1±0.0	=	0.2±0.0	=	0.1±0.1	<	0.2±0.0	>	0.1±0.1	=	0.1±0.0	+	0.1±0.0	0.2±0.1	=	0.1±0.0	=	0.1±0.1	=
18:2/16:1/20:5 + 18:3/18:1/18:4	0.2±0.1	<	0.4±0.1	<	0.5±0.0	<	0.8±0.2	>	0.4±0.0	=	0.3±0.1	<	0.5±0.1	=	0.5±0.1	<	0.7±0.1	=	0.9±0.2	>	0.7±0.1	>	0.2±0.1	<	0.5±0.1	0.2±0.1	0.3±0.0	0.4±0.1	=	0.5±0.1	=	0.2±0.1	=
18:3/16:1/20:5	0.1±0.0	<	0.4±0.0	<	0.5±0.1	=	0.6±0.1	>	0.3±0.1	>	0.1±0.1	<	0.1±0.1	<	0.3±0.0	<	0.4±0.0	<	0.4±0.0	=	0.5±0.1	>	0.1±0.0	<	0.1±0.0	0.1±0.0	0.1±0.0	0.2±0.1	=	0.2±0.0	=	0.1±0.0	=
18:4/16:1/20:5	0.1±0.1	<	0.2±0.0	<	0.3±0.1	=	0.4±0.1	=	0.2±0.0	>	0.1±0.0	<	0.2±0.0	<	0.2±0.0	=	0.3±0.1	=	0.3±0.0	<	0.4±0.1	>	0.1±0.0	<	0.1±0.0	+	0.1±0.0	0.1±0.1	=	0.3±0.0	>	0.2±0.0	>
16:1/16:2/18:2	+	=	+	<	+	=	+	=	+	=	+	=	+	=	+	=	+	=	+	=	+	<	0.1±0.0	>	+	+	+	0.1±0.1	=	+	=	+	<
18:2/16:2/20:5	0.1±0.0	=	0.2±0.1	=	0.2±0.0	>	0.1±0.0	=	0.1±0.0	=	0.1±0.1	=	0.1±0.0	=	0.1±0.1	=	0.2±0.0	=	0.1±0.0	=	0.1±0.0	=	0.2±0.1	=	0.1±0.0	+	0.1±0.0	0.1±0.0	=	0.1±0.0	=	0.1±0.0	=
14:0/18:2/16:3	0.1±0.0	=	0.1±0.1	=	0.1±0.0	=	0.1±0.0	=	0.1±0.0	=	0.2±0.1	=	0.2±0.1	=	0.1±0.0	=	0.1±0.0	>	0.1±0.0	=	0.1±0.0	<	0.2±0.1	>	0.2±0.0	0.1±0.0	0.1±0.0	0.3±0.0	=	0.4±0.0	>	0.1±0.1	=
16:3/18:2/20:5	0.1±0.0	=	0.1±0.0	=	0.1±0.1	=	0.1±0.0	>	+	<	0.1±0.0	>	0.1±0.0	=	0.1±0.0	=	0.1±0.0	>	0.1±0.0	=	0.1±0.0	<	0.1±0.1	>	+	+	0.1±0.0	0.1±0.0	=	+	<	0.1±0.0	=
16:1/24:0/17:0	0.2±0.2	=	0.2±0.0	>	0.1±0.0	>	+	=	+	=	0.2±0.2	=	0.2±0.1	=	0.2±0.0	>	0.1±0.0	>	+	=	+	<	0.2±0.1	>	0.2±0.1	0.2±0.1	0.2±0.0	0.2±0.1	=	0.1±0.0	=	0.3±0.1	=
18:1/17:0/18:2	0.3±0.2	=	0.2±0.0	>	0.1±0.0	>	+	<	0.1±0.0	<	0.4±0.1	>	0.3±0.1	=	0.2±0.1	>	0.1±0.0	>	+	=	+	<	0.3±0.1	>	0.2±0.0	0.2±0.0	0.2±0.0	0.3±0.1	=	0.1±0.0	=	0.1±0.0	=
18:2/17:0/18:2	0.1±0.1	=	0.1±0.0	>	0.1±0.0	>	+	=	+	<	0.3±0.1	>	0.2±0.1	=	0.1±0.0	>	0.1±0.0	>	+	=	+	<	0.3±0.2	>	0.1±0.0	0.1±0.1	0.1±0.0	0.3±0.2	=	0.1±0.0	=	0.2±0.1	=
16:1/17:1/18:1	0.5±0.3	=	0.4±0.1	>	0.1±0.0	>	0.1±0.0	<	0.1±0.0	<	0.4±0.1	>	0.3±0.1	=	0.4±0.1	>	0.1±0.0	>	0.1±0.0	=	0.1±0.0	<	0.4±0.2	>	0.3±0.1	0.2±0.1	0.4±0.1	0.2±0.0	=	0.1±0.0	=	0.1±0.1	=
16:0/18:0/18:2	1.2±0.2	>	0.6±0.2	>	0.3±0.1	<	0.6±0.2	<	1.1±0.2	=	1.1±0.2	>	0.9±0.2	=	0.7±0.2	>	0.4±0.0	=	0.6±0.2	=	0.7±0.2	=	0.9±0.2	=	1.1±0.1	1.6±0.4	0.8±0.0	1.0±0.2	=	0.9±0.2	=	1.2±0.2	=
16:0/18:0/18:3	0.7±0.2	>	0.3±0.0	>	0.2±0.0	=	0.3±0.1	<	0.4±0.1	>	0.3±0.1	=	0.5±0.0	>	0.3±0.1	>	0.2±0.0	=	0.2±0.0	=	0.2±0.1	<	0.3±0.1	>	0.5±0.1	0.4±0.1	0.7±0.0	0.3±0.0	=	0.6±0.0	>	0.4±0.1	=
16:0/24:0/18:0	0.3±0.1	>	0.1±0.0	=	0.1±0.1	>	+	=	+	<	0.3±0.1	>	0.2±0.1	=	0.2±0.0	>	0.1±0.0	>	+	>	+	=	0.3±0.3	=	0.2±0.0	0.2±0.1	0.1±0.0	0.3±0.2	=	0.1±0.0	=	0.1±0.0	=
18:0/16:1/24:0	0.1±0.0	=	0.1±0.0	=	0.1±0.0	>	+	=	+	<	0.1±0.0	>	+	=	+	>	+	>	+	=	+	<	0.1±0.0	>	+	0.1±0.1	0.1±0.1	1.0±1.3	=	+	=	0.1±0.0	=
18:0/18:1/18:0 + 16:0/20:0/18:1	0.8±0.1	>	0.5±0.1	>	0.2±0.1	=	0.3±0.0	<	0.4±0.0	<	1.0±0.2	>	0.7±0.0	>	0.5±0.1	>	0.3±0.0	=	0.4±0.0	=	0.3±0.1	<	0.7±0.0	>	0.6±0.2	1.0±0.1	0.6±0.0	0.8±0.1	=	0.4±0.1	=	0.6±0.1	=
18:1/18:0/18:2	0.7±0.2	=	0.7±0.0	>	0.2±0.1	=	0.3±0.0	<	0.5±0.1	=	0.5±0.1	>	0.5±0.1	=	0.6±0.2	>	0.3±0.0	<	0.4±0.0	=	0.4±0.1	=	0.4±0.1	=	0.5±0.2	0.7±0.2	0.5±0.1	0.6±0.1	=	0.5±0.1	=	0.4±0.0	=
18:0/18:1/20:4	0.1±0.0	=	0.1±0.0	=	+	=	+	<	0.1±0.0	<	0.1±0.0	>	0.1±0.0	>	+	=	+	<	0.1±0.0	=	0.1±0.0	=	0.1±0.0	=	+	0.1±0.0	0.1±0.0	0.1±0.0	=	+	=	0.1±0.0	>
18:0/18:1/20:5	0.2±0.1	=	0.2±0.0	=	0.1±0.0	<	0.3±0.1	=	0.3±0.0	=	0.2±0.1	=	0.2±0.1	>	0.1±0.0	<	0.3±0.1	<	0.4±0.1	=	0.3±0.1	=	0.2±0.1	<	0.2±0.0	0.3±0.0	0.2±0.0	0.1±0.0	=	0.2±0.0	=	0.3±0.1	=
18:0/18:2/20:5	0.3±0.2	=	0.3±0.1	=	0.2±0.0	<	0.3±0.0	<	0.4±0.0	>	0.3±0.1	=	0.3±0.1	>	0.2±0.0	<	0.3±0.0	=	0.4±0.1	=	0.4±0.1	=	0.3±0.1	=	0.2±0.0	0.2±0.0	0.2±0.0	0.4±0.2	=	0.3±0.0	=	0.4±0.2	=
18:3/18:0/20:5	0.2±0.2	=	0.1±0.1	=	0.2±0.0	>	0.2±0.0	<	0.2±0.0	>	0.2±0.0	=	0.2±0.0	>	0.1±0.0	<	0.2±0.0	>	0.2±0.0	=	0.2±0.0	<	0.3±0.0	>	0.2±0.0	0.1±0.0	0.3±0.0	0.1±0.0	=	0.3±0.0	=	0.3±0.1	=
20:4/18:1/20:4 + 20:4/18:0/20:5	0.1±0.0	=	0.1±0.1	=	0.1±0.0	>	+	<	0.1±0.0	<	0.3±0.1	>	0.1±0.0	>	+	=	+	=	0.1±0.0	=	0.1±0.0	<	0.2±0.1	>	+	0.1±0.0	+	0.3±0.1	=	+	=	0.2±0.1	=
18:1/16:1/18:1	0.1±0.0	>	0.1±0.0	=	0.1±0.0	=	0.1±0.0	=	0.1±0.0	<	0.2±0.0	>	0.1±0.1	=	0.1±0.0	=	0.1±0.0	=	0.1±0.0	>	0.1±0.0	=	0.1±0.1	=	0.2±0.0	0.2±0.1	0.1±0.0	0.1±0.0	=	0.1±0.0	=	0.1±0.0	=
16:0/18:2/18:3	1.2±0.6	=	1.4±0.1	<	2.3±0.3	=	2.6±0.4	=	2.7±0.1	>	1.5±0.5	<	1.3±0.2	=	1.2±0.2	<	1.9±0.2	=	2.1±0.1	=	2.1±0.2	>	0.9±0.2	<	1.3±0.2	0.7±0.1	1.4±0.2	1.4±0.3	=	1.5±0.1	=	1.2±0.2	=
16:1/22:0/18:1	0.2±0.1	=	0.1±0.0	>	+	=	+	<	0.1±0.0	<	0.2±0.1	>	0.1±0.0	=	0.1±0.0	>	+	=	+	=	+	<	0.2±0.1	>	+	0.1±0.0	0.1±0.0	0.2±0.0	=	+	<	0.1±0.1	=
16:4/18:1/18:4	0.1±0.0	=	0.1±0.0	=	0.1±0.0	>	+	=	+	<	0.1±0.0	=	0.1±0.0	=	0.1±0.0	=	0.1±0.0	>	+	<	0.1±0.0	=	0.1±0.1	=	+	+	0.1±0.0	0.1±0.0	=	0.1±0.1	=	0.2±0.0	=
18:1/18:0/18:1	1.3±0.4	>	0.6±0.1	>	0.3±0.1	=	0.2±0.0	<	0.3±0.0	<	0.6±0.1	>	0.7±0.1	=	0.5±0.2	>	0.3±0.1	>	0.2±0.0	=	0.2±0.1	<	0.6±0.1	>	1.0±0.2	1.5±0.2	0.8±0.1	0.4±0.1	=	0.5±0.0	=	0.5±0.3	=
18:0/20:0/18:1 + 18:1/14:0/24:0	0.4±0.1	>	0.2±0.1	>	0.1±0.0	>	0.1±0.0	<	0.1±0.0	<	0.2±0.1	>	0.3±0.1	=	0.2±0.0	>	0.1±0.0	>	0.1±0.0	>	+	<	0.3±0.1	>	0.2±0.1	0.3±0.1	0.3±0.0	0.3±0.3	=	0.1±0.0	=	0.2±0.1	=
18:1/18:1/18:1	4.4±1.2	=	2.5±1.3	>	0.9±0.2	=	0.6±0.2	=	2.0±1.9	=	0.8±0.0	=	2.8±1.0	>	0.7±0.2	=	0.7±0.2	>	0.4±0.1	>	0.3±0.1	<	0.7±0.2	>	2.8±0.8	1.1±0.3	2.7±0.9	0.6±0.2	=	1.3±0.0	=	0.6±0.2	=
18:1/18:2/18:1	2.7±0.7	=	1.9±0.8	>	0.8±0.2	=	0.7±0.2	<	0.9±0.0	>	0.7±0.1	=	1.6±0.6	>	0.8±0.2	=	0.8±0.3	=	0.7±0.1	=	0.6±0.2	=	0.7±0.2	=	1.8±0.4	0.8±0.2	1.6±0.4	0.7±0.3	=	0.9±0.0	=	0.5±0.1	=
18:1/20:0/18:1	0.3±0.0	>	0.2±0.1	>	0.1±0.0	=	0.1±0.0	<	0.1±0.0	=	0.1±0.0	=	0.2±0.0	>	0.2±0.0	>	0.1±0.0	>	0.1±0.0	=	0.1±0.0	=	0.1±0.1	=	0.2±0.1	0.2±0.1	0.2±0.0	0.5±0.4	=	0.1±0.0	<	0.2±0.1	=
18:1/20:3/18:1	0.2±0.1	>	0.1±0.0	>	0.1±0.0	<	0.1±0.0	<	0.2±0.0	>	0.1±0.0	=	0.1±0.0	=	0.1±0.0	<	0.1±0.0	<	0.1±0.0	=	0.1±0.0	=	0.3±0.2	=	0.2±0.0	0.2±0.0	0.2±0.0	0.2±0.0	=	0.1±0.0	=	0.1±0.0	=

18:2/18:2/18:2 + 18:2/18:1/18:3	1.6±0.2	=	1.5±0.2	>	1.1±0.2	=	1.7±0.5	=	1.6±0.1	>	0.7±0.3	<	1.6±0.6	=	1.1±0.3	=	1.4±0.5	=	1.5±0.2	=	1.5±0.1	>	0.8±0.4	<	1.3±0.2	0.7±0.2	1.4±0.1	0.7±0.0	=	0.9±0.1	=	0.3±0.0	=
18:2/18:1/18:4	0.3±0.1	=	0.2±0.1	=	0.2±0.1	=	0.3±0.1	=	0.3±0.1	=	0.3±0.1	=	0.3±0.2	=	0.3±0.1	<	0.5±0.1	=	0.8±0.3	>	0.5±0.1	>	0.1±0.0	<	0.2±0.1	0.1±0.1	0.3±0.1	0.3±0.0	=	0.2±0.1	=	0.2±0.0	=
18:1/18:1/20:4	0.2±0.1	=	0.2±0.1	=	0.1±0.0	<	0.2±0.0	=	0.3±0.0	=	0.3±0.1	=	0.2±0.0	>	0.1±0.0	=	0.1±0.0	<	0.2±0.0	>	0.2±0.0	=	0.2±0.0	<	0.1±0.0	0.2±0.0	0.2±0.0	0.2±0.0	=	0.1±0.0	=	0.2±0.0	=
20:4/18:1/20:5	0.1±0.1	<	0.4±0.1	=	0.3±0.1	=	0.3±0.1	=	0.3±0.0	<	0.8±0.4	>	0.2±0.0	=	0.2±0.0	=	0.2±0.0	<	0.5±0.1	=	0.5±0.1	=	1.0±0.5	=	0.1±0.0	0.4±0.3	0.1±0.0	1.1±0.4	=	0.2±0.0	=	1.0±0.4	=
18:1/18:1/20:5	0.4±0.2	<	0.7±0.1	=	0.6±0.2	<	1.3±0.1	>	1.0±0.0	>	0.6±0.3	<	0.3±0.1	=	0.4±0.1	<	0.8±0.3	<	1.2±0.1	>	0.9±0.1	>	0.3±0.0	<	0.4±0.0	0.5±0.1	0.4±0.0	0.5±0.1	=	0.3±0.0	=	0.4±0.1	=
18:2/18:1/20:5	0.6±0.3	<	1.0±0.1	=	0.9±0.1	<	2.4±0.2	>	1.7±0.0	>	1.1±0.1	<	0.6±0.2	<	1.0±0.2	<	1.6±0.4	<	2.5±0.2	=	2.2±0.1	>	0.7±0.2	<	0.4±0.0	0.5±0.2	0.6±0.1	0.9±0.3	=	0.4±0.1	=	0.8±0.0	=
18:3/18:1/20:5	0.3±0.2	<	0.6±0.1	=	0.7±0.2	<	1.4±0.5	>	0.3±0.1	=	0.4±0.2	<	0.5±0.2	=	0.5±0.2	<	1.2±0.3	=	1.3±0.5	>	0.5±0.2	>	0.1±0.1	<	0.3±0.0	0.3±0.0	0.5±0.1	0.4±0.1	=	0.6±0.0	=	0.3±0.1	=
18:4/18:1/20:5	0.6±0.4	=	0.4±0.1	=	0.5±0.0	<	0.7±0.1	=	0.7±0.1	=	0.5±0.2	=	0.5±0.1	=	0.5±0.1	<	0.7±0.1	<	1.0±0.1	>	0.7±0.1	>	0.4±0.1	<	0.4±0.1	0.4±0.1	0.6±0.1	0.7±0.2	=	0.5±0.0	=	0.6±0.3	=
20:0/18:1/20:5	0.1±0.0	=	0.1±0.0	=	0.1±0.0	<	0.1±0.0	<	0.1±0.0	=	0.2±0.0	>	0.1±0.0	=	0.1±0.0	=	0.1±0.0	<	0.1±0.0	=	0.1±0.0	=	0.1±0.1	=	+	0.1±0.0	0.1±0.0	0.3±0.0	>	+	=	0.2±0.2	=
20:5/18:1/20:5	0.1±0.1	<	0.2±0.0	<	0.3±0.1	=	0.6±0.3	>	0.2±0.0	=	0.3±0.1	=	0.2±0.1	=	0.3±0.1	<	0.5±0.1	=	0.6±0.1	=	0.6±0.2	>	0.3±0.0	<	0.2±0.0	0.2±0.0	0.1±0.0	0.1±0.0	=	0.2±0.0	=	0.3±0.1	=
18:2/16:0/18:2	2.5±0.5	=	2.7±0.4	=	2.5±0.3	<	4.0±0.8	=	4.4±0.2	>	3.1±1.0	=	2.4±0.3	=	2.2±0.2	<	2.8±0.2	<	3.8±0.2	=	3.2±0.5	>	1.9±0.6	<	3.4±0.4	2.4±0.2	2.7±0.1	2.7±0.4	=	2.7±0.1	=	2.0±0.4	=
16:3/18:2/18:3	0.1±0.0	<	0.1±0.0	<	0.3±0.0	>	0.2±0.0	>	0.1±0.0	=	0.1±0.1	=	0.1±0.0	<	0.2±0.0	=	0.2±0.1	>	0.2±0.0	=	0.2±0.0	>	0.1±0.1	<	+	+	0.1±0.1	0.1±0.0	=	0.1±0.0	=	0.1±0.1	=
16:4/18:2/18:3	+	<	0.1±0.0	<	0.3±0.1	>	0.1±0.0	>	0.1±0.0	=	0.1±0.0	=	0.1±0.0	=	0.1±0.0	=	0.2±0.1	>	0.1±0.0	<	0.2±0.0	>	0.1±0.1	=	+	+	0.1±0.0	0.1±0.0	<	0.1±0.0	=	0.1±0.0	=
18:2/18:0/18:2	0.9±0.2	>	0.5±0.1	>	0.4±0.0	<	0.6±0.1	<	0.9±0.1	=	0.9±0.3	=	0.8±0.1	>	0.5±0.1	=	0.5±0.1	<	0.7±0.1	=	0.7±0.2	=	0.6±0.0	=	0.7±0.0	0.9±0.1	0.8±0.1	0.8±0.0	=	0.6±0.0	=	0.8±0.0	>
18:2/18:1/18:2	2.9±0.6	=	2.7±0.7	>	2.0±0.1	=	2.6±0.6	=	2.7±0.1	>	1.5±0.4	<	2.0±0.4	>	1.5±0.2	<	2.1±0.4	=	2.1±0.1	=	1.7±0.3	=	1.5±0.3	<	2.5±0.3	1.4±0.1	2.2±0.4	1.3±0.1	=	1.6±0.1	=	1.3±0.1	=
18:1/20:0/18:2 + 18:1/20:1/18:1	0.4±0.1	>	0.3±0.1	>	0.1±0.0	=	0.1±0.0	<	0.2±0.0	<	0.3±0.1	>	0.3±0.0	>	0.2±0.0	>	0.1±0.0	<	0.2±0.0	=	0.2±0.0	=	0.2±0.1	=	0.2±0.0	0.2±0.1	0.3±0.1	0.6±0.2	=	0.1±0.0	<	0.2±0.1	=
18:1/18:2/20:3	0.2±0.1	>	0.1±0.0	=	0.1±0.0	<	0.3±0.1	<	0.4±0.0	>	0.3±0.0	=	0.1±0.0	=	0.1±0.0	=	0.2±0.0	<	0.2±0.0	=	0.2±0.0	=	0.2±0.1	=	0.2±0.0	0.2±0.0	0.2±0.0	0.3±0.0	=	0.2±0.0	=	0.2±0.1	=
18:2/18:1/20:1 + 18:2/20:0/18:2	0.3±0.0	>	0.1±0.0	=	0.1±0.0	<	0.3±0.1	<	0.4±0.0	>	0.3±0.0	=	0.3±0.0	>	0.2±0.0	=	0.2±0.0	=	0.2±0.0	<	0.3±0.0	>	0.2±0.0	<	0.1±0.1	0.2±0.0	0.2±0.1	0.3±0.1	=	0.1±0.0	<	0.2±0.0	=
18:2/20:4/18:2	0.4±0.2	=	0.2±0.0	<	0.2±0.0	<	0.3±0.1	=	0.4±0.0	>	0.4±0.0	=	0.4±0.1	>	0.2±0.0	<	0.3±0.1	=	0.3±0.1	<	0.5±0.1	>	0.3±0.1	=	0.3±0.0	0.2±0.0	0.3±0.0	0.3±0.1	=	0.5±0.0	=	0.3±0.0	=
18:2/18:3/18:2	0.8±0.3	=	0.9±0.3	=	1.2±0.2	=	1.6±0.4	>	0.9±0.0	>	0.6±0.2	<	1.0±0.1	>	0.8±0.1	<	1.3±0.1	=	1.3±0.1	=	1.4±0.1	>	0.6±0.2	<	0.7±0.1	0.3±0.0	1.0±0.1	0.6±0.1	=	0.8±0.1	=	0.6±0.2	=
18:3/18:2/18:4	0.2±0.1	<	0.4±0.1	=	0.4±0.1	>	0.1±0.1	=	0.1±0.0	=	0.1±0.1	=	0.3±0.0	=	0.3±0.1	=	0.3±0.0	>	0.1±0.0	=	0.1±0.1	=	0.2±0.0	>	0.1±0.0	+	0.2±0.0	0.2±0.0	=	0.2±0.1	=	0.1±0.1	=
18:2/18:3/20:0	0.2±0.1	=	0.1±0.1	=	0.1±0.0	<	0.2±0.0	<	0.3±0.0	=	0.2±0.1	=	0.2±0.1	>	0.1±0.0	<	0.2±0.0	=	0.2±0.0	<	0.2±0.0	>	0.1±0.0	=	0.1±0.0	0.1±0.0	0.2±0.0	0.2±0.1	=	0.1±0.0	=	0.1±0.0	=
18:3/18:2/20:4	0.2±0.1	=	0.1±0.0	<	0.2±0.0	=	0.2±0.0	=	0.2±0.0	=	0.1±0.0	=	0.2±0.0	>	0.1±0.0	<	0.2±0.0	=	0.2±0.1	=	0.2±0.0	>	0.1±0.0	<	0.1±0.0	+	0.3±0.0	0.2±0.1	=	0.2±0.1	=	0.1±0.0	=
20:3/18:2/20:4 + 20:3/18:1/20:5	0.1±0.0	=	0.1±0.0	<	0.1±0.0	<	0.2±0.0	=	0.2±0.0	<	0.3±0.1	=	0.1±0.0	=	0.1±0.0	=	0.1±0.0	<	0.2±0.0	=	0.2±0.0	=	0.2±0.1	=	0.1±0.0	0.1±0.1	0.1±0.0	0.2±0.0	=	0.1±0.0	<	0.2±0.1	=
20:4/18:2/20:5	0.2±0.1	<	0.5±0.1	=	0.6±0.1	<	0.9±0.2	>	0.7±0.0	=	0.7±0.3	=	0.4±0.0	=	0.4±0.1	<	0.7±0.0	<	1.2±0.2	=	1.4±0.3	=	1.2±0.7	=	0.2±0.0	0.5±0.4	0.2±0.1	1.1±0.2	=	0.3±0.0	=	1.3±0.2	=
18:2/18:2/20:5	0.5±0.4	<	1.4±0.2	=	1.9±0.5	<	2.8±0.6	=	2.4±0.1	>	1.1±0.2	<	0.6±0.1	<	1.2±0.2	<	1.9±0.4	<	2.8±0.6	<	3.8±0.4	>	1.0±0.4	<	0.3±0.0	0.4±0.3	0.6±0.0	0.9±0.3	=	0.5±0.0	<	0.9±0.0	=
18:3/18:2/20:5	0.9±0.5	<	2.5±0.1	<	3.3±0.4	=	3.4±0.3	>	2.0±0.1	>	1.0±0.4	<	1.3±0.3	<	2.2±0.4	<	3.5±0.1	>	3.1±0.3	=	3.9±0.7	>	1.3±0.4	<	0.5±0.1	0.6±0.4	1.2±0.1	0.6±0.1	=	1.1±0.1	=	1.4±0.5	=
18:2/18:4/20:5	0.7±0.4	=	0.8±0.2	<	1.3±0.2	=	1.1±0.4	=	0.9±0.1	=	1.2±0.3	=	1.3±0.2	=	1.4±0.4	=	1.7±0.1	<	2.0±0.2	=	2.1±0.4	>	1.5±0.3	<	0.6±0.1	0.7±0.4	0.9±0.1	1.3±0.1	=	1.2±0.0	=	1.6±0.7	=
20:0/18:2/20:5	0.1±0.0	>	0.1±0.0	=	0.1±0.0	=	0.1±0.0	<	0.2±0.0	=	0.2±0.1	=	0.1±0.0	>	0.1±0.0	=	0.1±0.0	<	0.1±0.0	<	0.2±0.0	=	0.1±0.0	=	0.1±0.0	0.1±0.0	0.1±0.0	0.2±0.1	=	0.1±0.0	<	0.2±0.1	=
20:3/18:2/20:5	0.2±0.1	=	0.1±0.0	<	0.3±0.1	<	0.5±0.1	>	0.4±0.0	>	0.2±0.1	<	0.2±0.1	=	0.2±0.1	<	0.3±0.1	<	0.5±0.1	=	0.5±0.1	>	0.2±0.1	<	0.2±0.0	0.1±0.1	0.2±0.0	0.3±0.0	=	0.2±0.0	=	0.2±0.1	=
20:4/18:2/20:5	0.2±0.1	=	0.2±0.0	<	0.3±0.0	<	0.4±0.1	=	0.3±0.0	=	0.3±0.1	<	0.3±0.1	=	0.2±0.0	<	0.4±0.0	=	0.4±0.1	=	0.6±0.2	>	0.2±0.0	<	0.1±0.0	0.1±0.1	0.3±0.0	0.2±0.1	=	0.3±0.0	=	0.2±0.1	=
20:5/18:2/20:5	0.8±0.4	<	1.5±0.0	<	2.3±0.2	=	2.7±0.6	>	1.6±0.1	>	1.3±0.0	<	0.9±0.2	<	1.4±0.3	<	2.4±0.1	<	2.7±0.1	=	3.4±0.9	>	1.9±0.8	=	0.5±0.0	1.0±0.7	1.1±0.1	1.2±0.1	=	1.1±0.0	=	1.8±0.4	=
18:2/18:2/22:0 + 20:0/18:0/20:4	0.1±0.0	>	0.1±0.0	=	0.1±0.0	>	+	<	+	<	0.1±0.1	>	0.1±0.0	=	0.1±0.0	=	+	>	+	<	+	=	0.2±0.2	=	+	0.1±0.0	0.1±0.0	0.3±0.0	=	+	=	0.1±0.1	=
18:3/16:0/20:0	0.4±0.0	>	0.2±0.0	>	0.1±0.0	=	0.1±0.0	=	0.2±0.0	=	0.2±0.1	=	0.3±0.0	>	0.1±0.0	=	0.1±0.0	>	0.1±0.0	=	0.1±0.0	<	0.2±0.0	>	0.1±0.0	0.1±0.0	0.3±0.0	0.1±0.0	=	0.2±0.0	<	0.2±0.1	=
18:3/18:3/18:3	0.2±0.1	<	0.4±0.1	=	0.4±0.1	>	0.1±0.1	=	0.1±0.0	=	0.1±0.1	=	0.3±0.0	=	0.3±0.1	=	0.3±0.0	>	0.1±0.0	=	0.1±0.1	=	0.2±0.0	>	0.1±0.0	+	0.2±0.0	0.2±0.0	=	0.2±0.1	=	0.1±0.1	=
18:3/18:4/18:3	0.3±0.1	=	0.5±0.1	=	0.6±0.2	>	0.2±0.1	=	0.2±0.0	<	0.5±0.1	>	0.6±0.1	=	0.7±0.1	=	0.8±0.1	>	0.5±0.1	=	0.4±0.1	=	0.5±0.1	=	0.3±0.0	0.3±0.1	0.5±0.1	0.4±0.1	=	0.5±0.0	=	0.4±0.3	=
18:3/18:3/18:4	0.1±0.0	=	0.1±0.0	=	0.1±0.0	=	0.1±0.0	=	0.1±0.0	=	0.1±0.0	=	0.1±0.0	=	0.1±0.0	=	0.1±0.0	>	0.1±0.0	<	0.2±0.0	=	0.1±0.1	=	0.1±0.0	0.1±0.0	+	0.2±0.2	=	0.2±0.0	=	0.1±0.1	=
18:4/18:3/18:4 + 18:4/16:2/20:5	0.1±0.0	<	0.4±0.1	<	0.7±0.0	>	0.4±0.2	>	0.2±0.0	=	0.3±0.1	=	0.4±0.1	<	0.6±0.1	=	0.7±0.1	>	0.5±0.1	=	0.6±0.1	>	0.4±0.1	=	0.2±0.1	0.2±0.1	0.3±0.0	0.5±0.1	=	0.5±0.0	=	0.5±0.1	=
18:3/18:3/20:5	0.5±0.3	<	1.5±0.0	<	2.3±0.1	>	0.9±0.1	>	0.7±0.1	>	0.3±0.2	<	0.9±0.2	<	1.3±0.2	<	1.9±0.2	>	0.6±0.2	<	1.0±0.2	>	0.5±0.1	=	0.4±0.1	0.3±0.2	0.7±0.0	0.3±0.1	=	0.9±0.0	=	0.6±0.0	=

18:4/18:3/20:5	0.9±0.6	=	1.7±0.4	<	3.1±0.2	>	1.6±0.5	=	1.5±0.1	>	1.2±0.2	=	2.6±0.4	=	3.3±0.5	=	3.7±0.4	>	2.5±0.4	=	2.8±0.5	>	1.7±0.4	<	1.3±0.2	1.1±0.6	1.5±0.0	[illegible]	1.2±0.4	=	2.7±0.0	=	1.9±0.4	=
18:3/20:5/20:4	0.1±0.0	<	0.1±0.0	=	0.1±0.0	>	0.1±0.0	=	0.1±0.0	=	0.1±0.0	=	0.2±0.1	=	0.1±0.1	=	0.1±0.0	=	0.2±0.0	=	0.1±0.1	=	0.2±0.1	=	0.1±0.0	0.1±0.0	0.1±0.0	[illegible]	0.1±0.1	=	0.2±0.0	=	0.2±0.1	=
18:4/20:0/20:4 + 18:3/20:0/20:5	0.1±0.1	=	0.1±0.0	=	0.1±0.0	=	0.1±0.0	<	0.1±0.0	<	0.3±0.1	>	0.2±0.1	>	0.1±0.0	=	0.1±0.0	=	0.1±0.0	=	0.1±0.0	<	0.3±0.1	>	0.1±0.0	0.2±0.0	0.1±0.0	[illegible]	0.5±0.1	=	0.1±0.0	=	0.4±0.0	=
18:4/20:0/20:5	0.1±0.1	=	0.1±0.0	=	0.1±0.0	=	0.1±0.0	<	0.2±0.0	=	0.2±0.1	>	0.3±0.0	>	0.1±0.0	=	0.1±0.0	=	0.2±0.0	<	0.2±0.0	=	0.3±0.1	>	0.1±0.0	0.2±0.0	0.2±0.0	[illegible]	0.2±0.0	=	0.3±0.0	=	0.3±0.0	=
18:4/20:4/20:5	0.3±0.2	=	0.2±0.0	=	0.2±0.0	=	0.1±0.1	=	0.2±0.0	=	0.2±0.1	=	0.6±0.1	>	0.2±0.1	=	0.2±0.1	=	0.2±0.1	=	0.2±0.1	<	0.4±0.1	>	0.3±0.0	0.3±0.1	0.4±0.1	[illegible]	0.3±0.1	=	0.7±0.0	=	0.5±0.2	=
20:4/18:4/20:5	0.9±0.4	<	2.4±0.1	<	3.3±0.1	>	2.3±0.6	=	1.6±0.2	>	1.1±0.3	<	1.6±0.3	<	2.5±0.4	<	3.2±0.2	>	2.7±0.1	<	3.5±0.6	>	2.2±0.7	=	0.7±0.2	1.3±0.9	1.2±0.1	[illegible]	1.0±0.2	=	1.9±0.1	=	1.7±0.1	=
20:5/18:4/20:5	0.9±0.6	<	2.6±0.4	<	5.2±0.2	>	3.5±1.4	=	2.3±0.2	>	0.7±0.2	<	2.3±0.6	<	4.9±1.3	=	6.3±0.7	>	4.6±0.6	=	5.1±0.6	>	1.5±0.4	<	1.5±0.4	1.4±0.9	1.4±0.1	[illegible]	0.5±0.1	=	3.5±0.5	=	1.3±0.3	=
20:4/20:3/20:4	+	=	+	=	0.1±0.0	>	+	<	+	<	0.2±0.1	>	+	=	+	=	+	=	+	<	+	<	0.1±0.1	>	+	0.1±0.0	+	[illegible]	0.3±0.1	=	+	=	0.2±0.0	=
20:4/20:3/20:5	0.1±0.0	=	0.1±0.1	=	0.1±0.0	=	0.1±0.0	=	0.1±0.0	<	0.2±0.1	=	0.1±0.0	=	+	<	0.1±0.0	<	0.1±0.0	=	0.2±0.0	=	0.3±0.1	>	0.1±0.0	0.1±0.1	+	[illegible]	0.2±0.0	=	0.1±0.0	=	0.2±0.0	=
20:4/20:4/20:4	0.1±0.0	<	0.3±0.1	=	0.2±0.1	>	0.1±0.0	<	0.1±0.0	<	0.9±0.7	>	0.1±0.0	=	0.2±0.1	=	0.2±0.1	>	0.1±0.0	=	0.1±0.0	<	1.3±0.7	>	0.1±0.1	0.3±0.2	0.1±0.0	[illegible]	1.1±0.5	=	0.1±0.0	=	1.2±0.4	=
20:4/20:5/20:4	0.1±0.0	<	0.5±0.1	=	0.6±0.2	=	0.3±0.1	=	0.3±0.0	=	0.4±0.2	=	0.2±0.0	<	0.4±0.2	=	0.5±0.0	=	0.6±0.1	=	0.7±0.2	=	1.1±0.5	=	0.1±0.0	0.5±0.3	0.1±0.0	[illegible]	0.5±0.1	=	0.2±0.0	=	1.1±0.3	=
20:4/20:4/20:5	+	<	0.1±0.0	=	0.1±0.0	=	0.1±0.1	=	0.1±0.0	=	+	=	0.1±0.0	=	0.1±0.0	<	0.1±0.0	<	0.2±0.0	=	0.3±0.1	=	0.1±0.0	<	0.1±0.0	0.1±0.1	+	[illegible]	0.2±0.1	=	0.1±0.0	=	0.1±0.0	=
20:5/20:4/20:5	+	=	+	=	+	=	+	=	+	<	+	=	0.1±0.0	=	+	=	0.1±0.0	=	0.1±0.0	=	0.1±0.1	>	0.1±0.0	=	0.1±0.0	0.1±0.0	+	[illegible]	0.1±0.0	=	0.1±0.0	=	0.1±0.0	=
18:0/20:5/20:5	0.1±0.0	=	+	=	+	=	+	<	+	<	0.1±0.1	>	+	=	+	=	+	=	+	<	+	=	0.1±0.1	=	0.1±0.0	0.1±0.0	0.1±0.1	[illegible]	0.2±0.2	=	0.1±0.0	=	0.1±0.0	=
20:4/20:5/20:5	0.1±0.0	<	0.5±0.1	=	0.7±0.1	>	0.4±0.2	=	0.3±0.0	>	0.2±0.1	=	0.2±0.1	=	0.3±0.1	<	0.6±0.0	<	0.7±0.0	=	1.0±0.3	>	0.5±0.2	<	0.2±0.0	0.4±0.2	0.1±0.0	[illegible]	0.2±0.1	=	0.3±0.1	=	0.5±0.2	=
20:5/20:5/20:5	0.1±0.0	<	0.2±0.0	<	0.3±0.0	>	0.1±0.1	=	0.1±0.0	=	0.3±0.2	=	0.1±0.0	<	0.2±0.1	=	0.3±0.0	>	0.2±0.1	=	0.3±0.1	=	0.2±0.0	=	0.1±0.0	0.2±0.1	0.1±0.0	[illegible]	0.2±0.0	=	0.2±0.0	=	0.2±0.1	=

Table S4. The plastid-derived lipid molecular species of MGDG, DGDG, SQDG, PG, PE, PC, PI and TAG of various parts of the blades of *Undaria pinnatifida* samples uninfected and infected with the endophyte *Laminariocolax aecidioides* collected in different months. Values given as % of total molecular species (mean ± s.d. for triplicate). "+" – amount less than 0.1%, "-" – not detected. Between columns result of Tukey test and Students t-test (p<0.05, n=3) are shown (">", "<" – statistically significant difference, "=" – no significant changes). "T" – difference between «June» and «February», «S» – difference between uninfected and infected samples.

Lipid	The lower part of the blades of infected *U. pinnatifida* samples												The upper intact part of the blades of infected *U. pinnatifida* samples												The lower part of the blades of uninfected *U. pinnatifida* samples		The lower part of the blades of uninfected *U. pinnatifida* samples				The upper part of the blades of uninfected *U. pinnatifida* samples			
	Nov.		Dec.		Jan.		Feb.		Apr.		Jun.	T	Nov.		Dec.		Jan.		Feb.		Apr.		Jun.	T	Nov.	Jun.	Nov.	S	Jun.	S	Nov.	S	Jun.	S
MGDG	0.8±0.1	=	0.9±0.2	=	0.8±0.3	=	1.0±0.2	=	0.8±0.2	<	1.3±0.2	=	1.2±0.6	<	2.4±0.8	>	0.8±0.1	<	1.3±0.3	>	0.8±0.0	=	1.1±0.3	=	2.6±1.1	1.9±0.3	1.2±0.0	>	1.3±0.4	=	3.7±0.1	>	1.0±0.0	=
DGDG	1.1±0.4	>	0.4±0.2	=	0.8±0.3	=	1.2±0.2	>	0.7±0.2	<	2.2±0.8	>	1.7±0.8	=	1.0±0.3	=	1.0±0.1	=	1.9±1.5	=	1.3±0.3	=	3.1±1.9	=	2.2±1.4	3.2±1.7	1.1±0.0	=	2.3±0.1	=	2.2±0.1	=	1.7±0.5	=
SQDG	80.3±2.0	=	78.8±3.4	=	82.6±1.0	=	82.8±1.2	<	86.0±1.1	>	80.9±2.0	=	78.0±1.9	>	74.1±2.1	=	77.1±1.9	<	83.1±1.8	=	83.0±1.2	>	77.1±2.7	<	79.2±2.0	79.3±1.7	81.0±1.6	=	82.1±5.0	=	76.9±0.2	=	74.0±0.3	=
PG	39.7±2.7	=	42.5±2.9	<	47.9±2.8	=	48.0±4.3	>	38.4±0.6	<	44.4±4.5	=	52.5±0.8	>	48.9±1.2	<	53.5±3.1	<	60.3±1.3	>	54.4±2.9	=	48.5±6.0	<	46.5±2.9	44.2±2.7	41.1±1.8	=	44.2±3.0	=	52.0±2.8		53.1±1.1	=
PE	0.8±0.2	=	0.8±0.1	=	0.9±0.3	=	0.8±0.1	=	0.7±0.1	>	0.3±0.1	<	0.7±0.1	=	1.0±0.4	=	0.7±0.1	<	1.8±0.7	>	0.9±0.3	=	1.4±0.7	=	1.8±0.4	3.2±2.5	1.1±0.2	=	0.3±0.0	=	2.0±0.1	>	1.1±0.9	=
PC	1.1±0.0	>	0.8±0.2	=	0.7±0.1	>	0.5±0.0	<	0.8±0.1	=	1.1±0.3	>	0.8±0.1	=	0.7±0.2	=	0.5±0.1	=	0.6±0.1	=	0.5±0.1	<	0.9±0.2	>	0.9±0.1	0.8±0.4	1.5±0.3	=	1.4±0.1	=	0.9±0.1	=	0.9±0.2	=
PI	1.6±1.2	=	1.5±1.4	=	1.2±1.2	=	0.8±0.4	=	1.3±1.5	=	2.4±1.2	>	1.5±0.2	>	0.4±0.8	=	1.7±1.0	=	2.6±2.8	=	1.4±0.3	>	0.2±0.3	=	1.3±0.6	1.7±1.2	0.7±1.0	=	+	=	1.9±1.8	=	2.3±3.2	=
TAG	38.3±4.5	=	34.0±2.2	>	28.7±0.5	=	28.8±3.1	<	33.0±1.6	<	38.0±1.1	>	35.1±1.9	=	34.2±3.0	>	26.2±0.5	=	26.5±1.0	=	23.9±2.3	<	34.4±2.8	>	46.5±2.9	44.2±2.7	41.1±1.8	=	44.2±3.0	=	52.0±2.8		53.1±1.1	=
